# Epigenetic Priming with Oral Decitabine/Cedazuridine Combined with Nivolumab in Unresectable or Metastatic Mucosal Melanoma: Clinical and Correlative Findings from a Phase Ib/II Trial

**DOI:** 10.3390/cancers18193172

**Published:** 2026-10-01

**Authors:** Jessica S. W. Borgers, Cheng-Chang Wu, Theresa M. Medina, Claire M. Muckle, Elizabeth N. Katsnelson, Phaedra A. Whitty, Luke Mantle, Sapna P. Patel, Dexiang Gao, Kimberly R. Jordan, William A. Robinson, Richard P. Tobin, Martin D. McCarter, Kasey L. Couts

**Affiliations:** 1 Division of Surgical Oncology, Department of Surgery, University of Colorado Anschutz Medical Campus, Aurora, CO 80045, USA; jessica.borgers@cuanschutz.edu (J.S.W.B.); claire.muckle@cuanschutz.edu (C.M.M.); elizabeth.katsnelson@austin.utexas.edu (E.N.K.); richard.tobin@cuanschutz.edu (R.P.T.); martin.mccarter@cuanschutz.edu (M.D.M.); 2 Department of Pediatrics, School of Medicine, University of Colorado Cancer Center Biostatistics Core, University of Colorado Anschutz Medical Campus, Aurora, CO 80045, USA; cheng-chang.wu@cuanschutz.edu (C.-C.W.); dexiang.gao@cuanschutz.edu (D.G.); 3 Division of Medical Oncology, Department of Medicine, University of Colorado Anschutz Medical Campus, Aurora, CO 80045, USA; theresa.medina@cuanschutz.edu (T.M.M.); phaedra.whitty@cuanschutz.edu (P.A.W.); luke.mantle@cuanschutz.edu (L.M.); sapna.p.patel@cuanschutz.edu (S.P.P.); william.robinson@cuanschutz.edu (W.A.R.); 4 Department of Ophthalmology, University of Colorado Anschutz Medical Campus, Aurora, CO 80045, USA; 5 Department of Immunology and Microbiology, University of Colorado Anschutz Medical Campus, Aurora, CO 80045, USA; kimberly.jordan@cuanschutz.edu

**Keywords:** mucosal melanoma, decitabine/cedazuridine, nivolumab, RIG-I, viral mimicry

## Abstract

**Background/Objectives:** Mucosal melanoma is a rare, aggressive melanoma subtype with lower response rates to immune checkpoint inhibitors than cutaneous melanoma. We previously identified reduced expression of innate immune pathogen-sensing pathway genes in mucosal melanoma, including components of the retinoic acid-inducible gene-I (RIG-I) pathway, and found that decitabine can induce re-expression of these immune genes and tumor-associated antigens. This study evaluated epigenetic immune priming with oral decitabine/cedazuridine (DEC-C) combined with nivolumab in patients with unresectable or metastatic mucosal melanoma. **Methods:** NCT05089370 was an open-label, single-center, single-arm phase Ib/II trial. Patients received DEC-C (35 mg decitabine/100 mg cedazuridine) orally for five days followed by nivolumab 480 mg every four weeks. The primary endpoint was safety and determination of the recommended phase II dose (RP2D). Secondary endpoints included objective response rate (ORR) and survival outcomes. Exploratory endpoints assessed immune and epigenetic correlates. **Results:** Eight patients were enrolled. Treatment-related adverse events occurred in seven patients (88%), with dose-limiting toxicities observed in three (38%). The RP2D was not established due to premature trial closure. At data cut-off, two patients remained on treatment with ongoing partial responses. The ORR was 25% (95% CI, 3.2–65), median progression-free survival was 3.0 months (95% CI, 2.1–not reached), and median overall survival was 7.2 months (95% CI, 4.4–not reached). Treatment significantly decreased global DNA methylation and induced RIG-I pathway genes in circulating immune cells. **Conclusions:** DEC-C plus nivolumab was associated with frequent treatment-related toxicities consistent with the known safety profiles of decitabine and nivolumab and demonstrated on-target epigenetic and immune activity in circulating cells. Although two partial responses were observed, the small sample size and wide confidence interval preclude conclusions regarding efficacy. These findings support future biomarker-integrated studies evaluating epigenetic immune-priming combinations in mucosal melanoma, with prospective optimization of dose, schedule, and treatment setting.

## Simple Summary

Mucosal melanoma is a rare and aggressive melanoma subtype with lower response rates to immunotherapy than cutaneous melanoma, creating a need for new combination treatment strategies. This study evaluated oral decitabine/cedazuridine, an epigenetic drug which alters DNA methylation and activates innate immune signaling pathways, in combination with anti-PD-1 immunotherapy nivolumab in patients with unresectable or metastatic mucosal melanoma. The trial closed early and enrolled only eight patients, reflecting the challenges of conducting prospective clinical trials in rare melanoma. Despite these limitations, the study provided important clinical and correlative insight, including an encouraging clinical signal in some patients. Treatment produced measurable biological effects in circulating immune cells, including reduced DNA methylation and increased expression of innate immune pathway genes. These findings support further investigation of biomarker-driven epigenetic immune-priming combinations in mucosal melanoma and other immunotherapy-resistant tumors characterized by impaired innate immune signaling.

## 1. Introduction

Mucosal melanoma is a rare and aggressive melanoma subtype arising from mucosal surfaces of the head and neck, anorectal, and genitourinary regions [1,2]. Outcomes for patients with mucosal melanoma are significantly worse than for those with cutaneous melanoma, with 5-year survival rates of approximately 25% and 81%, respectively [1]. This disparity is partly explained by later-stage diagnosis, challenges in surgical resection, and resistance to standard melanoma therapies, including immunotherapy [3,4,5,6]. Whereas patients with metastatic cutaneous melanoma achieve objective response rates of approximately 60% with combination anti-PD-1 and anti-CTLA-4 therapy and 41–52% with anti-PD-1 monotherapy, refs. [7,8] response rates in mucosal melanoma are substantially lower at 32% and 22%, refs. [4,5,6] respectively, despite similar rates of immune-related adverse events [4,5,9,10,11]. Complete responses are also significantly less frequent in patients with mucosal melanoma compared to those with cutaneous melanoma [12]. Collectively, these findings underscore the need for mechanism-based combination strategies that improve immunotherapy responsiveness in patients with mucosal melanoma.

Previously, we demonstrated that the retinoic acid-inducible gene-I (RIG-I) innate immune pathogen-sensing (IIPS) pathway is suppressed in mucosal melanoma compared with cutaneous melanoma [13]. In cutaneous melanoma, RIG-I pathway activity is associated with response to anti-PD-1 therapy, enhanced antigen presentation, and a favorable immune cell profile in the tumor microenvironment [14,15,16]. High RIG-I pathway activity is associated with increased MHC class I and II expression, greater frequency and activity of CD4^+^ and CD8^+^ T cells, and reduced abundance and suppressive function of myeloid-derived suppressor cells (MDSCs) and regulatory T cells (Tregs) [16]. Together, these findings suggest that low RIG-I pathway gene expression in mucosal melanoma may contribute to impaired antitumor immunity and intrinsic resistance to PD-1 blockade.

Although direct RIG-I agonists are under development, they are not yet clinically available. In contrast, hypomethylating agents (HMAs) that inhibit DNA methyltransferase 1 (DNMT1) can induce global hypomethylation and endogenous retroviral/retroelement expression resulting in a viral mimicry state that increases expression of IIPS pathway genes, tumor antigens, and natural killer (NK) cell ligands [17,18]. Because viral mimicry-associated innate immune programs have been implicated in antitumor immunity across multiple cancer types, epigenetic priming with HMAs represents a broadly relevant strategy to enhance immune checkpoint blockade, including in mucosal melanoma. Consistent with this mechanism, preclinical and clinical studies across multiple solid tumors indicate that epigenetic priming with HMAs can enhance immunotherapy efficacy by favorably remodeling the tumor microenvironment, refs. [14,15,16,19,20] supporting DNMT1 inhibition as a potential strategy to sensitize mucosal melanoma to PD-1-based therapy.

Of particular interest is the HMA decitabine, a cytidine-nucleoside analog that is incorporated into DNA during the S-phase of the cell cycle. Decitabine covalently binds DNMT1, causing its degradation and resulting in global DNA hypomethylation and broad changes in gene expression [21]. In our prior preclinical study of mucosal and other rare melanoma, decitabine was a potent inducer of IIPS genes and tumor-associated antigens, providing the rationale for its use as an epigenetic priming strategy to restore antitumor immune signaling in this disease [13].

Historically, decitabine required intravenous administration because it is rapidly deaminated by cytidine deaminase in the gastrointestinal tract. More recently, the oral fixed-dose formulation decitabine/cedazuridine (DEC-C), which combines decitabine with an acid-stable cytidine deaminase inhibitor, was developed to increase oral bioavailability and enable more convenient outpatient administration [22,23]. DEC-C is FDA-approved for the treatment of myelodysplastic syndrome and it is currently being investigated in patients with solid tumors [24]. Here, we report clinical and correlative findings from a pilot phase Ib trial evaluating oral DEC-C plus nivolumab as a mechanism-based epigenetic immune-priming combination strategy in patients with unresectable or metastatic mucosal melanoma.

## 2. Materials and Methods

### 2.1. Study Population

Patients were eligible for enrollment if they were ≥18 years of age and had histologically confirmed, unresectable or metastatic stage III or stage IV mucosal melanoma arising from the oral cavity (excluding the lip), nasopharynx, vagina/vulva, or rectum. Patients were required to have at least one measurable lesion according to Response Evaluation Criteria in Solid Tumors (RECIST) version 1.1, an Eastern Cooperative Oncology Group (ECOG) performance status of 0 or 1, and adequate organ function. Patients with treated brain metastases were eligible if lesions were radiographically stable for at least four weeks on magnetic resonance imaging. Prior immune checkpoint inhibitor (ICI) therapy was permitted if study treatment was initiated at least 30 days after completion of prior ICI therapy (excluding adjuvant anti-PD-1 or anti-CTLA-4 therapy). Patients with leptomeningeal disease, active infection or autoimmune disease, or requiring >10 mg/day of prednisone or equivalent were excluded. Complete eligibility criteria are provided in the full protocol (Supplementary File S1).

### 2.2. Study Design and Treatment

**Figure 1:**
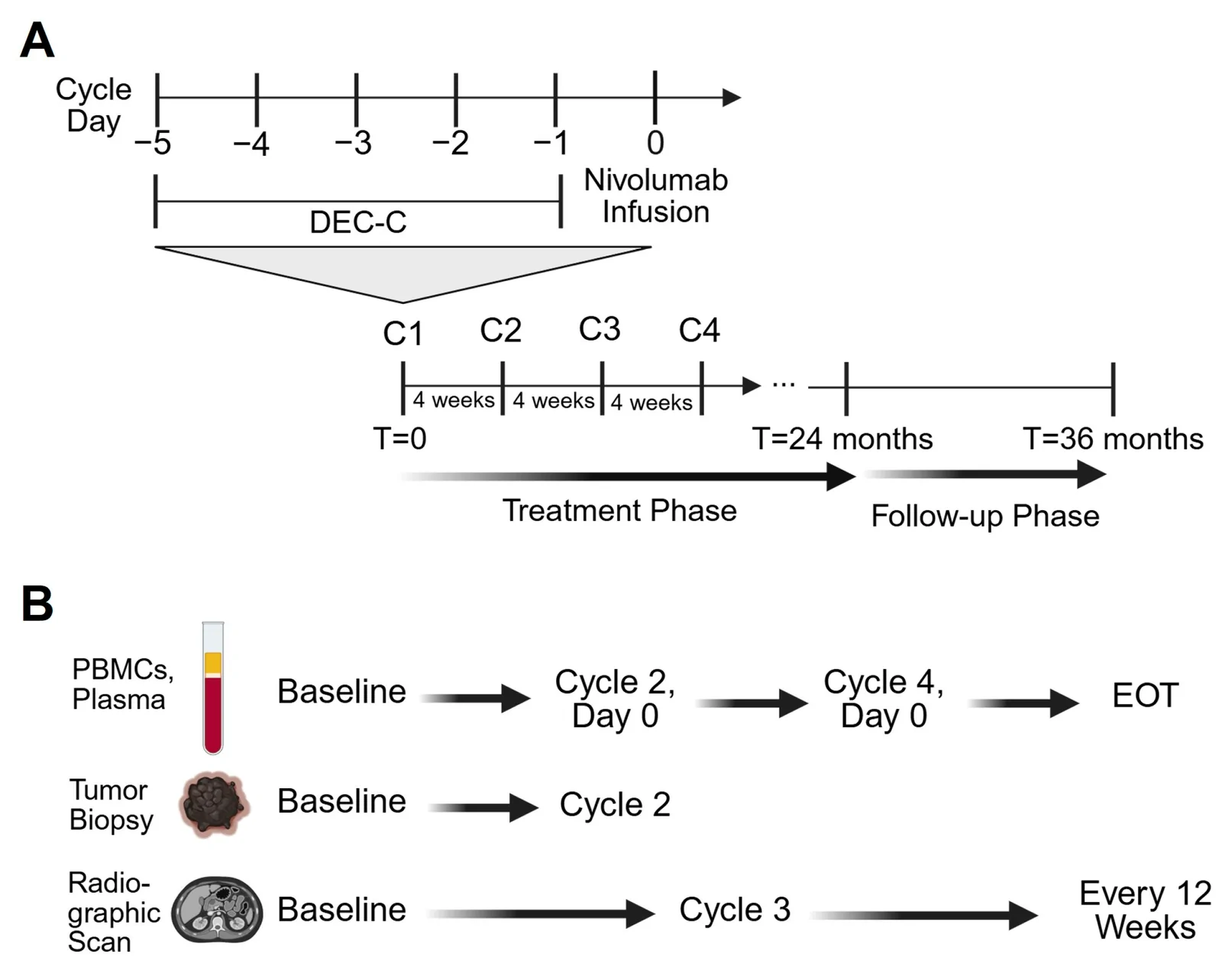
Study design and biospecimen collection schema. (**A**) Treatment schema for the phase Ib/II trial combining oral decitabine/cedazuridine (DEC-C) with nivolumab. DEC-C was administered once daily for five consecutive days prior to nivolumab infusion, followed by nivolumab 480 mg intravenously every four weeks. Dose reduction of DEC-C to a three-day priming schedule was permitted in the event of dose-limiting toxicities. (**B**) Schematic of prespecified biospecimen collection and imaging timepoints. Peripheral blood samples were collected at baseline, during treatment prior to nivolumab administration (cycles 2 and 4), and at disease progression for correlative immune and epigenetic analyses. Tumor biopsies were obtained at baseline and on treatment when feasible. Radiographic tumor assessments were performed at baseline and every 12 weeks thereafter per standard of care. Created in BioRender. Couts, K. (2026) https://BioRender.com/v9tk0pq.

NCT05089370 was an open-label, single-center, single-arm phase Ib/II trial designed to evaluate the safety and efficacy of the oral decitabine/cedazuridine (DEC-C) combined with nivolumab in patients with mucosal melanoma at the University of Colorado Cancer Center. The phase Ib portion was designed to determine the recommended phase II dose (RP2D) using a time-to-event Bayesian optimal interval design (TITE-BOIN), with patients enrolled in cohorts of three. All patients received standard-of-care nivolumab 480 mg intravenously every four weeks (Day 0). At dose level 1, DEC-C (35 mg decitabine/100 mg cedazuridine) was administered orally once daily at home on days −5, −4, −3, −2, and −1 of each nivolumab cycle (Figure 1A). In the event of dose-limiting toxicities (DLTs), defined as grade 3 or 4 toxicities attributed to DEC-C, both DEC-C and nivolumab were withheld until resolution to grade 1 or lower. DEC-C was then resumed on a reduced dosing schedule of three consecutive days per cycle (days −3, −2, and −1). Planned treatment duration was up to 24 months, followed by a 12-month follow-up period, for a total planned study duration of 36 months. Toxicities were reviewed by an independent data and safety monitoring committee.

Up to nine patients were planned for treatment at the RP2D during the phase Ib dose-finding portion of the trial. If at least three responses were observed, the study would proceed to the second stage. A minimum of 19 patients was required to preliminarily assess efficacy and further characterize safety at the RP2D. However, sponsor funding was discontinued for this and other DEC-C solid tumor trials for programmatic reasons unrelated to safety, efficacy, or interim analysis of the present study; therefore, the trial closed before completion of the phase Ib portion. Accordingly, we report safety, efficacy, and correlative analyses from the eight patients treated in the dose-finding phase in a non-pre-specified analysis.

This study was approved by the Colorado Multiple Institutional Review Board on 15 September 2021 (approval number 21-3518) and was conducted in accordance with the Declaration of Helsinki and the International Council for Harmonization Good Clinical Practice guidelines. Written informed consent was obtained from all participants. A completed CONSORT-DEFINE checklist is provided as Supplementary File S2.

### 2.3. Endpoints

The primary endpoint was safety and tolerability of DEC-C in combination with nivolumab and determination of the RP2D of DEC-C. Adverse events (AEs) were assessed by treating physicians according to the National Cancer Institute Common Terminology Criteria for Adverse Events (CTCAE) version 5.0. Secondary endpoints included objective response rate (ORR) and progression-free survival (PFS), assessed by the investigator according to RECIST v1.1, and overall survival (OS). The efficacy analysis population included all patients who received at least one dose of DEC-C. Objective response was defined as complete or partial response as best overall response. PFS was defined as the time from treatment initiation to first documented disease progression or melanoma-related death, with patients without an event censored at last follow-up. OS was defined as the time from treatment initiation to death from any cause, with patients alive at data cut-off censored at date of last follow-up. Exploratory endpoints evaluated the effects of DEC-C combined with nivolumab on circulating and tumor immune cell profiles, global DNA methylation and RIG-I pathway gene expression in circulating immune cells, and circulating cytokines.

### 2.4. Procedures and Assessments

Patients were evaluated prior to each nivolumab infusion. Safety assessments included review of AEs, symptom-directed physical examination, ECOG performance status, and laboratory testing. Research blood samples were collected at baseline, prior to nivolumab administration during cycles 2 and 4, and at the time of disease progression (Figure 1B). Plasma was isolated by centrifugation at 340× *g* for 10 min, and peripheral blood mononuclear cells (PBMCs) were isolated by Ficoll (Cytiva) density gradient centrifugation. Radiographic tumor assessments were performed at baseline and every 12 weeks thereafter per standard-of-care and evaluated according to RECIST v1.1. When feasible, tumor biopsies were obtained prior to treatment initiation and around cycle 2. Biopsy specimens were enzymatically digested using Liberase TL (Roche Diagnostics GmbH, Mannheim, Germany) and tumor cell suspensions were cryopreserved until analysis [25].

### 2.5. Methylation Assay

Global DNA methylation levels were assessed in PBMCs collected at baseline and on treatment by quantifying LINE-1 methylation using quantitative real-time PCR (qRT-PCR) on bisulfite-treated genomic DNA. Genomic DNA (gDNA) was purified from PBMCs using the DNeasy Blood and Tissue Kit (Qiagen, Inc., Germantown, MD, USA). A total of 1 µg gDNA was subjected to bisulfite conversion using the EpiTect Bisulfite Kit (Qiagen, Inc.). LINE-1 DNA was quantified in triplicate by qRT-PCR using primer sets specific for unmethylated LINE-1 (forward, 5′-TGTGTGTGAGTTGAAGTAGGGT-3′; reverse, 5′-ACCCAATTTTCCAAATACAACCATCA-3′) or methylated LINE-1 (forward, 5′-CGCGAGTCGAAGTAGGGC-3′; reverse, 5′-ACCCGATTTTCCAAATACGACCG-3′). For each sample, ΔCt was calculated as Ct(methylated)-Ct(unmethylated) to derive a methylated-to-unmethylated ratio. For each patient, this ratio was normalized to the corresponding baseline sample.

### 2.6. Gene Expression Analysis

Gene expression was analyzed in PBMCs collected at baseline and during treatment using qRT-PCR. Total RNA was purified from PBMCs using the RNeasy Plus Kit (Qiagen, Inc.) with on-column DNase digestion. A total of 1 μg of RNA was reverse-transcribed into cDNA using the Verso cDNA Synthesis Kit with random hexamer primers (Thermo Fisher Scientific, Waltham, MA, USA). cDNA was diluted 1:10, and 1 μL per reaction was used to quantify gene expression in triplicate by qRT-PCR with PowerUP SYBR Green Master Mix (Thermo Fisher Scientific). Primer sequences were as follows: *GAPDH* forward, 5′-CCCTTCATTGACCTCAACTACA-3′; *GAPDH* reverse, 5′-GTCATGAGTCCTTCCACGATAC-3′; *IFI6*, forward 5′-CGCTTTTCTTGTGCTACCTG-3′; *IFI6* reverse, 5′-TGTCCGAGCTCTCCGAG-3′; *IFI27* forward, 5′-CCTCTGCTCTCACCTCATC-3′; *IFI27* reverse, 5′-CACAACTCCTCCAATCACAAC-3′; *IFI44L* forward, 5′-ATATCACCAGCATAACCGAGC-3′; *IFI44L* reverse, 5′-TGTCATCCATGCACAGTCC-3′. Gene expression was calculated relative to *GAPDH* using the 2^-ΔCT method, where ΔCT represents the difference between the target gene Ct value and the *GAPDH* Ct value.

### 2.7. Flow Cytometric Analysis

Flow cytometric analysis of PBMCs and tumor specimens collected pre-, on-, and post-treatment was performed as described in Supplementary Table S1. Markers were chosen to identify well-described immune cell subsets in the circulation and tumors and to characterize markers of cellular function associated with antitumor immunity, including cytotoxicity, immunosuppression, and immune checkpoint expression [25,26,27,28,29,30]. Stained samples were analyzed using a Cytek Aurora spectral flow cytometer (Cytek Biosciences, Inc., Fremont, CA, USA) at the University of Colorado Cancer Center Flow Cytometry Shared Resource. Data were analyzed using FlowJo software (version 10.10.0).

### 2.8. Circulating Cytokine Analysis

Serial plasma samples were analyzed for concentrations of IL-1β, IL-6, IL-8, tumor necrosis factor-α (TNF-α), interferon (IFN)-β, IFN-γ, and IFN-α2a using a custom kit (Meso Scale Diagnostics, LLC, Rockville, MD, USA), according to the manufacturer’s protocol. Cytokine concentrations were quantified using a QuickPlex SQ 120 instrument (Meso Scale Discovery) in the Human Immune Monitoring Shared Resource at the University of Colorado School of Medicine.

### 2.9. Statistical Analysis

A TITE-BOIN design was used in the phase Ib portion of the trial to identify the maximum tolerated dose (MTD) and RP2D, with a target DLT rate of ϕ = 0.30 and underdosing and overdosing boundaries of 0.236 and 0.359, respectively, based on a 28-day DLT assessment window. For the planned phase II portion, the original power calculation was based on Simon’s two-stage design to evaluate ORR. The study hypothesized an ORR of 43%, representing a 23% absolute improvement over the historical ORR of ~20% observed with anti-PD-1 therapy in mucosal melanoma [4,5,9,10,11]. Under this design, the null hypothesis (ORR = 20%) would be rejected if three or more responses were observed among the first 11 patients, with planned enrollment continuing until at least 19 patients were treated at the RP2D. This design provided a one-sided type I error rate of 0.10 and 80% power when the true response rate was 43%.

The study was discontinued before completion of the phase Ib portion. Therefore, the planned phase Ib MTD determination and phase II efficacy analyses among patients treated at the RP2D were not performed. Instead, all treated patients were included in the analyses reported here. Demographic, baseline, and safety data were summarized descriptively. ORR was calculated with exact 95% confidence intervals (CIs). Median PFS and OS, with corresponding 95% CIs, were estimated using the Kaplan–Meier method. Exploratory endpoints were log-transformed using the natural logarithm, as appropriate, and summarized descriptively. Values of zero were replaced with a pseudo-count of 0.01 for log transformation. Changes from baseline to on-treatment were assessed using paired *t*-tests. All statistical tests were two-sided, with a significance level of 0.05. Statistical analyses were performed using R (version 4.3.1) and GraphPad Prism (version 10.5.0).

## 3. Results

### 3.1. Baseline Patient Characteristics

Between 20 June 2022 and 22 July 2024, ten patients were screened for eligibility, of whom eight (Table 1) were enrolled and received at least one dose of study treatment. All enrolled patients were female (100%), with a median age of 64 years (range, 34–71). Most patients had stage IV disease (6/8, 75%), and primary tumor sites included anorectal (*n* = 4, 50%), vulvovaginal (*n* = 3, 38%), and oral (*n* = 1, 13%) mucosal melanoma. At time of enrollment, five patients (63%) had received prior systemic therapy for unresectable or metastatic disease, with a median of three prior lines of treatment in this setting (range, 1–4). All five patients had progressed on either single-agent anti-PD-1 therapy or an anti-PD-1-based combination regimen with ipilimumab or relatlimab prior to enrollment. Patients 003 and 009 were ICI-naïve, while patient 010 had received adjuvant nivolumab two years before disease recurrence and trial enrollment. Baseline patient characteristics are summarized in Table 1.

### 3.2. Safety and Tolerability of DEC-C Plus Nivolumab

**Figure 2:**
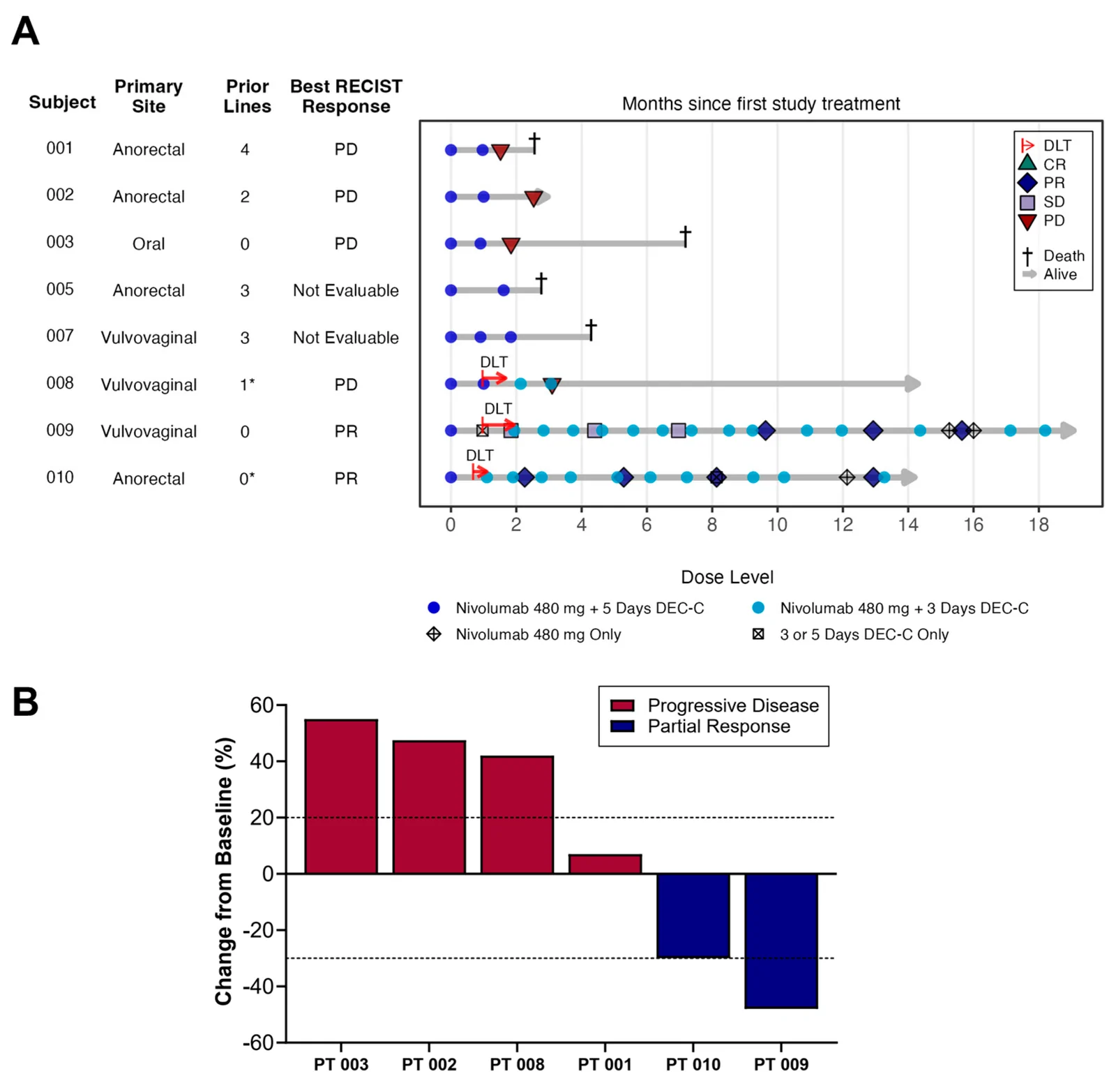
Overview of treatments and responses. (**A**) Swimmer’s plot showing the location of the mucosal site of the primary tumor, number of prior lines of systemic therapy for unresectable stage III/stage IV disease, best overall response according to RECIST +v1.1, response over time, number of treatments cycles received, duration of follow-up, and vital status. * Indicates prior neoadjuvant ipilimumab + nivolumab (008) and adjuvant nivolumab (010). (**B**) Waterfall plot showing the best overall response presented as change in the sum of target lesions from baseline.

Safety outcomes and the most common treatment-related AEs are summarized in Table 2. Seven of eight patients (88%) experienced at least one treatment-related AE. The most frequently reported AEs of any grade, occurring in ≥20% of patients, were nausea (62.5%); fatigue (50%); constipation, hypoxia, decreased neutrophil count, and pain (37.5% each); and abdominal pain, anemia, febrile neutropenia, flu-like symptoms, and decreased white blood cell count (25% each). All patients with treatment-related AEs experienced at least one grade 3 or 4 event. Grade 3–4 AEs occurring in more than one patient included hypoxia (*n* = 3), febrile neutropenia (*n* = 2), decreased neutrophil count (*n* = 2), and pain (*n* = 2). Six AEs in four patients (50%) met protocol-specified criteria for serious adverse events (SAEs), including abdominal pain and febrile neutropenia (*n* = 2 each), and hypoxia, pain, rectal pain, and cancer-related pain (*n* = 1 each). Three patients (38%) experienced a DLT, consisting of grade 3 febrile neutropenia and grade 3 or 4 decreased neutrophil count, which are known adverse effects of DEC-C [31]. In these cases, DEC-C dose-reductions from five to three days per cycle were required (Figure 2A). No grade 5 AEs were observed. The RP2D was not established due to premature trial closure.

### 3.3. Clinical Outcomes with DEC-C Plus Nivolumab

The median number of completed treatment cycles with DEC-C plus nivolumab was 2.5 (interquartile range [IQR] 2.0–8.5), corresponding to a median duration of treatment of 1.7 months (IQR, 1.0–8.2). Reasons for treatment discontinuation included radiographically confirmed disease progression (*n* = 3, 50%), patient withdrawal (*n* = 2, 33%), and physician decision (*n* = 1, 17%). At the data cut-off of September 30, 2025, with a median follow-up of 5.7 months (IQR, 2.9–14.2) for the entire cohort, two patients remained on study treatment with ongoing partial responses (duration of response 6.0 and 10.7 months at data cutoff). Four patients had RECIST-defined progressive disease (PD) as their best overall response, and two patients experienced clinical progression without confirmatory imaging, resulting in an ORR of 25% (95% CI, 3.2–65). Given the small sample size and wide confidence interval, the observed ORR is descriptive and should not be interpreted as evidence of clinical efficacy. Patient-level treatment duration and response outcomes are summarized in Figure 2, including duration of treatment and response over time (Figure 2A). Best radiographic responses are shown in the waterfall plot (Figure 2B), which demonstrates tumor regression exceeding the RECIST threshold for partial response in two patients, while the remaining evaluable patients exhibited stable or progressive disease as their best overall response.

### 3.4. Global DNA Hypomethylation in Circulating Immune Cells

**Figure 3:**
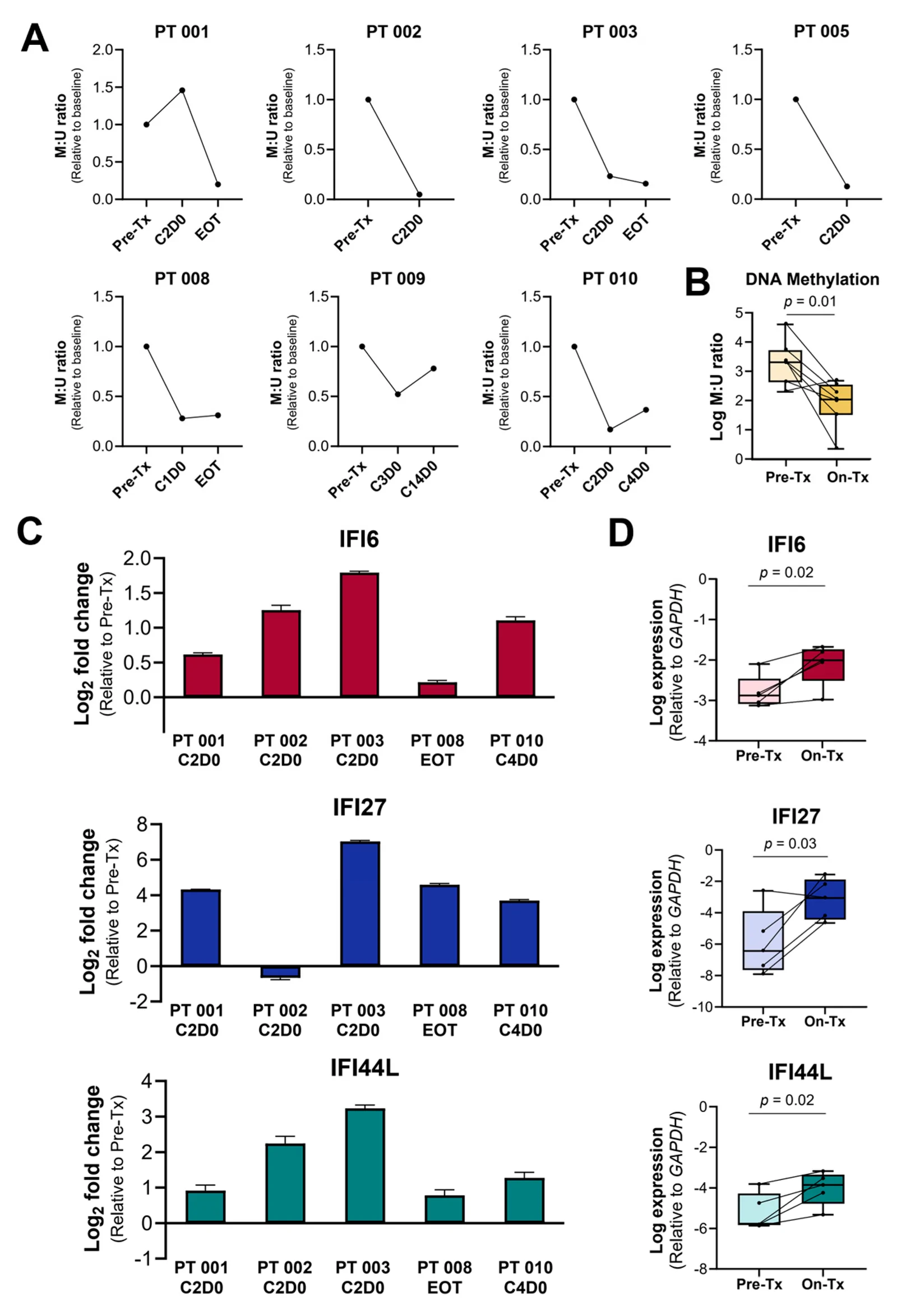
DEC-C induces global DNA hypomethylation and RIG-I pathway gene expression in PBMCs. (**A**) Global DNA methylation levels were assessed in PBMCs from seven evaluable patients using LINE-1 methylation analysis. For each patient, ratios of methylated to unmethylated (M:U) DNA are shown over time and normalized to the baseline sample (day 0). Time points correspond to on-treatment and end-of-treatment blood draws, expressed as days from baseline collection. (**B**) Paired analysis of global DNA methylation in PBMCs comparing pre-treatment (Pre-Tx) and on-treatment (On-Tx) samples across evaluable patients. Each line represents an individual patient with matched samples. M:U ratios are shown on a log scale. (**C**) Expression of RIG-I pathway target genes *IFI6*, *IFI27*, and *IFI44L* was measured by quantitative RT-PCR in matched Pre-Tx and On-Tx- PBMC samples from five patients. Gene expression is shown as log_2_ fold change from baseline, relative to *GAPDH*. On-treatment samples were obtained at cycle 2 day 0 (C2D0), cycle 4 day 0 (C4D0), or end of treatment (EOT), depending on sample availability. Bars represent mean ± SEM of technical triplicates. (**D**) Paired analysis of *IFI6*, *IFI27*, and *IFI44L* expression in PBMCs comparing baseline and on-treatment samples. Each line represents an individual patient with matched samples. Expression values are shown on a log scale relative to *GAPDH*.

Global DNA methylation was evaluated in PBMCs using LINE-1 methylation qRT-PCR analysis. Paired pre and on-treatment samples were available from seven patients. Across evaluable patients, treatment with DEC-C plus nivolumab was associated with a consistent reduction in LINE-1 methylation during therapy, as reflected by a decreased methylated-to-unmethylated LINE-1 ratio normalized to baseline (Figure 3A). Decreases in LINE-1 methylation were observed early during treatment and were sustained across multiple on-treatment time points in patients with longitudinal sampling (Figure 3B). These findings demonstrate on-target epigenetic activity of DEC-C in circulating immune cells in patients with mucosal melanoma.

### 3.5. Induction of RIG-I Pathway Target Genes in Circulating Immune Cells

To determine whether epigenetic priming was associated with induction of RIG-I innate immune pathway genes, expression of downstream target genes was assessed in PBMCs using qRT-PCR. Matched baseline and on-treatment RNA samples were available from five patients. Expression of *IFI6*, *IFI27*, and *IFI44L*, genes previously identified as RIG-I pathway genes induced by decitabine in mucosal melanoma preclinical models, ref. [13] was evaluated relative to baseline.

Across evaluable patients, treatment with DEC-C plus nivolumab was associated with increased expression of all three RIG-I pathway target genes compared with pre-treatment (Figure 3C,D). Induction of *IFI6*, *IFI27*, and *IFI44L* was observed across patients despite variability in sampling time points. Together, these findings indicate that epigenetic priming with DEC-C is associated with transcriptional induction of RIG-I pathway genes in circulating immune cells in patients with mucosal melanoma.

### 3.6. Flow Cytometric Immune Profiling

**Figure 4:**
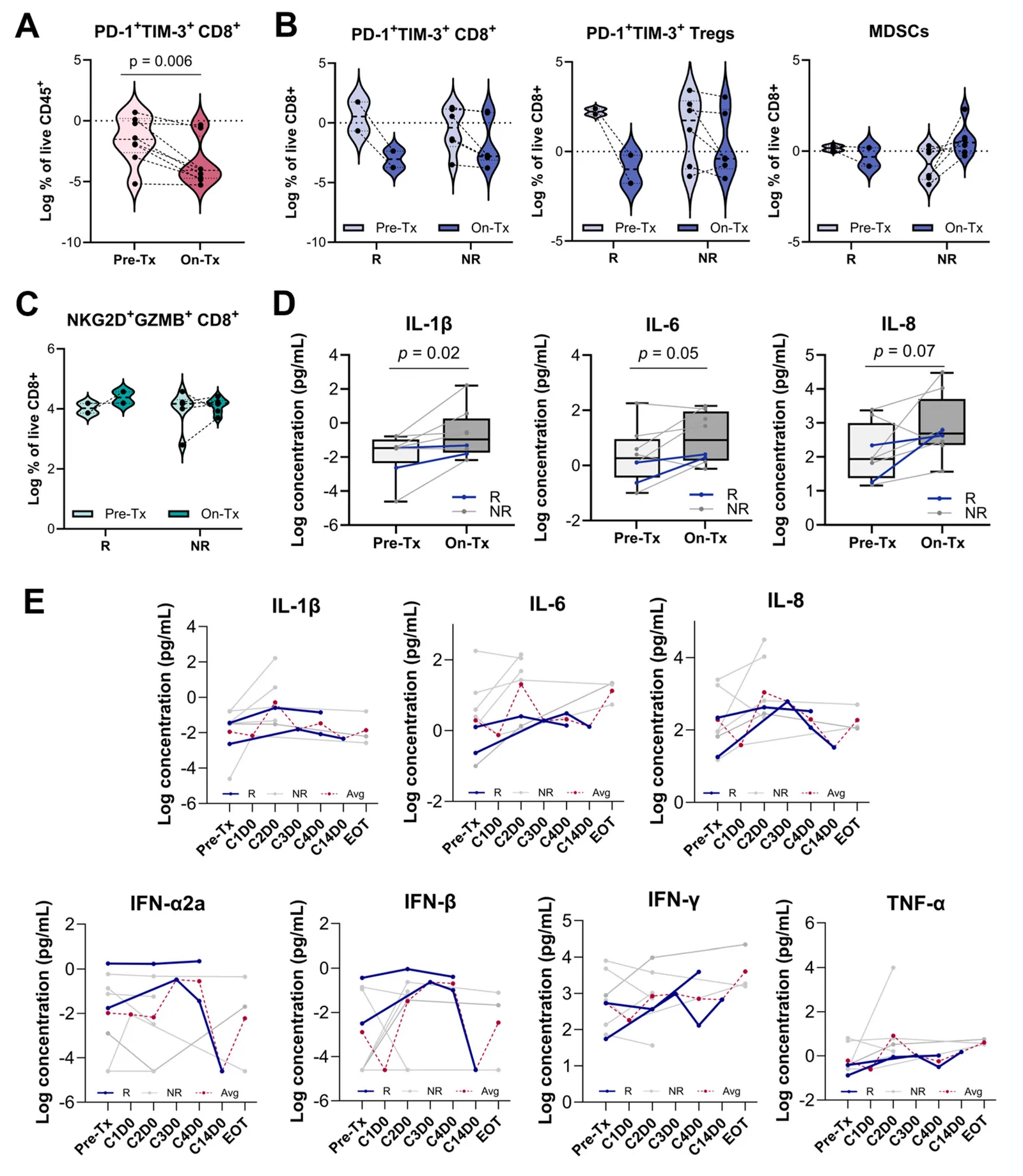
Circulating immune cell and cytokine analyses during treatment with DEC-C plus nivolumab. (**A**–**C**) Flow cytometric analysis of translationally relevant circulating immune cell subsets. (**A**) Comparisons of the frequency PD-1^+^TIM-3^+^ CD8^+^ T cells between samples collected at baseline and on-treatment in all patients. (**B**,**C**) Exploratory descriptive visualization of immune cell changes by response status. Because only two responders were observed, no formal statistical comparisons were performed. (**D**,**E**) Longitudinal analysis of circulating plasma cytokines. (**D**) Plasma cytokines with significant changes in levels during treatment, and (**E**) all cytokines at baseline and serial on-treatment time points. All values are log-transformed.

Flow cytometric analysis of paired pre- and on-treatment PBMCs demonstrated a significant decrease in the frequency of CD8^+^ T cells expressing markers of exhaustive differentiation (PD-1^+^TIM-3^+^) during treatment (Figure 4A). No statistically significant differences were observed between pre- and on-treatment samples in the circulating frequencies of total T cells, B cells, NK cells, Tregs, or MDSCs (Supplementary Table S2). Given that only two patients achieved objective responses, immune profiling by response status was considered exploratory and descriptive, and no formal statistical comparisons were performed. Descriptively, the two responding patients had decreased frequencies of PD-1^+^TIM-3^+^ CD8^+^ T cells, PD-1^+^TIM-3^+^ Tregs, and MDSCs on-treatment (Figure 4B), accompanied by a trend toward increased highly cytotoxic NKG2D^+^GZMB^+^ CD8^+^ T cells (Figure 4C). In contrast, while nonresponding patients demonstrated a similar trend of increased NKG2D^+^GZMB^+^ CD8^+^ T cells, this was accompanied by a trend of increased circulating MDSCs (Supplementary Table S3).

Because only three patients had matched pre- and on-treatment tumor biopsies, including two nonresponders and one responder, analyses of intratumoral immune cell populations were descriptive. In tumor samples, similar trends were observed, with decreased MDSC frequencies in the responding tumor and increased MDSCs in nonresponding tumors. Reductions in the frequencies of PD-1^+^TIM-3^+^Tregs and PD-1^+^TIM-3^+^CD8^+^ T cells were more pronounced in responding compared with nonresponding tumors, whereas the frequency of NKG2D^+^GZMB^+^ CD8^+^ T cells increased across all tumors analyzed (Supplementary Tables S4 and S5).

### 3.7. Circulating Cytokine Analysis

Analysis of plasma samples demonstrated a significant increase in IL-1β levels when comparing the first on-treatment sample with baseline (*p* = 0.02), with trends toward increased IL-6 (*p* = 0.05), and IL-8 (*p* = 0.07) (Figure 4D). No significant changes were observed in plasma levels of TNF-α, IFN-β, IFN-γ, or IFN-α2a. A full overview of plasma cytokine levels over the course of treatment is provided in Figure 4E and Supplementary Table S6. Owing to the small number of patients, no statistically significant differences in cytokine levels were detected between responding and nonresponding patients (Supplementary Table S7).

## 4. Discussion

Although outcomes for patients with cutaneous melanoma have improved substantially with immune checkpoint inhibitors, refs. [32,33] patients with advanced mucosal melanoma continue to experience low response rates to these therapies [4,5,9,10,11] and poor survival outcomes, ref. [1] representing a substantial unmet clinical need. Preclinical studies and emerging clinical strategies are beginning to explore approaches to overcome intrinsic resistance to immunotherapy in mucosal melanoma [34]. One such approach is epigenetic priming using HMAs to restore innate immune signaling pathways required for effective antitumor immunity [13]. In this context, we conducted an open-label, single-center, single-arm pilot trial evaluating nivolumab combined with the oral HMA decitabine/cedazuridine (DEC-C) in patients with unresectable or metastatic mucosal melanoma. Although premature study closure prevented determination of the RP2D, this study provides clinical and correlative evidence that DEC-C plus nivolumab is associated with systemic epigenetic and immune modulation in patients with mucosal melanoma. The small sample size, wide confidence interval, and nonrandomized design preclude conclusions regarding efficacy, but the findings may inform the design of future biomarker-integrated studies of epigenetic priming in this disease. Thus, this study addresses a key challenge in rare melanoma combination therapy: developing mechanism-based immunotherapy combinations in patient populations where prospective clinical trials are difficult to complete, while using integrated correlative biomarkers to determine whether the intended biological effects are achieved.

A growing body of evidence supports a critical role for IIPS pathways, including the RIG-I pathway, in shaping antitumor immunity and response to ICI therapies [14,15,16]. Our recent data demonstrate that mucosal melanomas have lower expression of RIG-I and IIPS pathway genes compared with cutaneous melanoma, a feature associated with immune exclusion and poor response to PD-1 blockade [13]. Among several drug-repurposing strategies evaluated to increase IIPS gene expression in mucosal melanoma preclinical models, HMAs emerged as the most effective class [13]. Although HMAs can activate IIPS-associated transcriptional programs, our work and that of others have shown that decitabine induces these genes more robustly and reproducibly than other HMAs, such as 5-azacytidine [13,17]. Several early-phase clinical studies in other solid tumors have explored combinations of HMAs with ICIs, demonstrating biological activity and, in some cases, preliminary clinical benefit [35,36]. Most prior studies used intravenously administered HMAs, whereas the development of oral DEC-C enables a more practical epigenetic immune-priming strategy that can be readily integrated with immunotherapy. Collectively, these observations provided the rationale for evaluating oral DEC-C combined with nivolumab in patients with advanced mucosal melanoma. Although evaluated here in mucosal melanoma, this strategy is based on a broader body of evidence implicating impaired innate immune sensing and defective viral mimicry-associated transcriptional programs as mechanisms of tumor immune evasion across melanomas and other cancer types.

In this pilot study, DEC-C combined with nivolumab was associated with frequent treatment-related toxicity, including grade 3–4 events in all patients who experienced treatment-related AEs. These toxicities were consistent with the known safety profiles of decitabine and nivolumab, and no grade 5 AEs or unexpected safety signals were observed [31,37]. DLTs were primarily hematologic and aligned with the established safety profile of decitabine. Patients who required DEC-C dose modification were able to continue treatment with a shortened three-day priming schedule, and partial responses were observed in two patients receiving the reduced DEC-C schedule. However, because PK analyses were not performed and the number of patients receiving each schedule was small, these data cannot determine whether the three-day schedule provides equivalent drug exposure or biological activity compared with the five-day schedule.

Although two partial responses were observed with DEC-C plus nivolumab, this study was not powered to formally assess efficacy, and no efficacy conclusions can be drawn from the observed ORR of 25% given the small sample size and wide confidence interval. Objective responses occurred in patients treated in earlier lines of therapy, whereas patients with prior progression on immunotherapy, who comprised the majority of our cohort, derived limited benefit. Given the small sample size and nonrandomized design, this pattern is descriptive and may reflect selection bias or differences in underlying sensitivity to PD-1 blockade. Historical studies of anti-PD-1 therapy in mucosal melanoma have reported ORRs of approximately 20–25%, refs. [4,5,9,10,11] primarily in anti-PD-1-naïve populations, including patients previously treated with anti-CTLA-4 monotherapy. Although limited by the small sample size, it is notable that among the three patients who had not progressed on prior anti-PD-1 therapy, two achieved partial responses. Therefore, these clinical observations should be interpreted as hypothesis-generating and used to inform the design of future biomarker-integrated studies, rather than to guide clinical decision-making.

Translational analyses from this study demonstrated systemic epigenetic and immune changes in circulating immune cells, including patients who received the shortened three-day DEC-C schedule, with limited descriptive analysis of matched tumor specimens. Global DNA methylation decreased in circulating immune cells across evaluable patients, consistent with the expected biological activity of HMAs. In parallel, RIG-I pathway target genes were induced in PBMCs, supporting activation of IIPS-associated programs linked to epigenetic viral mimicry. However, because methylation and gene-expression analyses were performed in circulating immune cells, these findings may not reflect epigenetic or transcriptional changes within the tumor microenvironment. Exploratory immune profiling further demonstrated a decrease in circulating CD8^+^ T cells expressing markers of exhaustive differentiation (PD-1^+^TIM-3^+^) during treatment. Among responding patients, trends toward reduced immunosuppressive populations, including Tregs and MDSCs, and increased frequencies of cytotoxic CD8^+^ T cells were observed. The reduction in Tregs included a highly immunosuppressive subset expressing PD-1 and TIM-3, ref. [38] which has been associated with poor clinical outcomes in multiple cancer types, including melanoma, bladder cancer, and non-small cell lung cancer [39,40,41]. Similarly, our group has previously shown that the MDSC frequency correlates with poor clinical responses to ICIs in patients with melanoma [29,30,42]. These findings align with prior reports of decitabine-mediated immune remodeling [14,15,16,19,20]. Finally, plasma cytokine analyses demonstrated induction of select inflammatory mediators, including IL-1β, although interferon responses were variable and not uniformly observed. Collectively, these translational findings are consistent with systemic epigenetic immune priming with DEC-C in patients with mucosal melanoma. However, the predominance of blood-based molecular analyses and limited matched tumor tissue preclude confirmation of intratumor epigenetic reprogramming or restoration of IIPS within the TME. Future studies should incorporate paired tumor and blood-based sampling to define mechanisms associated with response., while recognizing that serial biopsies in mucosal melanoma may be limited by anatomically difficult tumor sites and availability of safe biopsy targets.

This study has several important limitations. First, the trial was discontinued before completion of the phase Ib portion, and the primary endpoint of establishing the RP2D was not met. Although definitive dose selection was not possible, no unexpected safety signals were observed, and patients requiring dose modification were able to continue treatment using a shortened three-day DEC-C priming schedule. Second, this was a single-arm study without a nivolumab monotherapy control arm, limiting the ability to directly attribute clinical or translational effects to DEC-C. Third, this was a single-institution pilot study with a small sample size (*n* = 8), including only female patients, which limits generalizability and precludes formal subgroup, dose–response, or site-specific efficacy analyses. These analyses will be important in future studies, given preliminary evidence that ICI response rates may vary by mucosal site, with more favorable outcomes reported for head and neck primary tumors [43]. In addition, the study population was enriched for heavily pretreated patients, including individuals with prior exposure and progression on PD-1-based therapy, which makes cross-trial comparisons to largely anti-PD-1-naïve cohorts challenging and supports evaluation of this strategy earlier in the disease course. Finally, limited availability and quantity of paired pre- and on-treatment tumor biopsies prevented robust assessment of intratumoral epigenetic and immune effects. Only three patients had matched tumor specimens available for immune profiling, and these analyses were descriptive; therefore, this study cannot confirm restoration of innate immune signaling or epigenetic remodeling in the target tumor tissue. This also limited our ability to define the optimal timing and duration of DEC-C priming relative to nivolumab. Given potential differences in tumor cell turnover and epigenetic dynamics in vivo compared with preclinical models, future studies incorporating systematic tumor sampling will be required to optimize priming schedules and better define mechanisms of response.

## 5. Conclusions

This pilot study provides evidence that oral decitabine/cedazuridine (DEC-C) combined with nivolumab is associated with systemic epigenetic immune modulation in patients with mucosal melanoma, including global DNA hypomethylation and induction of RIG-I pathway-associated innate immune signaling in circulating immune cells. Although the recommended phase II dose (RP2D) could not be formally established due to premature trial closure, no grade 5 adverse events or unexpected safety signals were observed. Treatment-related toxicity was frequent, including grade 3–4 events, and dose modification was required for patients who experienced dose-limiting toxicities. Treatment could be continued with a shortened three-day DEC-C priming schedule when dose modification was required,. but because pharmacokinetic analyses were not performed, the optimal DEC-C dose and schedule remain undefined. Two partial responses were observed, particularly in patients treated in earlier lines of therapy; however, the small sample size, wide confidence interval, and nonrandomized design preclude conclusions regarding efficacy. Together, these findings support the design of future biomarker-integrated studies evaluating epigenetic immune-priming strategies, including IIPS activating approaches, combined with immune checkpoint blockade for patients with mucosal melanoma. These findings may also inform development of oral DNMT inhibitor-based immune-priming strategies in other melanoma subtypes and cancer types characterized by low innate immune signaling and resistance to immune checkpoint blockade.

## Figures and Tables

**Table 1:** Baseline Patient Characteristics.

Characteristic	Treated (*n* = 8)
Female	8 (100%)
Age, Median (range)	64 (34–71)
Ethnicity	
American Indian or Alaska Native	1 (12.5%)
White	6 (75%)
Hispanic or Latino	1 (12.5%)
ECOG Score	
0	5 (62.5%)
1	3 (37.5%)
Baseline Serum LDH	
Normal	1 (12.5%)
>ULN	3 (37.5%)
Unknown	4 (50%)
Disease Stage (AJCC 8th edition)	
III Unresectable	2 (25%)
IV M1b	2 (25%)
IV M1c	4 (50%)
* BRAF/NRAS/KIT*-Mutation	
*KIT*	2 (25%)
None	6 (75%)
Primary Site of Tumor	
Anorectal	4 (50%)
Vulvovaginal	3 (37.5%)
Oral	1 (12.5%)
Prior Systemic Therapy for Unresectable Stage III or Stage IV Disease	
*n* of Patients	5 (62.5%)
Median # of lines (range)	1.50 (0–4)
Prior anti-CTLA-4 monotherapy	0 (0%)
Prior anti-PD-1 monotherapy	3 (37.5%)
Prior anti-PD-1 + anti-CTLA-4	4 (50%)
Prior anti-PD-1 + anti-LAG-3	4 (50%)
Prior clinical trial	2 (25%)
Prior (Neo)Adjuvant Therapy	
*n* of Patients	2 (25%)
Neoadjuvant anti-PD-1 + anti-CTLA-4	1 (12.5%)
Adjuvant anti-PD-1	1 (12.5%)
Prior Surgery	
*n* of Patients	4 (50%)
Prior Radiation	
*n* of Patients	3 (37.5%)

**Table 2:** Treatment-Related Toxicity Profile by Study Agent.

	All		DEC-C		Nivolumab		
Adverse Event, *n* (%)	Any Grade	Grade 3–4	Any Grade	Grade 3–4	Any Grade	Grade 3–4	SAEs
Nausea	5 (63)		3 (38)		1 (13)		
Fatigue	4 (50)		4 (50)		3 (38)		
Constipation	3 (38)		1 (13)		0 (0)		
Hypoxia	3 (38)	3 (38)	0 (0)		0 (0)		1 (13)
Neutrophil count decreased	3 (38)	2 (25) ^†^	3 (38)	2 (25)	0 (0)		1 (13)
Pain	3 (38)	2 (25)	0 (0)		0 (0)		
Abdominal pain	2 (25)	2 (25)	1 (13)		0 (0)		2 (25)
Anemia	2 (25)		1 (13)		0 (0)		
Febrile neutropenia	2 (25)	2 (25) ^††^	2 (25)	2 (25)	1 (13)	1 (13)	2 (25)
Flu like symptoms	2 (25)		0 (0)		0 (0)		
White blood cell decreased	2 (25)	1 (13)	2 (25)	1 (13)	0 (0)		
Alanine aminotransferase increased	1 (13)		0 (0)		1 (13)		
Arthralgia	1 (13)		0 (0)		1 (13)		
Elevated platelet count	1 (13)		0 (0)		0 (0)		
Dizziness	1 (13)		1 (13)		0 (0)		
Dysarthria	1 (13)		0 (0)		0 (0)		
Fever	1 (13)		0 (0)		0 (0)		
Gastritis	1 (13)		0 (0)		0 (0)		
Diverticulitis	1 (13)		0 (0)		0 (0)		
Cancer-related pain	1 (13)	1 (13)	0 (0)		0 (0)		1 (13)
Generalized muscle weakness	1 (13)		1 (13)		1 (13)		
Hyperglycemia	1 (13)		0 (0)		0 (0)		
Hypokalemia	1 (13)		0 (0)		0 (0)		
Hyponatremia	1 (13)		1 (13)		1 (13)		
Infusion related reaction	1 (13)		0 (0)		1 (13)		
Lymphocyte count decreased	1 (13)		1 (13)		0 (0)		
Auditory hallucinations	1 (13)		1 (13)		0 (0)		
Rectal pain	1 (13)	1 (13)	0 (0)		0 (0)		1 (13)
Renal calculi	1 (13)		0 (0)		0 (0)		
Sinus tachycardia	1 (13)		0 (0)		0 (0)		
Lichen Sclerosis	1 (13)		0 (0)		1 (13)		
Skin infection	1 (13)		0 (0)		0 (0)		
Supraventricular tachycardia	1 (13)	1 (13)	0 (0)		0 (0)		
Urinary tract infection	1 (13)		0 (0)		0 (0)		
Vomiting	1 (13)		1 (13)		0 (0)		

^†^ Two dose-limiting toxicities, one Grade 3 and one Grade 4. ^††^ One dose-limiting toxicity, Grade 3.

## Data Availability

The original contributions presented in this study are included in the article/Supplementary Materials. Further inquiries can be directed to the corresponding authors.

## Supplementary Materials

The following supporting information can be downloaded at: https://www.mdpi.com/article/10.3390/cancers18193172/s1, Supplementary File S1: Study Protocol; Supplementary File S2: CONSORT-DEFINE checklist; Supplementary Tables (Table S1: Overview of flow cytometry panel; Table S2: Summary Statistics of Log-Transformed Values of pre- and on-treatment circulating immune cells; Table S3: Summary Statistics of Log-Transformed Values of circulating immune cells comparing responders and non-responders; Table S4: Summary Statistics of Original Scale Values of pre- and on-treatment tumor cells; Table S5: Summary Statistics of Original Scale Values of tumor cells comparing responders and non-responders; Table S6: Summary Statistics of Log-Transformed Values of pre- and on-treatment plasma cytokines; Table S7: Summary Statistics of Log-Transformed Values of plasma cytokines comparing responders and non-responders; Table S8: Representativeness of Study Participants).

## Abbreviations

The following abbreviations are used in this manuscript:

AE	Adverse event
AJCC	American Joint Committee on Cancer
CI	Confidence interval
CR	Complete response
CTCAE	Common Terminology Criteria for Adverse Events
CTLA-4	Cytotoxic T-lymphocyte–associated protein 4
DEC-C	Decitabine/cedazuridine
DLT	Dose-limiting toxicity
DNA	Deoxyribonucleic acid
DNMT1	DNA methyltransferase 1
ECOG	Eastern Cooperative Oncology Group
EOT	End of treatment
FDA	United States Food and Drug Administration
GAPDH	Glyceraldehyde-3-phosphate dehydrogenase
gDNA	Genomic DNA
GZMB	Granzyme B
HMA	Hypomethylating agent
ICI	Immune checkpoint inhibitor
IFI6	Interferon alpha-inducible protein 6
IFI27	Interferon alpha-inducible protein 27
IFI44L	Interferon-induced protein 44-like
IFN	Interferon
IIPS	Innate immune pathogen-sensing
IL	Interleukin
IQR	Interquartile range
LAG-3	Lymphocyte-activation gene 3
LDH	Lactate dehydrogenase
LINE-1	Long interspersed nuclear element-1
M:U	Methylated-to-unmethylated
MDSC	Myeloid-derived suppressor cell
MHC	Major histocompatibility complex
MTD	Maximum tolerated dose
NCCN	National Comprehensive Cancer Network
NK	Natural killer
NKG2D	Natural killer group 2 member D
NR	Not reached
ORR	Objective response rate
OS	Overall survival
PBMC	Peripheral blood mononuclear cell
PD	Progressive disease
PD-1	Programmed death-1
PFS	Progression-free survival
PR	Partial response
qRT-PCR	Quantitative real-time polymerase chain reaction
RECIST	Response Evaluation Criteria in Solid Tumors
RIG-I	Retinoic acid-inducible gene-I
RNA	Ribonucleic acid
RP2D	Recommended phase II dose
SAE	Serious adverse event
SD	Stable disease
SEM	Standard error of the mean
TIM-3	T-cell immunoglobulin and mucin-domain containing-3
TITE-BOIN	Time-to-event Bayesian optimal interval
TNF-α	Tumor necrosis factor-alpha
Treg	Regulatory T cell
ULN	Upper limit of normal
