# Supplementary material for: Epigenetic Priming with Oral Decitabine/Cedazuridine Combined with Nivolumab in Unresectable or Metastatic Mucosal Melanoma: Clinical and Correlative Findings from a Phase Ib/II Trial
Source: Cancers (Basel). 2026 Oct 1;18(19):3172. doi: 10.3390/cancers18193172 (PMC13664269; doi:10.3390/cancers18193172)
Supplement: Supplementary file 1 [file cancers-18-03172-s001.zip › Supplementary File S1_09.30.pdf]

Oral Decitabine/Cedazuridine (DEC-C) in Combination with Nivolumab as a Strategy to Enhance the Efficacy of Immune Checkpoint Blockade in Unresectable or Metastatic Mucosal Melanoma

**Protocol Number:**	21-3518
**National Clinical Trial (NCT) Number:**	NCT05089370
**Principal Investigator:**	Martin McCarter, MD<br>Department of Surgery<br>Mail Stop C313<br>12631 East 17th Avenue<br>Aurora, CO 80045<br><br>P – 303-724-2725<br>F – 303-724-2733
**IND/IDE Sponsor:**	Martin McCarter, MD
**Funded by:**	National Comprehensive Cancer Network (NCCN) is funding through a grant from Taiho Oncology, Inc. (2021-IST-DECC-000076)

PROTOCOL AMENDMENT HISTORY

Version	Date	Description of Change	Brief Rationale
v05.20.2021	05.20.2021	N/A	N/A
v04.15.2022	04.15.2022	Clarification of "Taiho" to "Taiho Oncology, Inc." & alignment of DSMC language	Requirements to satisfy NCCN/Taiho Oncology, Inc. with our contract & support our current institutional language
v06.28.2022	06.28.2022	Clarification of the Statistical Analyses in Section 9.4<br>Addition of treated and stable thyroid disease to exclusion criterion #1 in Section 5.2<br>Changed iRECIST to RECIST	Changed the design to a TITE-BOIN with a cohort size of 3 instead of 1 to potentially accelerate the trial by allowing new patients to enroll while there are patients with pending DLTs.<br>Treated and stable thyroid disease was added an exception as the melanoma is generally a life-limiting cancer. If the potential participant is not on active therapy for the thyroid cancer, then they could be considered.<br>We changed from iRECIST to RECIST as this is the primary criteria used for the Nivolumab programs.

TABLE OF CONTENTS

STATEMENT OF COMPLIANCE 6
LIST OF ABBREVIATIONS 7
PARTICIPATING Sites .... Error! Bookmark not defined.
1 PROTOCOL SUMMARY 10
1.1 Synopsis 10
1.2 Study Schema 11
1.3 Schedule of Events 12
2 INTRODUCTION 13
2.1 Study Rationale 13
2.2 Background 13
2.2.1 Pharmaceutical and Therapeutic Background 14
2.2.2 Preclinical and Clinical Trial Data 15
2.3 Risk/Benefit Assessment 15
2.3.1 Known Potential Risks 15
2.3.2 Known Potential Benefits 16
2.3.3 Assessment of Potential Benefits 16
3 OBECTIVES AND ENDPOINTS 17
4 STUDY DESIGN 17
4.1 Overall Design 17
4.2 scientific RAtionale for STudy design 18
4.3 Justification for Dose 18
4.4 End of Study Definition 19
5 STUDY POPULATION 19
5.1 Inclusion Criteria 19
5.2 Exclusion Criteria 20
5.3 Lifestyle Considerations 21
5.3.1 Diet 21
5.3.2 Contraception 21
5.3.3 Use in Pregnancy 23
5.3.4 Use in Nursing Women 23
5.4 Screen Failures 23
5.5 Strategies for Recruitment and Retention 23
6 STUDY INTERVENTION 24
6.1 Study Intervention(s) Administration 24
6.1.1 Study Intervention Description 24
6.1.2 Dosing and Administration 24
6.2 Preparation/Handling/Storage/Accountability 31
6.2.1 Acquisition and Accountability 31
6.2.2 Formulation, Appearance, Packaging, and Labeling 32
6.2.3 Product Storage and Stability 32
6.2.4 Preparation 32
6.3 Measures to Minimize Bias: Randomization and Blinding 33
6.4 Study Intervention Compliance 33
6.5 Prohibited Therapy 33

6.6 Concomitant Therapy 34
6.6.1 Rescue Medicine 34
7 STUDY INTERVENTION DISCONTINUATION AND PARTICIPANT DISCONTINUATION/WITHDRAWAL 34
7.1 Discontinuation of Study Intervention (Stopping Rules) 34
7.1.1 Discontinuation of Study Therapy after CR 34
7.1.2 Discontinuation of Study Therapy after Disease Stability 35
7.1.3 Toxicity and Study Discontinuation Criteria 35
7.2 Participant Discontinuation/Withdrawal from Study 35
7.3 Lost to Follow-Up 36
8 STUDY ASSESSMENTS AND PROCEDURES 36
8.1 Efficacy Assessments 36
8.2 Safety and Other Assessments 36
8.3 Study Procedures 37
8.3.1 Administrative Procedures 37
8.3.2 Clinical Assessments 38
8.3.3 Tumor Tissue Collection and Correlative Studies Blood Sampling 39
8.3.4 Laboratory Procedures/Assessments 40
8.3.5 Visit Requirements 41
8.4 Adverse Events and Serious Adverse Events 42
8.4.1 Definition of Adverse Events (AE) 42
8.4.2 Definition of Serious Adverse Events (SAE) 42
8.4.3 Classification of an Adverse Event 42
8.4.4 Time Period and Frequency for Event Assessment and Follow-Up 44
8.4.5 Adverse Event Reporting 45
8.4.6 Serious Adverse Event Reporting 45
8.4.7 Reporting of Pregnancy 49
8.5 Unanticipated Problems 49
8.5.1 Definition of Unanticipated Problems (UAP) 49
8.5.2 Reporting of Unanticipated Problems 49
9 STATISTICAL CONSIDERATIONS 50
9.1 Statistical Hypotheses 50
9.2 Sample Size Determination 50
9.3 Population for Analyses 50
9.4 Statistical Analyses 51
9.4.1 General Approach 51
9.4.2 Analysis of the Primary Efficacy Endpoint(s) 54
9.4.3 Analysis of the Secondary Endpoint(s) 54
9.4.4 Safety Analyses 54
9.4.5 Baseline Descriptive Statistics 55
9.4.6 Planned Interim Analyses 55
9.4.7 Tabulation of Individual Participant Data 55
9.4.8 Exploratory Analyses 55
10 SUPPORTING DOCUMENTATION AND OPERATIONAL CONSIDERATIONS 56
10.1 Regulatory, Ethical, and Study Oversight Considerations 56
10.1.1 Informed Consent Process 56

10.1.2 Study Discontinuation and Closure 57
10.1.3 Confidentiality and Privacy 57
10.1.4 Future Use of Stored Specimens or Data 58
10.1.5 Safety Oversight 59
10.1.6 Clinical Monitoring 60
10.1.7 Quality Assurance and Quality Control 60
10.1.8 Data Handling and Record Keeping 61
10.1.9 Protocol Deviations 61
10.1.10 Publication and Data Sharing Policy 62
10.1.11 Conflict of Interest Policy 62
11 REFERENCES 63

STATEMENT OF COMPLIANCE

This is an investigator-initiated study. The Principal Investigator (PI), Martin McCarter, MD, is conducting the study and acting as the sponsor. As the Sponsor-Investigator, both the legal/ethical obligations of a PI and those of a sponsor will be followed.

The trial will be carried out in accordance with Good Clinical Practice (GCP) as required by applicable United States (US) laws and applications, including but not limited to United States (US) Code of Federal Regulations (CFR) applicable to clinical studies (45 CFR Part 46, 21 CFR Part 50, 21 CFR Part 56, 21 CFR Part 312, and/or 21 CFR Part 812), as applicable.

The PI will assure that no changes to the protocol will take place without documented approval from the Institutional Review Board (IRB). All personnel involved in the conduct of this study have completed Human Subjects Protection Training.

I agree to ensure that all staff members involved in the conduct of this study are informed about their obligations in meeting the above commitments.

Sponsor-Principal Investigator: Martin McCarter, MD
Print/Type Name

Signature: ______________________________ **Date:** __________

LIST OF ABBREVIATIONS

ACRONYM	DESCRIPTION
ADL	Activities of daily living
AE	Adverse event
AR	Adverse reaction
ALT	Alanine aminotransferase
ANC	Absolute neutrophil count
aPTT	Activated partial thromboplastin time
AST	Aspartate aminotransferase
BCG	Bacillus Calmette-Guerin
BMS	Bristol Myers Squibb
CBC	Complete blood count
CFR	Code of federal regulations
CM	Cutaneous melanoma
CNS	Central nervous system
COMIRB	Colorado multiple institutional review board
CR	Complete response
CrCl	Creatinine clearance
CRF	Case report form
CT	Computed tomography
CTCAE	Common Terminology Criteria for Adverse Events
CU	University of Colorado
DISC	University of Colorado-Denver Development and Informatics Service Center
DKA	Diabetic ketoacidosis
dL	Deciliter
DLT	Dose limiting toxicity
DNMT1	DNA methyltransferase 1
DSM	Data and safety monitoring
DSMC	Data and safety monitoring committee
ECI	Event of clinical interest
ECOG	Eastern cooperative oncology group
EOT	End of treatment
ERC	Ethics resource center
FDA	Food and drug administration
FDAAA	Food and Drug Administration amendments act
FDAMA	Food and Drug Administration Modernization Act
FSH	Follicle-stimulating hormone
g	Gram
GCP	Good clinical practice
GFR	Glomerular filtration rate
GI	Gastrointestinal
GM-CSF	Granulocyte-macrophage colony stimulating factor

HCG	Human chorionic gonadotropin
HIV	Human immunodeficiency virus
HLA	Human leukocyte antigen
HMA	Hypomethylating agent
ICH	International conference on harmonization
IMAR	Immune-mediated adverse reaction
INR	International normalized ratio
IRB	Institutional review board
IUD	Intrauterine device
IV	Intravenous
kg	Kilogram
L	Liter
m	Meter
mcL	Microliter
MDSC	Myeloid-derived suppressor cell
mg	Milligram
mL	Milliliter
MM	Mucosal melanoma
MRI	Magnetic resonance imaging
MTD	Maximum tolerated dose
NCCN	National comprehensive cancer network
NCI	National Cancer Institute
NSAID	Non-steroidal anti-inflammatory drug
NSCLC	Non-small cell lung cancer
ORR	Overall response rate
OS	Overall survival
OTC	Over the counter
PBMC	Peripheral blood mononuclear cell
PFS	Progression-free survival
PCR	Polymerase chain reaction
PI	Principal Investigator
PK	Pharmacokinetics
PO	By mouth
PT	Prothrombin time
Q4W	Every four weeks
RP2D	Recommended Phase 2 dose
SAE	Serious adverse event
SGOT	Serum glutamic oxaloacetic transaminase
SGPT	Serum glutamic pyruvic transaminase
T1DM	Type 1 diabetes mellitus
TB	Tuberculosis
TSH	Thyroid-stimulating hormone
UAP	Unanticipated problem
UCCC	University of Colorado Cancer Center

ULN	Upper limit of normal
US	United States

PARTICIPATING SITES

University of Colorado Cancer Center
Anschutz Cancer Pavilion
1665 Aurora Court
Aurora, CO 80045

1 PROTOCOL SUMMARY

1.1 SYNOPSIS

**Protocol Title:**	Oral Decitabine/Cedazuridine (DEC-C) in Combination with Nivolumab as a Strategy to Enhance the Efficacy of Immune Checkpoint Blockade in Unresectable or Metastatic Mucosal Melanoma
**Objectives:**	• ***Primary Objective:***<br>• Establish the safety and tolerability of DEC-C in combination with Nivolumab in unresectable, locally advanced, or metastatic mucosal melanoma patients.<br>• ***Secondary Objectives:***<br>• Determine the response rate to DEC-C in combination with Nivolumab in unresectable, locally advanced, or metastatic mucosal melanoma patients.<br>• Determine if the addition of DEC-C to Nivolumab increases progression free survival (PFS) and overall survival (OS) in unresectable, locally advanced, or metastatic mucosal melanoma patients.<br>• ***Tertiary/Exploratory Objectives:***<br>• Evaluate the effect of DEC-C in combination with Nivolumab on circulating and tumor immune cell profiles.<br>• Determine the effect of DEC-C in combination with Nivolumab on global hypomethylation and RIG-I pathway gene expression in circulating cells and tumor cells.<br>• Determine the effect of DEC-C in combination with Nivolumab on circulating and tumor innate immune sensing cytokines and proteins.
**Endpoint:**	• ***Primary Endpoint:***<br>• Determine the safety and tolerability of DEC-C in combination with Nivolumab by evaluating adverse events (AEs) using CTCAEv5.0 criteria and establishing the recommended phase 2 dose (RP2D) of DEC-C.

- ***Secondary Endpoints:***
  - Evaluate the rate of subjects that achieve an objective response (ORR) to DEC-C in combination with Nivolumab by radiographic assessment using RECIST criteria.
  - Evaluate and estimate progression free survival (PFS) and overall survival (OS) in unresectable locally advanced and metastatic mucosal melanoma patients at 24 months post-study.
- ***Tertiary/ exploratory:***
  - Compare the frequency and activity of circulating and tumor immune cells pre- and on- treatment by flow cytometry.
  - Evaluate pre- and on- treatment levels of global hypomethylation and RIG-I pathway gene expression in circulating and tumor cells by qRT-PCR.
  - Analyze pre- and on- treatment levels of circulating and tumor immune cytokines and proteins by Olink proteomics analysis.

Population:

- ***Sample size***
  - Maximum number of participants that can be enrolled is 30 (allow for screen failures and non-evaluable patients)
  - Minimum number of participants to be enrolled 9-19 (answer scientific question/aims)

Phase: Ib/II

Participating Sites: University of Colorado Cancer Center

Description of Study Intervention: DEC-C and Nivolumab

Study Duration: 3 years

Participant Duration: 3 years

1.2 STUDY SCHEMA

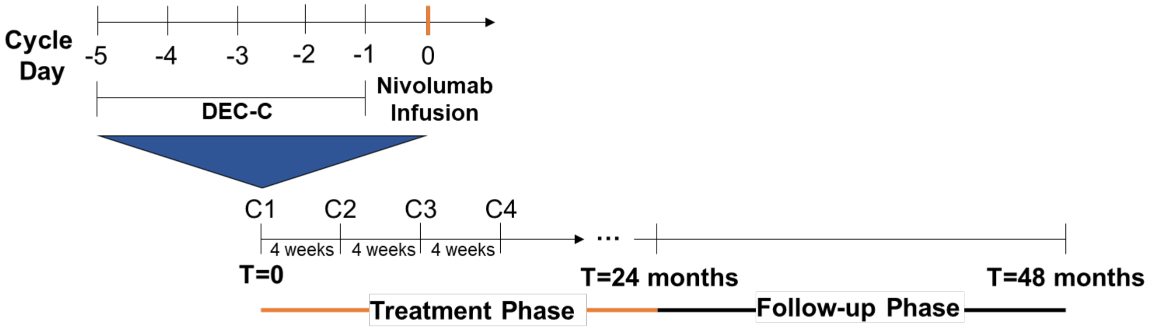

Figure 1. Study Schema. DEC-C will be started 5 days prior to Nivolumab infusion on a 28-day cycle. The 24-month treatment phase will be followed by a 24-month follow-up phase.

1.3 SCHEDULE OF EVENTS

Table 1. Schedule of Events

**Trial Period:**	**Screening Phase**	**Treatment Cycles**					**Post-Treatment**			
Treatment Cycle/Title:	Screening	1	2	3	4	5+	EOT[i]	Safety Follow-up 1	Safety Follow-up 2[b]	Survival Follow-up
Scheduling Window (Days):	-28 to -5		± 7	±7	± 7	± 7	± 7	30 days (±2 days) post last treatment or prior to starting new treatment	90 days (±7 days) post last treatment	Every 12 weeks (±14 days) from EOT
**Administrative Procedures**										
Informed Consent	x									
Inclusion/Exclusion Criteria	x									
Demographics and Medical History	x									
Prior and Concomitant Medication Review	x	x	x	x	x	x	x	x	x	x
DEC-C Administration[a]		x	x	x	x	x				
Nivolumab Administration		x	x	x	x	x				
Post-study anticancer therapy status							x	x	x	x
Survival Status								x	x	x
**Clinical Procedures/Assessments**										
Review Adverse Events		x	x	x	x	x	x	x	x	
Full Physical Examination	x									
Directed Physical Examination		x	x	x	x	x		x		
Vital Signs and Weight	x							x		
ECOG Performance Status	x	x	x	x	x	x	x			
**Laboratory Procedures/Assessments: analysis performed by LOCAL laboratory**										
Pregnancy Test – Urine or Serum β-HCG[h]	x									
CBC with Differential[g]	x	x	x	x	x	x				
Comprehensive Serum Chemistry Panel[e & g]	x	x	x	x	x	x				
Total T3, FT4 and TSH[g]	x	x		x		x[f]				
**Efficacy Measurements**										
Tumor Imaging[c]	x			x		x	x			
**Tumor Biopsies Collection/Correlative Studies Blood**										
Newly Obtained Tissue Collection[d]	x		x							
Archival Tissue Collection[j]	x									
Correlative Studies Blood Collection	x		x		x		x			

[a] Administered according to schedule outlined in section 6.1. DEC-C drug supply will be dispensed in clinic on Cycle 1 Day -5 for Cycle 1, Cycle 1 Day 0 for Cycle 2, Cycle 2 Day 0 for Cycle 3 and Cycle 3 Day 0 for Cycle 4, etc.
[b] To be completed only for subjects who have not initiated a new therapy. Can be collected at a regularly scheduled clinic visit or via patient contact by telephone.
[c] Tumor imaging generally performed every 12 weeks per standard of care for the clinic and evaluated by RECIST. Baseline tumor imaging should be completed within 28 days of starting treatment; however, exceptions may be approved after discussion with the principal investigator. Brain metastases that were radiated prior to starting the trial should be re-imaged at the standard 12-week frequency, or as deemed clinically appropriate, by the treating physician.
[d] Patients will have biopsies performed prior to treatment and around cycle 2 unless the tumor is inaccessible, the biopsy is not in the subject's best interest, or the patient refuses biopsy during course of the study.
[e] To include lactate dehydrogenase at screening.
[f] To be collected every other cycle per standard of care for clinic.
[g] Screening labs should be performed within 10 days of DEC-C treatment.
[h] Within 72 hours prior to receiving the first dose of DEC-C.
[i] To be completed when it is decided that the subject will discontinue study treatment. Tumor imaging is only required at investigator's discretion. This visit can be combined with the first safety follow-up visit if the subject is planning to start a new treatment.
[j] Archival tissue may be requested from previously collected biopsies for analysis.

2 INTRODUCTION

2.1 STUDY RATIONALE

Activation of the RIG-I innate immune pathway and increased expression of tumor antigens and pro-immune genes by DEC-C during Nivolumab treatment may enhance the frequency and activity of anti-tumor immune cells (CD4+ and CD8+ T-cells, NK cells) and reduce the frequency and activity of immunosuppressive cells (MDSCs, Tregs) (*1-4*). This may increase the overall effectiveness and success of Nivolumab treatment. This pilot clinical trial will demonstrate whether combinatorial immunotherapeutic approaches that target epigenetic immune repression and RIG-I activity can favorably alter the tumor immune cell microenvironment and benefit patients with mucosal melanoma.

2.2 BACKGROUND

Malignant melanoma typically develops on the skin (cutaneous melanoma, CM), but it can also arise in other locations such as in the mucosa (mucosal melanoma, MM) (*5*). MM accounts for 1.3% of all malignant melanomas and develops in the head and neck (nasal, sinus, and oral) and genitourinary (vaginal, penile, and anorectal) regions (*6*). The 5-year survival rate for MM (25%) is significantly worse than CM (81%) (*5*). This is largely because MM is detected at later stages, is challenging to surgically resect, and is resistant to standard melanoma therapies including immunotherapy (*7-10*).

We and others find immune checkpoint blockade therapy (ICB, including anti-CTLA4, anti-PD1, and the combination) is less effective in MM compared to CM (*8, 9*). The current standard of care for patients with metastatic melanoma, including MM, is combination anti-CTLA4/anti-PD1 immunotherapy. CM patients have an overall response rate (ORR) of 60% to this combination (*11*), compared to only 32% for mucosal melanoma patients (*8, 12*). Unfortunately, this combination is often limited by severe immune-related adverse events (*11*). Single agent anti-PD1 immunotherapies, such as Nivolumab, are effective treatments for patients with metastatic melanoma and are less toxic. CM has an ORR of 40-50% to single-agent anti-PD1 (*13*), but the average ORR is only 22% for MM (*9*). Additionally, significantly fewer patients with MM achieve complete response compared to patients with CM (*14*).

We discovered that the RIG-I innate immune pathogen sensing pathway is inhibited in MM compared to CM. In CM, the RIG-I pathway is critical for response to anti-PD1 because it enhances antigen presentation and promotes a favorable immune cell profile in the tumor environment (*15-17*). High RIG-I pathway activity in CM leads to elevated expression of MHC Class I and II, increased frequency and activity of CD4+ and CD8+ T-cells, and decreased number and activity of immune-suppressive myeloid-derived suppressor cells (MDSCs) and T-regulatory cells (Tregs) (*17*). Direct RIG-I agonists are being developed, but they are not available in the clinic yet. Interestingly, hypomethylating agents (HMAs) inhibiting DNA methyltransferase 1 (DNMT1), specifically Decitabine, activates innate immune sensing by inducing global hypomethylation, which increases innate immune sensing genes, endogenous retroviruses, tumor antigens, and NK cell ligands (*3, 18*).

Altogether, these data suggest that low RIG-I pathway gene expression in MM is a potential mechanism underlying low anti-tumor immunity and innate resistance to anti-PD1. Further, re-activation of the RIG-I pathway using decitabine in combination with anti-PD1 therapy may be a novel strategy for sensitizing MM tumors to anti-PD1.

2.2.1 PHARMACEUTICAL AND THERAPEUTIC BACKGROUND

Decitabine is a cytidine-nucleoside analogue that incorporates into DNA during the S-phase of the cell cycle. It covalently binds DNA methyltransferase 1 (DNMT1) and leads to its degradation, which results in reduced methylation of CpG residues in genomic DNA, altered epigenetic patterns, and modified gene expression (*19*). Decitabine is a standard treatment currently approved for patients with hematological cancers including myelodysplastic syndrome (MDS) and chronic myelomonocytic leukemia (CMML).

Decitabine is rapidly deaminated by cytidine deaminases *in vivo*, making it difficult to achieve stable pharmacokinetics and limiting dosing routes to intravenous or subcutaneous injections because of the high level of cytidine deaminases in the gastrointestinal (GI) tract (*20*). The lack of an oral form of dosing for Decitabine was particularly inconvenient since standard Decitabine dosing is 5 consecutive days per 28-day cycle, for 4 or more cycles. To overcome this limitation, Astex Pharmaceuticals, Inc. developed an oral formulation (ASTX727) of Decitabine combined with Cedazuridine, a cytidine deaminase inhibitor which is stable in acidic environments (*21*).

DEC-C (trade name "INQOVI") is an FDA-approved, commercially available oral formulation of 35mg Decitabine/100 mg Cedazuridine tablets, and the recommended dose is one tablet per day, for 5 days, per 28-day cycle. It is administered for 4 consecutive cycles, but continued dosing may be needed to achieve complete or partial response (*19*). In July 2020, DEC-C received approval for the treatment of MDS and CMML in both the United States and Canada. DEC-C in combination with Nivolumab is investigational and has not been approved.

2.2.2 PRECLINICAL AND CLINICAL TRIAL DATA

Pre-clinical data shows Decitabine favorably alters the melanoma tumor immune microenvironment (*22*), and early clinical trials show Decitabine has immune modulatory function in melanoma patients (*23, 24*). An initial study combining oral 5'-azacytidine with anti-PD1 showed minimal toxicity compared to single-agent anti-PD1, but had disappointing efficacy results, which was likely due to low drug effects on methylation and a lack of RIG-I activation by 5'-azacytidine (*25*).

Our preliminary research using transcriptomic data for MM and CM tumors show the RIG-I pathway is inhibited in MM compared to CM. Consistent with this, we find MM cell lines and patient tumors have low immunogenicity. MM cells have significantly lower surface expression of antigen and immunogenicity proteins MHC Class I, MHC Class II, and PD-L1 compared to ICB- responsive CM cells. MM tumors also have fewer CD8+ and CD4+ lymphocytes in both the tumor and surrounding stroma compared to CM.

We treated ICB-resistant MM cells with low/high doses of two HMAs, 5'-azacytidine and decitabine. Consistent with previous studies (*3*), we found Decitabine is a more potent inducer of innate immune sensing genes. Further, Decitabine also induced expression of NK cell ligands and tumor antigens, as well as increasing cell surface expression of MHC Class I and II.

2.3 RISK/BENEFIT ASSESSMENT

2.3.1 KNOWN POTENTIAL RISKS

2.3.1.1 NIVOLUMAB SAFETY PROFILE

Nivolumab is generally well tolerated and demonstrates a favorable safety profile in comparison to chemotherapy. Nivolumab is an immunomodulatory agent, and based on this mechanism of action, immune-mediated adverse reactions (IMARs) are of primary concern. An IMAR is defined as a clinically significant AE of any organ that is associated with study drug exposure, is of unknown etiology, and is mediated by the immune response. If an IMAR is suspected, a thorough evaluation should be conducted in an effort to possibly rule out neoplastic, infectious, metabolic, toxin, or other etiologic causes before diagnosing an IMAR. Serological, immunological, and histological (biopsy) data should be considered to support the diagnosis of an immune-mediated toxicity.

Immune-mediated adverse reactions (AR), including severe and fatal cases, have occurred in patients receiving Nivolumab. Immune-mediated ARs can occur after discontinuation of treatment. Immune-mediated ARs affecting more than one body system can occur simultaneously. In clinical studies, most immune-mediated ARs were reversible and managed with interruptions of Nivolumab, administration of corticosteroids, and/or supportive care.

2.3.1.2 DEC-C SAFETY PROFILE

Although DEC-C is a standard treatment for patients with MDS and CMML, it is being investigated in patients with solid tumors (*26*). Virtually all patients experience some drug-related toxicity, especially myelosuppression. These adverse effects are seldom permanent or irreversible. Myelosuppression is the most common cause of dose interruption and/or reduction (36%) but rarely leads to drug discontinuation (1%).

2.3.2 KNOWN POTENTIAL BENEFITS

Currently, there are few effective treatments, and no clinical trials developed specifically to benefit, patients with mucosal melanoma. Mucosal melanomas have low immunogenicity and activity of the RIG-I pathway, which are both known to be reversed by Decitabine in melanomas *in vitro* and *in vivo*. Therefore, DEC-C has the potential to epigenetically prime immunologically quiescent tumors for improved anti-tumor immunity, enhanced response to Nivolumab, and increased overall survival for patients.

2.3.3 ASSESSMENT OF POTENTIAL BENEFITS

The risks to participants are reasonable in relation to the anticipated benefits to participants and/or society, and in relation to the importance of the knowledge that may reasonably be expected to result, thereby falling in favor of performing the study:

- To Participant: The potential benefit to the study subject is increased anti-tumor immunity and enhanced immunologic responses.
- To Society: The potential benefit to society is generalizable knowledge regarding utilization of combinational immunotherapies.
- Importance of the knowledge gained: The knowledge to be gained includes a better understanding of anti-tumor immunity and tumor induced immunosuppression mechanisms mediated by epigenetic silencing and the RIG-I pathway.

3 OBJECTIVES AND ENDPOINTS

OBJECTIVES	ENDPOINTS	JUSTIFICATION FOR ENDPOINTS
**Primary**		
• Establish the safety and tolerability of DEC-C with Nivolumab in unresectable, locally advanced, or metastatic mucosal melanoma patients.	• Determine the safety and tolerability of DEC-C in combination with Nivolumab by evaluating adverse events (AEs) using CTCAEv5.0 criteria and establishing the recommended phase 2 dose (RP2D) of DEC-C.	• This is the first time this combination is being used in mucosal melanoma patients; therefore, safety needs to be established before evaluating efficacy.
**Secondary**		
• Determine the response rate to DEC-C in combination with Nivolumab in unresectable, locally advanced, or metastatic mucosal melanoma patients.	• Evaluate the rate of subjects that achieve an objective response (ORR) to DEC-C in combination with Nivolumab by radiographic assessment using RECIST criteria.	• This endpoint will determine if combination is more effective than current standard of care.
• Determine if the addition of DEC-C to Nivolumab increases progression free survival (PFS) and overall survival (OS) in unresectable, locally advanced, or metastatic mucosal melanoma patients.	• Evaluate and estimate progression free survival (PFS) and overall survival (OS) in unresectable locally advanced and metastatic mucosal melanoma patients at 24 months post-study.	• This endpoint will determine if combination is more effective than current standard of care.
**Tertiary/Exploratory**		
• Evaluate the effect of DEC-C in combination with Nivolumab on circulating and tumor immune cell profiles.	• Compare the frequency and activity of circulating and tumor immune cells pre- and on-treatment by flow cytometry.	• This endpoint will determine if the intended immunological targets of the combination are being affected.
• Determine the effect of DEC-C in combination with Nivolumab on global hypomethylation and RIG-I pathway gene expression in circulating cells and tumor cells.	• Evaluate pre- and on- treatment levels of global hypomethylation and RIG-I pathway gene expression in circulating and tumor cells by qRT-PCR.	• This endpoint will determine if the intended methylation and RIG-I targets of the combination are being affected.
• Determine the effect of DEC-C in combination with Nivolumab on circulating and tumor innate immune sensing cytokines and proteins.	• Analyze pre- and on- treatment levels of circulating and tumor immune cytokines and proteins by Olink proteomics analysis.	• This endpoint will determine if the intended immunological targets of the combination are being affected.

4 STUDY DESIGN

4.1 OVERALL DESIGN

This is a Phase Ib/II investigator-initiated open label trial of the combination of DEC-C and Nivolumab treatment.

This is a single-center study to be conducted at the University of Colorado Cancer Center (UCHealth central campus). A complete and current listing of investigators, research personnel, and research facilities participating in this study will be maintained throughout the duration of this study on applicable study required forms such as an *FDA Form 1572*, the *COMIRB Research Personnel Form*, and/or a *UCCC Protocol Contact List*, incorporated herein by reference.

Phase Ib: A time to event Bayesian optimal interval (TITE-BOIN) design will be used to assess the RP2D during phase 1b (*27*). The BOIN design is a model assistant approach used in phase I clinical trial for finding the maximum tolerated dose (MTD). We anticipate a maximum of 9 patients treated at the RP2D in Phase 1b dose de-escalation and we consider as subjects in stage 1 of phase 2.

Phase II: The objective of Phase II is to preliminarily evaluate the efficacy and further evaluate safety of the combination at the RP2D. The phase II cohort will enroll patients using the established RP2D of DEC-C from the phase Ib portion in combination with standard doses of Nivolumab. Patients treated at the RP2D in Phase 1b will be included in analysis for the Phase II component of this study. All patients enrolled at the RP2D level and receiving any DEC-C treatment will be included in the efficacy and safety/tolerability endpoint analysis. Our results indicate that the objective response rate (ORR) to anti-PD1 in mucosal melanoma patients at our institution is 20%. We hypothesize ORR for the combination will be 43%, a 23% absolute improvement over the anti-PD1 treatment. Simon's 2-stage design will be used to evaluate the ORRs (*28*). The null hypothesis that the ORR is 20% will be rejected if there are 3 or more responses among the 11 patients (including phase Ib RP2D patients). The design yields a type I error rate of 0.1 and power of 80% when the true response rate is 43%. The total sample size required for the Phase II will depend on the number of patients treated at the RP2D during Phase 1b, and we estimated the total sample size will be in the range of 19-21 subjects.

4.2 SCIENTIFIC RATIONALE FOR STUDY DESIGN

Activation of the RIG-I innate immune pathway and increased expression of tumor antigens and pro-immune genes during Nivolumab treatment may enhance the frequency and activity of anti-tumor immune cells (CD4+ and CD8+ T-cells, NK cells) and reduce the frequency and activity of immunosuppressive cells (MDSCs, Tregs) (*1-4*), increasing the overall effectiveness and success of Nivolumab treatment. This pilot clinical trial will demonstrate whether combinatorial immunotherapeutic approaches that target epigenetic immune repression and RIG-I activity can favorably alter the tumor immune cell microenvironment and benefit mucosal melanoma patients.

4.3 JUSTIFICATION FOR DOSE

DEC-C: Although DEC-C is a standard treatment for patients with MDS and CMML, it is being investigated in patients with solid tumors (*26*). We have selected the dose of DEC-C based on its standard recommended dosing, the available oral formulation, and other trials which have investigated reduced Decitabine dosing o combined oral Decitabine with anti-PD1. We have selected the standard dose of DEC-C based on the limited additional toxicities observed in

combining Decitabine, or related HMAs 5'-azacytidine and Guadecitabine, with immunotherapies, including anti-PD1 (*24, 29*).

Nivolumab: Nivolumab monotherapy has been extensively studied in multiple tumor types, including melanoma, NSCLC, RCC, cHL, squamous cell carcinoma of the head and neck (SCCHN), and urothelial carcinoma, using body weight normalized dosing (mg/kg), and has been safely administered at doses up to 10 mg/kg Q2W. Nivolumab is currently approved for the treatment of various tumors, including melanoma, NSCLC, RCC, cHL, SCCHN, and urothelial carcinoma, using a regimen of either nivolumab 240 mg Q2W, nivolumab 3 mg/kg Q2W, and nivolumab 480 mg Q4W. We have selected nivolumab 480 mg Q4W to minimize patient visits and align with dosing immediately after DEC-C administration.

4.4 END OF STUDY DEFINITION

The start of the trial is defined as first visit for first participant screened. End of trial is defined as the last visit or scheduled procedure shown in the Schedule of Activities for the last participant. Study completion is defined as the final date on which data for the primary endpoint was or is expected to be collected if this is not the same.

5 STUDY POPULATION

5.1 INCLUSION CRITERIA

In order to be eligible to participate in this study, an individual must meet all of the following criteria:

1. Provision to sign and date the consent form.
2. Stated willingness to comply with all study procedures and be available for the duration of the study.
3. Be a male or female aged 18-100.
4. Histologically confirmed diagnosis of advanced mucosal melanoma (unresectable Stage III or Stage IV Melanoma). Anatomical locations for primary site of mucosal melanoma include oral cavity (excluding lip), nasopharynx, vagina/vulva, and rectum.
5. Prior immune checkpoint blockade therapy, including anti-PD1 and anti-CTLA4 for unresectable locally advanced or metastatic disease, is allowed. The combination of anti-PD1 and anti-CTLA4 is also allowed as a prior line of therapy. Study therapy must not be initiated within 30 days of previous immune checkpoint blockade therapy (excluding adjuvant anti-PD1 and anti-CTLA4).
6. Patients must have systemic cross-sectional imaging (PET/CT or CT of chest, abdomen, and pelvis) with radiographically measurable, by immune-RECIST (RECIST) criteria, or clinically measurable disease.
7. Previously treated brain metastatic disease is allowed. Stability of brain metastases must be confirmed by MRI > 4 weeks from most recent treatment and within 4 weeks of initiating study therapy.
8. Patients must have an ECOG performance status of 0 or 1 (**Table 5**).

9. Patients must have adequate bone marrow function as evidenced by all of the following: ANC ≥ 2,500 microliter (mcL); platelets ≥ 100,000/mcL; Hemoglobin ≥ 10 g/dL.
10. Patients must have adequate hepatic function as evidenced by the following: total bilirubin ≤ 1.5 x institutional upper limit of normal (IULN) (except Gilbert's Syndrome, who must have a total bilirubin < 3.0 mg/dL), and SGOT (AST) and SGPT (ALT) and alkaline phosphatase ≤ 2.5 x ULN.
11. Patients must have adequate renal function as evidenced by ONE of the following: serum creatinine ≤ 2.0 x ULN OR measured or calculated creatinine clearance ≥ 40 mL/min.
12. Patients known to be HIV positive are eligible if they meet the following criteria within 30 days prior to registration: stable and adequate CD4 counts (≥ 350 mm3), and serum HIV viral load of < 25,000 IU/ml. Patients may be on or off anti-viral therapy so long as they meet the CD4 count criteria.
13. Women of childbearing potential must have a negative urine or serum pregnancy test within 72 hours prior to receiving the first dose of study medication. Women/men of reproductive potential must have agreed to use an effective contraceptive method for the course of the study through 120 days after the last dose of study medication. Should a woman become pregnant or suspect she is pregnant while she or her partner is participating in this study, she should inform her treating physician immediately. A woman is considered to be of "reproductive potential" as defined in section 5.3.2.
14. Patients must be willing to have archived tumor specimens utilized for correlative studies if available.
15. Patients must consent to have biopsies performed prior to treatment and around cycle 2 during study. However, in the event that tumor is inaccessible, the biopsy is not in the subject's best interest, or the patient refuses biopsy during course of the study, patients will be allowed to remain on study.

5.2 EXCLUSION CRITERIA

An individual who meets any of the following criteria will be excluded from participation in this study:

1. No other prior malignancy is allowed except for the following: adequately treated basal cell or squamous cell skin cancer, in situ cervical cancer, lobular carcinoma of the breast in situ, atypical melanocytic hyperplasia or melanoma in situ, treated and stable thyroid cancer, adequately treated Stage I cancer (including multiple primary melanomas) from which the patient is currently in complete remission, or any other cancer from which the patient has been disease free for three years.
2. Patients must not currently be on other drugs metabolized by CDA.
3. Prior cytotoxic chemotherapy treatment received within 30 days of study enrollment.
4. Patients with leptomeningeal disease.
5. Current immunosuppressive therapy including >10mg/day of prednisone within 14 days of enrollment is not permitted. Inhaled or topical steroids, and adrenal replacement steroid doses ≤10 mg daily prednisone equivalent, are permitted in the absence of active autoimmune disease.

6. Patients must not have known active Hepatitis B Virus (HBV) or Hepatitis C Virus (HCV) infection prior to registration.
7. Patients must not have active autoimmune disease that has required systemic treatment in past 2 years from date of enrollment (i.e., with use of disease modifying agents, corticosteroids or immunosuppressive drugs). Replacement therapy (e.g., thyroxine, insulin, or physiologic corticosteroid replacement therapy for adrenal or pituitary insufficiency, etc.) is not considered a form of systemic treatment.
8. Patients must not have a history of (non-infectious) pneumonitis that required steroids or current pneumonitis.
9. Patients must not have received live vaccines within 42 days prior to enrollment. Examples of live vaccines include, but are not limited to, the following: measles, mumps, rubella, chicken pox, shingles, yellow fever, rabies, BCG, and typhoid (oral) vaccine. Seasonal influenza and COVID-19 vaccines for injection are *generally* killed virus vaccines and are allowed; however, intranasal influenza vaccines (e.g., Flu-Mist®) are live attenuated vaccines, and are not allowed.
10. Patients must not have a history or current evidence of any condition, therapy or laboratory abnormality that might confound the trial results, interfere with the patient's participation for the full duration of the trial, or indicate that participation in the trial is not in the patient's best interests, in the opinion of the treating investigator.
11. Patients must not be pregnant or lactating.
12. Treatment with any investigational agent within 30 days of first administration of study treatment is not permitted.

5.3 LIFESTYLE CONSIDERATIONS

5.3.1 DIET

Subjects should maintain a normal diet unless modifications are required to manage an AE such as diarrhea, nausea or vomiting. Food should be withheld for 2 hours prior to and following oral administration of each DEC-C tablet.

5.3.2 CONTRACEPTION

Nivolumab may have adverse effects on a fetus in utero. Furthermore, it is not known if Nivolumab has transient adverse effects on the composition of sperm.

Animal reproduction studies have not been conducted with Nivolumab; however, blockade of PD-L1 signaling has been shown in murine models of pregnancy to disrupt tolerance to the fetus and to result in an increase in fetal loss. These results indicate a potential risk, based on its mechanism of action, that administration of Nivolumab during pregnancy could cause fetal harm, including increased rates of abortion or stillbirth. Human IgG4 is known to cross the placental barrier and Nivolumab is an IgG4; therefore, Nivolumab has the potential to be transmitted from the mother to the developing fetus. Nivolumab is not recommended during pregnancy unless the clinical benefit outweighs the potential risk to the fetus. Women of childbearing potential should use effective contraception during treatment with Nivolumab and for at least 120 days after the last dose of Nivolumab.

For this trial:

- ***Male subjects will be considered to be of non-reproductive potential if*** they have azoospermia (whether due to having had a vasectomy or due to an underlying medical condition).

- ***Female subjects will be considered of non-reproductive potential if they are either:***
  (1) Postmenopausal (defined as at least 12 months with no menses without an alternative medical cause; in women < 45 years of age a high follicle stimulating hormone (FSH) level in the postmenopausal range may be used to confirm a post-menopausal state in women not using hormonal contraception or hormonal replacement therapy. In the absence of 12 months of amenorrhea, a single FSH measurement is insufficient.).
  OR
  (2) have had a hysterectomy and/or bilateral oophorectomy, bilateral salpingectomy or bilateral tubal ligation/occlusion, at least 6 weeks prior to screening;
  OR
  (3) has a congenital or acquired condition that prevents childbearing.

- ***Female and male subjects of reproductive potential must agree to avoid becoming pregnant or impregnating a partner***, respectively, while receiving study drug and for 120 days after the last dose of study drug by complying with one of the following:
  (1) practice abstinence† from heterosexual activity;
  OR
  (2) use (or have their partner use) acceptable contraception during heterosexual activity.

- ***Acceptable methods of contraception are‡:***
  Single method (one of the following is acceptable):
    - intrauterine device (IUD).
    - vasectomy of a female subject's male partner.
    - contraceptive rod implanted into the skin.

  Combination method (requires use of two of the following):
    - diaphragm with spermicide (cannot be used in conjunction with cervical cap/spermicide).
    - cervical cap with spermicide (nulliparous women only).
    - contraceptive sponge (nulliparous women only).
    - male condom or female condom (cannot be used together).
    - hormonal contraceptive: oral contraceptive pill (estrogen/progestin pill or progestin-only pill), contraceptive skin patch, vaginal contraceptive ring, or subcutaneous contraceptive injection.

†Abstinence (relative to heterosexual activity) can be used as the sole method of contraception if it is consistently employed as the subject's preferred and usual lifestyle and if considered acceptable by local regulatory agencies and ERCs/IRBs. Periodic abstinence (e.g., calendar, ovulation, sympto-thermal, post-ovulation methods, etc.) and withdrawal are not acceptable methods of contraception.

‡If a contraceptive method listed above is restricted by local regulations/guidelines, then it does not qualify as an acceptable method of contraception for subjects participating at sites in this country/region.

Subjects should be informed that taking the study medication may involve unknown risks to the fetus (unborn baby) if pregnancy were to occur during the study. In order to participate in the study subjects of childbearing potential must adhere to the contraception requirement (described above) from the day of study medication initiation (or 14 days prior to the initiation of study medication for oral contraception) throughout the study period up to 120 days after the last dose of trial therapy. If there is any question that a subject of childbearing potential will not reliably comply with the requirements for contraception, that subject should not be entered into the study.

5.3.3 USE IN PREGNANCY

If a subject inadvertently becomes pregnant while on treatment with Nivolumab, the subject will immediately be removed from the study. The site will contact the subject at least monthly and document the subject's status until the pregnancy has been completed or terminated. The outcome of the pregnancy will be reported to the Sponsor-Investigator, DSMC, COMIRB, and within 2 working days to NCCN/Taiho Oncology, Inc. if the outcome is a serious adverse experience (e.g., death, abortion, congenital anomaly, or other disabling or life-threatening complication to the mother or newborn).

The study investigator will make every effort to obtain permission to follow the outcome of the pregnancy and report the condition of the fetus or newborn to the Sponsor-Investigator, DSMC, COMIRB, and to NCCN/Taiho Oncology, Inc. If a male subject impregnates his female partner the study personnel at the site must be informed immediately and the pregnancy reported to the investigator, DSMC, COMIRB, and to NCCN/Taiho Oncology, Inc.

5.3.4 USE IN NURSING WOMEN

It is unknown, but likely, that Nivolumab is excreted in human milk. Since many drugs are excreted in human milk, and because of the potential for serious adverse reactions in the nursing infant, subjects who are breast-feeding are not eligible for enrollment.

5.4 SCREEN FAILURES

Subject may be re-screened for eligibility with documented approval from the PI.

5.5 STRATEGIES FOR RECRUITMENT AND RETENTION

Potential subjects will be identified and recruited from the research study team's Center for Rare Melanomas. The consent process will be conducted in a private room. Potential subjects will be given a copy of the consent form to take home with them before making a final decision to participate.

The target sample size is 19, and we expect that up to 30 patients will be screened to reach this target. We anticipate an annual enrollment rate of 12 patients per year to reach target enrollment within 18 months.

The study will not enroll participants from vulnerable populations. We expect to enroll an equal number of males and females, but mucosal melanomas commonly develop in the vulvovaginal region and may result in a higher frequency of women compared to men enrolled in the study.

6 STUDY INTERVENTION

6.1 STUDY INTERVENTION(S) ADMINISTRATION

6.1.1 STUDY INTERVENTION DESCRIPTION

Nivolumab (per standard of care) and DEC-C (supplied by Taiho Oncology, Inc.) will both be distributed by the University of Colorado Cancer Center's investigational pharmacy.

6.1.2 DOSING AND ADMINISTRATION

Nivolumab: Nivolumab 480 mg will be administered intravenously as standard of care Q4W. Trial treatment with Nivolumab should be administered on Day 0 of each cycle after all procedures/assessments have been completed as detailed in **Table 1** *Schedule of Events*. All trial treatments will be administered on an outpatient basis.

Nivolumab is to be administered as a 30-minute IV infusion. **NIVOLUMAB MUST NOT BE ADMINISTERED AS AN IV PUSH OR BOLUS.** At the end of the infusion, flush the line with a sufficient quantity of normal saline.

Immune-mediated ARs, including severe and fatal cases, have occurred in patients receiving Nivolumab. Immune-mediated ARs affecting more than one body system can occur simultaneously. In clinical studies, most immune-mediated ARs were reversible and managed with interruptions of Nivolumab, administration of corticosteroids, and/or supportive care.

Consultation with the appropriate medical specialist should be considered when investigating a possible IMAR. These events can occur at times ranging from after the first dose of treatment to several months after the last dose of treatment.

Mild IMARs are usually treated symptomatically and infrequently require dosing delays or discontinuation. Higher grade and persistent lower grade IMARs typically necessitate withholding or discontinuing treatment, and administration of systemic steroids or other immunosuppressive agents (such as tumor necrosis factor blockers) when systemic steroids are not effective.

Early recognition of IMARs and initiation of treatment are critical to reduce the risk of complications because the majority of IMARs are reversible with the use of steroids and other immune suppressants. Therefore, early recognition and initiation of treatment is critical to reduce complications.

For suspected IMARs, ensure adequate evaluation to confirm etiology or exclude other causes. Based on the severity of IMARs, withhold or permanently discontinue Nivolumab and administer corticosteroids. Dose modification and toxicity management guidelines for IMARs associated with Nivolumab are provided in **Table 2**.

Table 2. Dose Modification Guidelines for Nivolumab-Related Adverse Events

General instructions:

1. Corticosteroid taper should be initiated upon AE improving to Grade 1 or less and continue to taper over at least 4 weeks.
2. For situations where Nivolumab has been withheld, Nivolumab can be resumed after AE has been reduced to Grade 1 or 0 and corticosteroid has been tapered. Nivolumab should be permanently discontinued if AE does not resolve within 12 weeks of last dose or corticosteroids cannot be reduced to ≤10 mg prednisone or equivalent per day within 12 weeks.
3. For severe and life-threatening IMARs, IV corticosteroid should be initiated first followed by oral steroid. Other immunosuppressive treatment should be initiated if IMARs cannot be controlled by corticosteroids.

**Immune-mediated ARs**	**Toxicity grade or conditions (CTCAEv5.0)**	**Action taken to Nivolumab**	**IMAR management with corticosteroid and/or other therapies**	**Monitor and follow-up**
Pneumonitis	Grade 2	Withhold.	• Administer corticosteroids (initial dose of 1-2 mg/kg prednisone or equivalent) followed by taper.	• Monitor participants for signs and symptoms of pneumonitis.<br>• Evaluate participants with suspected pneumonitis with radiographic imaging and initiate corticosteroid treatment.<br>• Add prophylactic antibiotics for opportunistic infections.
	Grade 3 or 4, or recurrent Grade 2	Permanently discontinue.		
Diarrhea / Colitis	Grade 2 or 3	Withhold.	• Administer corticosteroids (initial dose of 1-2 mg/kg prednisone or equivalent) followed by taper.	• Monitor participants for signs and symptoms of enterocolitis (ie, diarrhea, abdominal pain, blood or mucus in stool with or without fever) and of bowel perforation (ie, peritoneal signs and ileus).<br>• Participants with ≥ Grade 2 diarrhea suspecting colitis should consider GI consultation and performing endoscopy to rule out colitis.<br>• Participants with diarrhea/colitis should be advised to drink liberal quantities of clear fluids. If sufficient oral fluid intake is not feasible, fluid and electrolytes should be substituted via IV infusion.
	Grade 4	Permanently discontinue.		

General instructions:

1. Corticosteroid taper should be initiated upon AE improving to Grade 1 or less and continue to taper over at least 4 weeks.
2. For situations where Nivolumab has been withheld, Nivolumab can be resumed after AE has been reduced to Grade 1 or 0 and corticosteroid has been tapered. Nivolumab should be permanently discontinued if AE does not resolve within 12 weeks of last dose or corticosteroids cannot be reduced to ≤10 mg prednisone or equivalent per day within 12 weeks.
3. For severe and life-threatening IMARs, IV corticosteroid should be initiated first followed by oral steroid. Other immunosuppressive treatment should be initiated if IMARs cannot be controlled by corticosteroids.

Immune-mediated ARs	Toxicity grade or conditions (CTCAEv5.0)	Action taken to Nivolumab	IMAR management with corticosteroid and/or other therapies	Monitor and follow-up
AST / ALT elevation or Increased bilirubin	Grade 2	Withhold.	Administer corticosteroids (initial dose of 0.5- 1 mg/kg prednisone or equivalent) followed by taper.	Monitor with liver function tests (consider weekly or more frequently until liver enzyme value returned to baseline or is stable.
AST / ALT elevation or Increased bilirubin<br><br>Type 1 diabetes mellitus (T1DM) or Hyperglycemia	Grade 3 or 4	Permanently discontinue.	• Administer corticosteroids (initial dose of 1-2 mg/kg prednisone or equivalent) followed by taper.	• Monitor with liver function tests (consider weekly or more frequently until liver enzyme value returned to baseline or is stable.<br>• Monitor participants for hyperglycemia or other signs and symptoms of diabetes.
	Newly onset T1DM or Grade 3 or 4 hyperglycemia associated with evidence of β-cell failure	Withhold.	• Administer insulin for participants with T1DM .<br>• Administer anti-hyperglycemic in participants with hyperglycemia until metabolic control is achieved.	
Hypophysitis	Grade 2	Withhold.	• Administer corticosteroids to treat adrenal insufficiency and other hormone replacements as clinically indicated.	• Monitor patients for signs and symptoms of adrenal insufficiency and hypophysitis (including hypopituitarism).
Hypophysitis<br><br>Hyperthyroidism	Grade 3 or 4	Withhold or permanently discontinue[1].	• Administer corticosteroids to treat adrenal insufficiency and other hormone	• Monitor patients for signs and symptoms of adrenal insufficiency and hypophysitis (including hypopituitarism).

General instructions:

1. Corticosteroid taper should be initiated upon AE improving to Grade 1 or less and continue to taper over at least 4 weeks.
2. For situations where Nivolumab has been withheld, Nivolumab can be resumed after AE has been reduced to Grade 1 or 0 and corticosteroid has been tapered. Nivolumab should be permanently discontinued if AE does not resolve within 12 weeks of last dose or corticosteroids cannot be reduced to ≤10 mg prednisone or equivalent per day within 12 weeks.
3. For severe and life-threatening IMARs, IV corticosteroid should be initiated first followed by oral steroid. Other immunosuppressive treatment should be initiated if IMARs cannot be controlled by corticosteroids.

Immune-mediated ARs	Toxicity grade or conditions (CTCAEv5.0)	Action taken to Nivolumab	IMAR management with corticosteroid and/or other therapies	Monitor and follow-up
	Grade 2	Continue.	replacements as clinically indicated.<br>• Treat with non-selective beta-blockers (eg, propranolol) or thionamides as appropriate.	• Monitor for signs and symptoms of thyroid disorders.
Hyperthyroidism<br><br>Hypothyroidism	Grade 3 or 4	Withhold or permanently discontinue[1].	• Treat with non-selective beta-blockers (eg, propranolol) or thionamides as appropriate.<br>• Initiate thyroid replacement hormones (eg, levothyroxine or liothyroinine) per standard of care.	• Monitor for signs and symptoms of thyroid disorders.<br>• Monitor for signs and symptoms of thyroid disorders.<br>•
	Grade 2-4	Continue.		
Nephritis and Renal dysfunction	Grade 2	Withhold.	Administer corticosteroids (prednisone 1-2 mg/kg or equivalent) followed by taper.	• Monitor changes of renal function.
Nephritis and Renal dysfunction	Grade 3 or 4	Permanently discontinue.	• Administer corticosteroids (prednisone 1-2 mg/kg or equivalent) followed by taper.<br>• Based on severity of AE administer corticosteroids.	• Monitor changes of renal function.<br>• Ensure adequate evaluation to confirm etiology and/or exclude other causes.
Myocarditis	Grade 1 or 2	Withhold.		
Myocarditis	Grade 3 or 4	Permanently discontinue.	• Based on severity of AE administer corticosteroids.	• Ensure adequate evaluation to confirm etiology and/or exclude other causes.

General instructions:

1. Corticosteroid taper should be initiated upon AE improving to Grade 1 or less and continue to taper over at least 4 weeks.
2. For situations where Nivolumab has been withheld, Nivolumab can be resumed after AE has been reduced to Grade 1 or 0 and corticosteroid has been tapered. Nivolumab should be permanently discontinued if AE does not resolve within 12 weeks of last dose or corticosteroids cannot be reduced to ≤10 mg prednisone or equivalent per day within 12 weeks.
3. For severe and life-threatening IMARs, IV corticosteroid should be initiated first followed by oral steroid. Other immunosuppressive treatment should be initiated if IMARs cannot be controlled by corticosteroids.

**Immune-mediated ARs**	**Toxicity grade or conditions (CTCAEv5.0)**	**Action taken to Nivolumab**	**IMAR management with corticosteroid and/or other therapies**	**Monitor and follow-up**
Severe skin reactions		Withhold or permanently discontinue based on the type of event. Events that require discontinuation include and not limited to: Stevens-Johnson Syndrome (SJS) and toxic epidermal necrolysis (TEN).	• Administer corticosteroids.	Monitor patients for suspected severe skin reactions and exclude other causes.
All other immune-mediated ARs	Intolerable/ persistent Grade 2	Withhold.	• Based on type and severity of AE administer corticosteroids.	• Ensure adequate evaluation to confirm etiology and/or exclude other causes.<br>•
1. Withhold or permanently discontinue Nivolumab is at the discretion of the investigator or treating physician.<br>**NOTE:** For participants with Grade 3 or 4 immune-mediatedendo crinopathy where withhold of Nivolumab is required, Nivolumab may be	Grade 3	Withhold or discontinue based on the type of event. Events that require discontinuation include and not limited to: Guillain-Barre Syndrome, pancreatitis, encephalitis, sarcoidosis, myasthenic syndrome-myasthenia gravis (including exacerbation), and myelitis.	• Based on type and severity of AE administer corticosteroids.	• Ensure adequate evaluation to confirm etiology and/or exclude other causes.
	Grade 4 or recurrent Grade 3	Permanently discontinue.		

General instructions:

1. Corticosteroid taper should be initiated upon AE improving to Grade 1 or less and continue to taper over at least 4 weeks.
2. For situations where Nivolumab has been withheld, Nivolumab can be resumed after AE has been reduced to Grade 1 or 0 and corticosteroid has been tapered. Nivolumab should be permanently discontinued if AE does not resolve within 12 weeks of last dose or corticosteroids cannot be reduced to ≤10 mg prednisone or equivalent per day within 12 weeks.
3. For severe and life-threatening IMARs, IV corticosteroid should be initiated first followed by oral steroid. Other immunosuppressive treatment should be initiated if IMARs cannot be controlled by corticosteroids.

**Immune-mediated ARs**	**Toxicity grade or conditions (CTCAEv5.0)**	**Action taken to Nivolumab**	**IMAR management with corticosteroid and/or other therapies**	**Monitor and follow-up**
resumed when AE resolves to ≤ Grade 2 and is controlled with hormonal replacement therapy or achieved metabolic control (in case of T1DM).				

1. Withhold or permanently discontinue Nivolumab is at the discretion of the investigator or treating physician.

NOTE:
For participants with Grade 3 or 4 immune-mediated endocrinopathy where withhold of Nivolumab is required, Nivolumab may be resumed when AE resolves to ≤ Grade 2 and is controlled with hormonal replacement therapy or achieved metabolic control (in case of T1DM).

Management of Nivolumab infusion reactions: Signs and symptoms usually develop during or shortly after drug infusion and generally resolve completely within 24 hours of completion of infusion.

Following are treatment guidelines (**Table 3**) for subjects who experience an infusion reaction associated with administration of Nivolumab.

Table 3. Infusion Reaction Treatment Guidelines

NCI CTCAE Grade	Treatment	Premedication at subsequent dosing
<u>Grade 1</u><br>Mild reaction; infusion interruption not indicated; intervention not indicated	Increase monitoring of vital signs as medically indicated until the subject is deemed medically stable in the opinion of the investigator.	None.
<u>Grade 2</u><br>Requires infusion interruption but responds promptly to symptomatic treatment (e.g., antihistamines, NSAIDS, narcotics, IV fluids); prophylactic medications indicated for < =24 hrs	**Stop Infusion and monitor symptoms.**<br>Additional appropriate medical therapy may include but is not limited to:<br>IV fluids<br>Antihistamines<br>NSAIDS<br>Acetaminophen<br>Narcotics<br>Increase monitoring of vital signs as medically indicated until the subject is deemed medically stable in the opinion of the investigator.<br>If symptoms resolve within one hour of stopping drug infusion, the infusion may be restarted at 50% of the original infusion rate (e.g., from 100 mL/hr to 50 mL/hr). Otherwise dosing will be held until symptoms resolve and the subject should be premedicated for the next scheduled dose.<br>**Subjects who develop Grade 2 toxicity despite adequate premedication should be permanently discontinued from further trial treatment administration.**	Subject may be premedicated 1.5h (± 30 minutes) prior to infusion of Nivolumab with:<br><br>Diphenhydramine 50 mg po (or equivalent dose of antihistamine).<br><br>Acetaminophen 500-1000 mg po (or equivalent dose of antipyretic).
<u>Grades 3 or 4</u><br>Grade 3:<br>Prolonged (i.e., not rapidly responsive to symptomatic medication and/or brief interruption of infusion); recurrence of symptoms following initial improvement; hospitalization indicated for other clinical sequelae (e.g., renal impairment, pulmonary infiltrates)<br><br>Grade 4:<br>Life-threatening; pressor or ventilatory support indicated	**Stop Infusion.**<br>Additional appropriate medical therapy may include but is not limited to:<br>IV fluids<br>Antihistamines<br>NSAIDS<br>Acetaminophen<br>Narcotics<br>Oxygen<br>Pressors<br>Corticosteroids<br>Epinephrine<br><br>Increase monitoring of vital signs as medically indicated until the subject is deemed medically stable in the opinion of the investigator.<br>Hospitalization may be indicated.<br>**Subject is permanently discontinued from further trial treatment administration.**	No subsequent dosing.
Appropriate resuscitation equipment should be available in the room and a physician readily available during the period of drug administration.		

DEC-C: DEC-C will be administered by patients at home on day -5, -4, -3, -2, and -1 (starting dose), with each cycle of Nivolumab. DEC-C will be administered once a day (35 mg Decitabine/100 mg Cedazuridine) on an empty stomach (patient is to withhold food for 2 hours prior to and after administration). In the event Nivolumab treatment is delayed, DEC-C administration will concomitantly delayed.

If patients experience dose limiting toxicities (DLTs), grade 3 or 4 toxicity that is believed to be related to the DEC-C, then both DEC-C and Nivolumab will be held until the toxicity resolves to grade 0 or 1. In the case where the grade 3 or 4 toxicity is believed to be related to Nivolumab, the dose modification guidelines in **Table 2** will be followed. If the toxicity appears to be related to a combination of Nivolumab and DEC-C, the DEC-C will be held and the guidelines in **Table 2** will be followed. After the toxicity has resolved, the DEC-C will be restarted at the next scheduled time at a *reduced dose* of 3 consecutive days per Q4W.

The choice of a de-escalation design was based on concern for the potential toxicity associated with the combination of Decitabine and Nivolumab. The standard dosing for Decitabine is 5 consecutive days per 28-day cycle, but one study compared 3 consecutive days per 28-day cycle to the standard 5 days and found similar efficacy (*30*). The de-escalation design was intended to aim for the effect on demethylation and RIG-I pathway activation yet allow for a dose reduction for side effects.

Interruptions: Dosing interruptions are permitted in the case of medical / surgical events or logistical reasons not related to study therapy (e.g., elective surgery, unrelated medical events, patient vacation, and/or holidays). Subjects should be placed back on study therapy within 3 weeks of the scheduled interruption, unless otherwise discussed with the Sponsor-Investigator. The reason for interruption should be documented in the patient's study record.

6.2 PREPARATION/HANDLING/STORAGE/ACCOUNTABILITY

6.2.1 ACQUISITION AND ACCOUNTABILITY

The study agents will be dispensed by the University of Colorado Cancer Center's investigational pharmacy. DEC-C, provided by Taiho Oncology, Inc, will be dispensed in the clinic on Cycle 1 Day -5 for Cycle 1, Cycle 1 Day 0 for Cycle 2, Cycle 2 Day 0 for Cycle 3 and Cycle 3 Day 0 for Cycle 4, etc. Nivolumab will be dispensed in the clinic on Day 0 of each cycle.

The Sponsor-Investigator is responsible for keeping accurate records of the clinical supplies received from NCCN/Taiho Oncology, Inc., or designee, the amount of DEC-C and Nivolumab dispensed to and returned by the subjects and the amount remaining at the conclusion of the trial.

Upon completion or termination of the study, all unused and/or partially used investigational product (study drug) will be destroyed at the site per institutional policy. It is the Sponsor-Investigator's responsibility to arrange for disposal of all empty containers, provided that procedures for proper disposal have been established according to applicable federal, state, local

and institutional guidelines and procedures, and provided that appropriate records of disposal are kept.

6.2.2 FORMULATION, APPEARANCE, PACKAGING, AND LABELING

DEC-C will be supplied as 35mg/100 mg Decitabine/Cedazuridine tablets. The tablets are biconvex, oval-shaped, film-coated, red and debossed with "H35" on one side.

Nivolumab is a sterile, preservative-free, non-pyrogenic, clear to opalescent, colorless to pale-yellow liquid that may contain light (few) particles. Nivolumab injection for intravenous infusion is supplied in single-dose vials.

6.2.3 PRODUCT STORAGE AND STABILITY

Clinical supplies must be stored in a secure, limited-access location under the storage conditions specified on the label. Receipt and dispensing of trial medication must be recorded by an authorized person at the trial site.

Clinical supplies may not be used for any purpose other than that stated in the protocol.

DEC-C should be stored at room temperature, 20°C to 25°C (68°F to 77°F); excursions permitted from 15°C to 30°C (59°F to 86°F).

Nivolumab does not contain a preservative. After preparation, store the Nivolumab infusion either:

- at room temperature for no more than 8 hours from the time of preparation. This includes room temperature storage of the infusion in the IV container and time for administration of the infusion.
- under refrigeration at 2°C to 8°C (36°F to 46°F) for no more than 24 hours from the time of infusion preparation. Do not freeze.

6.2.4 PREPARATION

DEC-C will be supplied as a tablet and no preparation is required.

Nivolumab preparation:

- Withdraw the required volume of Nivolumab and transfer into an intravenous container.
- Dilute Nivolumab with either 0.9% Sodium Chloride Injection, USP or 5% Dextrose Injection, USP to prepare an infusion with a final concentration ranging from 1 mg/mL to 10 mg/mL.
- Mix diluted solution by gentle inversion. Do not shake.
- Discard partially used vials or empty vials of Nivolumab.

6.3 MEASURES TO MINIMIZE BIAS: RANDOMIZATION AND BLINDING

This is an open-label, single-arm trial; therefore, the Sponsor-Investigator, study personnel, and subject will know the treatment administered.

6.4 STUDY INTERVENTION COMPLIANCE

Participants will be required to keep a drug diary to monitor at home administration of DEC-C.

6.5 PROHIBITED THERAPY

Medications or vaccinations specifically prohibited in the exclusion criteria are not allowed during the ongoing trial. If there is a clinical indication for one of these or other medications or vaccinations specifically prohibited during the trial, discontinuation from trial therapy or vaccination may be required. The final decision on any supportive therapy or vaccination rests with the Sponsor-Investigator and/or the subject's primary physician.

Subjects are prohibited from receiving the following therapies during the Screening and Treatment Phase (including retreatment for post-complete response relapse) of this trial:

- Antineoplastic systemic chemotherapy or biological therapy
- Immunotherapy not specified in this protocol.
- Chemotherapy not specified in this protocol.
- Radiation therapy
  - Note: Radiation therapy to a symptomatic solitary lesion or to the brain may be allowed at the investigator's discretion.
- Live vaccines within 30 days prior to the first dose of trial treatment and while participating in the trial. Examples of live vaccines include, but are not limited to, the following: measles, mumps, rubella, varicella/zoster, yellow fever, rabies, BCG, and typhoid.
- Systemic glucocorticoids for any purpose other than to modulate symptoms from an event of clinical interest of suspected immunologic etiology. The use of physiologic doses of corticosteroids may be approved after consultation with the Sponsor-Investigator.

Subjects who, in the assessment by the investigator, require the use of any of the aforementioned treatments for clinical management should be removed from the trial. Subjects may receive other medications that the investigator deems to be medically necessary.

The Exclusion Criteria describes other medications that are prohibited in this trial.

There are no prohibited therapies during the Post-Treatment Follow-up Phase.

6.6 CONCOMITANT THERAPY

All treatments that the investigator considers necessary for a subject's welfare may be administered at the discretion of the investigator in keeping with the community standards of medical care. All concomitant medication will be recorded on the case report form (CRF) including all prescription, over the counter (OTC), herbal supplements, and IV medications. If changes occur during the trial period, documentation of drug dosage, frequency, route, and date may also be included on the CRF.

All concomitant medications received within 28 days before the first dose of trial treatment and 30 days after the last dose of trial treatment should be recorded. Concomitant medications administered after 30 days after the last dose of trial treatment should be recorded for SAEs.

6.6.1 RESCUE MEDICINE

Subjects should receive appropriate supportive care measures as deemed necessary by the treating investigator. Suggested supportive care measures for the management of adverse events with potential immunologic etiology are outlined below. Where appropriate, these guidelines include the use of oral or intravenous treatment with corticosteroids as well as additional anti-inflammatory agents if symptoms do not improve with administration of corticosteroids. Note that several courses of steroid tapering may be necessary as symptoms may worsen when the steroid dose is decreased. For each disorder, attempts should be made to rule out other causes such as metastatic disease or bacterial or viral infection, which might require additional supportive care. The treatment guidelines are intended to be applied when the investigator determines the events to be related to Nivolumab.

7 STUDY INTERVENTION DISCONTINUATION AND PARTICIPANT DISCONTINUATION/WITHDRAWAL

7.1 DISCONTINUATION OF STUDY INTERVENTION (STOPPING RULES)

The Sponsor-Investigator has the right to terminate this study at any time. Reasons for terminating the study may include, but are not limited to, the following:

- The incidence or severity of adverse events in this or other studies indicates a potential health hazard to patients.
- Patient enrollment is unsatisfactory.

7.1.1 DISCONTINUATION OF STUDY THERAPY AFTER CR

Discontinuation of treatment may be considered for participants who have attained a confirmed Complete Response (CR) that have had at least two treatments with Nivolumab beyond the date when the initial CR was declared. Subjects who then experience radiographic disease progression may be eligible for retreatment with Nivolumab (480 mg Q4W) for up to one additional year.

7.1.2 DISCONTINUATION OF STUDY THERAPY AFTER DISEASE STABILITY

Participants who achieve a partial response and prolonged disease stability may stay on Nivolumab (480 mg Q4W) for up to two years on study. In the absence of significant toxicity, the study drug may be discontinued at the discretion of the treating medical oncologist or Sponsor-Investigator after a prolonged period of disease stability. Subjects who then experience radiographic disease progression may be eligible for retreatment (480 mg Q4W) for up to one additional year.

7.1.3 TOXICITY AND STUDY DISCONTINUATION CRITERIA

The trial stopping criteria outlined in the Calculated Trial Stopping Criteria (**Table 6-2**) will be followed to avoid posing undue risk to additional patients. Nine-twelve patients will potentially be recruited in our study to evaluate DLTs and suggested RP2D. Toxicity is defined as AEs of grade 3 or higher, heme grade 4 or higher. Historical data of similar patients show an overall grade 3 or 4 toxicity rate of approximately 23% for Nivolumab treatment alone (*31*). It is estimated that the current trial (the combination) may potentially increase toxicity. The DSMC will have oversight responsibilities for reviewing toxicities in patients associated with this trial. Subjects who discontinue DEC-C treatment due to toxicity will be allowed to continue treatment with Nivolumab alone if the investigator feels the subject will continue to derive benefit from the treatment.

7.2 PARTICIPANT DISCONTINUATION/WITHDRAWAL FROM STUDY

Participants are free to withdraw from participation in the study at any time upon request. In addition, the investigator has the right to withdraw a patient from the study at any time. Reasons for withdrawal from the study may include, but are not limited to, the following:

- Patient withdrawal of consent at any time.
- Any medical condition that the Sponsor-Investigator determines may jeopardize the patient's safety if he or she continues in the study.
- Sponsor-Investigator determines it is in the best interest of the patient.
- Patient non-compliance.

Patients must discontinue study treatment if they experience any of the following:

- The subject or legal representative (such as a parent or legal guardian) withdraws consent.
- Confirmed radiographic disease progression (i.e., progressive disease per RECIST as documented by tumor imaging at any time point). Subjects with disease progression indicated on imaging should discontinue all study treatment but may continue Nivolumab according to standard of care outside of the study at investigator discretion. Subjects with suspected clinical progression may continue study treatment at investigator discretion until there is radiographic confirmation of disease progression.
- Unacceptable adverse experiences.
- Intercurrent illness that prevents further administration of treatment.
- Investigator's decision to withdraw the subject.
- The subject has a confirmed positive serum pregnancy test.

- Noncompliance with trial treatment or procedure requirements.
- The subject is lost to follow-up.
- Administrative reasons.

Every effort should be made to obtain information on patients who withdraw from the study. The primary reason for withdrawal from the study or study drug discontinuation should be documented on the appropriate eCRF.

7.3 LOST TO FOLLOW-UP

A subject will be considered lost to follow-up if he or she fails to return for 3 scheduled visits and is unable to be contacted by the study staff.

The following actions must be taken if a subject fails to return to the clinic for a required study visit:

- Site will attempt to contact the subject and reschedule the missed visit and advise subject on importance of maintaining assigned visit schedule.
- Before a subject is deemed lost to follow-up, the investigator will make every effort to regain contact with the subject. These contact attempts should be documented in the subject's medical record or study file.
- Should the subject continue to be unreachable, he or she will be considered to have withdrawn from the study.

8 STUDY ASSESSMENTS AND PROCEDURES

8.1 EFFICACY ASSESSMENTS

The efficacy analysis population consists of all subjects who receive any DEC-C treatment. We will monitor efficacy via two endpoints (1) patient response rates per RECIST criteria, and (2) progression free survival (PFS) and overall survival (OS).

For the end point overall response rate (ORR), ORR will be summarized for patients on RP2D and 95% exact confidence interval provided. For the secondary endpoints of PFS and OS, Kaplan-Meier product-limit method will be used to estimate the PFS and OS and median time and associated 95% CI reported.

8.2 SAFETY AND OTHER ASSESSMENTS

All patients who received any protocol therapy will be employed for safety analyses. Adverse Event (AE)/Safety analysis will be performed on all patients in the Full Analysis Set. Patient incidence of all treatment-emergent AEs will be tabulated by system organ class and preferred term. Tables of fatal AEs, serious AEs, treatment-related AEs, and AEs leading to withdrawal from investigation product will tabulated and provided. For select laboratory parameters, changes of

laboratory values over time (e.g., change from baseline summary statistics), grade shifts in laboratory values from baseline to worst on-study value, and grade 3 or higher laboratory toxicities will be summarized. The ECG measurements from this study will be performed as per standard of care for routine safety monitoring. The MedDRA will be used to code adverse events and the NCI CTCAE version 5.0 will be used to grade severity of adverse events and laboratory toxicities.

Summary statistic will be provided for total number of subjects treated under each dose, average dose administered, and duration of treatment.

8.3 STUDY PROCEDURES

Section 1.3 - *Schedule of Events Table 1* - summarizes the trial procedures to be performed at each visit. Individual trial procedures are described in detail below. It may be necessary to perform these procedures at unscheduled time points if deemed clinically necessary by the investigator.

Furthermore, additional evaluations/testing may be deemed necessary by the Sponsor/Principal-Investigator or treating physician for reasons related to subject safety. In some cases, such evaluation/testing may be potentially sensitive in nature (e.g., HIV, Hepatitis C, etc.), and thus local regulations may require that additional informed consent be obtained from the subject. In these cases, such evaluations/testing will be performed in accordance with those regulations.

All trial procedures will be performed by the investigator, or a qualified designee (also an IRB-approved research personnel for the study).

8.3.1 ADMINISTRATIVE PROCEDURES

8.3.1.1 MEDICAL HISTORY

A medical history will be obtained and will include all active conditions, and any condition diagnosed within the prior 10 years that are considered to be clinically significant by the investigator. Details regarding the disease for which the subject has enrolled in this study will be recorded separately and not listed as medical history.

8.3.1.2 PRIOR MEDICATIONS

Prior medication taken by the subject within 28 days before the first dose of trial treatment, including any protocol-specified washout requirement, will be reviewed and recorded. Treatment for the disease for which the subject has enrolled in this study will be recorded separately and not listed as a prior medication.

8.3.1.3 CONCOMITANT MEDICATIONS

Medication, if any, taken by the subject during the trial will be recorded. All medications related to reportable SAEs should be recorded.

8.3.1.4 DISEASE DETAILS AND TREATMENTS

Disease details: Prior and current details regarding disease status will be obtained. All prior cancer treatments including systemic treatments, radiation and surgeries will be reviewed. All new anti-neoplastic therapy initiated after the last dose of trial treatment will be reviewed. If a subject initiates a new anti-cancer therapy within 30 days after the last dose of trial treatment, the 30-day Safety Follow-up visit must occur before the first dose of the new therapy. Once new anti-cancer therapy has been initiated, the subject will move into survival follow-up.

8.3.2 CLINICAL ASSESSMENTS

8.3.2.1 FULL PHYSICAL EXAM

The investigator or qualified designee will perform a complete physical exam during the screening period. Clinically significant abnormal findings should be recorded as medical history.

8.3.2.2 DIRECTED PHYSICAL EXAM

For cycles that do not require a full physical exam per the *Trial Schedule of Events Chart (Section 7.1)*, the investigator or qualified designee will perform a directed physical exam as clinically indicated prior to trial treatment administration.

8.3.2.3 VITAL SIGNS

The investigator or qualified designee will take vital signs at screening and at treatment discontinuation. Vital signs should include weight and other vital signs per standard of care.

8.3.2.4 EASTERN COOPERATIVE ONCOLOGY GROUP (ECOG) PERFORMANCE SCALE

The investigator or qualified designee will assess ECOG status at screening (**Table 4**), prior to the administration of each dose of trial treatment and discontinuation of trial treatment as specified in the *Schedule of Events Table 1 (Section 1.3)*.

Table 4. ECOG Performance Status

Grade	Description
0	Normal activity. Fully active, able to carry on all pre-disease performance without restriction.
1	Symptoms, but ambulatory. Restricted in physically strenuous activity, but ambulatory and able to carry out work of a light or sedentary nature (e.g., light housework, office work).
2	In bed <50% of the time. Ambulatory and capable of all self-care, but unable to carry out any work activities. Up and about more than 50% of waking hours.
3	In bed >50% of the time. Capable of only limited self-care, confined to bed or chair more than 50% of waking hours.
4	100% bedridden. Completely disabled. Cannot carry on any self-care. Totally confined to bed or chair.
5	Dead.

* As published in Am. J. Clin. Oncol.: *Oken, M.M., Creech, R.H., Tormey, D.C., Horton, J., Davis, T.E., McFadden, E.T., Carbone, P.P.: Toxicity And Response Criteria Of The Eastern Cooperative Oncology Group. Am J Clin Oncol 5:649-655, 1982.* The Eastern Cooperative Oncology Group, Robert Comis M.D., Group Chair.

8.3.3 TUMOR TISSUE COLLECTION AND CORRELATIVE STUDIES BLOOD SAMPLING

Plasma will be banked from every patient for cytokine analysis, potential biomarker and other correlative studies. Plasma will also be collected for potential analysis of plasma DEC-C levels in the event this could correlate with results from the immunologic analysis. Biopsies will be collected at designated intervals specified in **Table 1** and, whenever possible, additional tumor samples will be cataloged and stored at the CU Melanoma Biorepository. Additional correlative studies may include genomic and transcriptomic analysis.

8.3.3.1 TISSUE COLLECTION

Fresh tissue will be collected prior to treatment initiation and post-treatment at around Cycle 2. Tumor tissue will be obtained out-patient by fine needle aspiration, core biopsy, punch biopsy, or scratch biopsy under local anesthesia. Every effort will be made to obtain adequate tissue from the tumor to allow for analysis. Tissue will be used to perform flow cytometry analysis of circulating immune cells and cells collected from tumor FNA biopsies (T cells including exhausted T cells and immunosuppressive Tregs, MDSCs, and NK cells).

An archival tissue sample from a previous diagnostic biopsy may also be requested for the study. An FFPE block or 10-15 slides may be requested.

The tissue specimens should be collected and processed according to the specimen collection forms and protocols.

8.3.4 LABORATORY PROCEDURES/ASSESSMENTS

8.3.4.1 STANDARD SAFETY LABORATORY TESTS

Will be obtained as outlined in **Table 1** - *Schedule of Events*.

8.3.4.2 EXPERIMENTAL BLOOD DRAWS

Will be performed at regularly scheduled clinic visits as outlined in **Table 1** - *Schedule of Events*. Peripheral blood mononuclear cells (PBMCs) will be isolated from blood.

- **Baseline immune response determination.** Subjects in the study will be asked to provide 40 mL of blood prior to initiating treatment to evaluate baseline immune responses and immune cell frequencies.

- **Immune response monitoring.** Blood samples will be drawn pre-treatment, prior to the administration of the Nivolumab in cycles 2 and 4, and upon progression, for continued measurement of immune cell frequencies.

- **Cytokine analysis.** Up to 10 mL of plasma will be stored for up to five years following study completion for subsequent cytokine analysis.

8.3.4.3 SPECIMEN ANALYSIS

Assays will be performed to quantify circulating and tumor immune cell frequency and activity, global DNA methylation levels, RIG-I pathway activity levels, and immune-mediated protein and cytokine expression.

- **Tumor and circulating immune cell analysis**: Flow cytometry analysis of tumor and circulating immune cells (including exhausted T cells and immunosuppressive Tregs, MDSCs, and NK cells) will be performed on serial patient blood samples and FNA biopsies as previously described (*32*). This will allow us to compare pre- and post- treatment immune cell profiles to assess whether the combination of DEC-C and Nivolumab favorably alters immune cell profiles and if immune profiles correlate with patient responses. Cells recovered from flow cytometry analysis will be used to purify gDNA and RNA for subsequent correlative analyses.

- **Global methylation analysis**: Global methylation levels will be analyzed in peripheral blood mononuclear cells and tumor cells prior to and during treatment by performing LINE-1 qRT-PCR on bisulfite-treated gDNA. This will allow us to determine whether DEC-C is inducing global hypo-methylation as expected.

- **RIG-I innate immune pathway analysis**: Gene expression will be analyzed in tumor cells prior to and during treatment by performing qRT-PCR analysis of RNA for a panel of 20 RIG-I pathway genes.

- **Plasma protein analysis**: Serial patient plasma samples will be analyzed using the Olink proteomics Immune-Response detection panel to quantify the expression of 92 proteins including innate immune sensing pathway cytokines and proteins (TNFa, IL-6, IL-8, etc.) and other immune markers including NK ligands. This data will determine a) whether pre-treatment serum protein levels predict responses to, and b) are increased by the treatment with, the combination of DEC-C and Nivolumab. This data will be compared to Olink proteomics data for treatment-naïve and anti-PD1 only treated mucosal melanoma patients with samples in our melanoma biorepository.

8.3.5 VISIT REQUIREMENTS

Visit requirements are outlined in Section 1.3, **Table 1** - *Schedule of Events.*

8.3.5.1 SCREENING PERIOD

There will be a screening period of up to 28 days after subjects sign informed consent.

8.3.5.2 TREATMENT PERIOD

The treatment period is defined as the time that the patients are receiving DEC-C and/or Nivolumab treatment.

8.3.5.3 POST-TREATMENT VISITS

Subjects who discontinue trial treatment will move into the follow-up phase and other treatment decisions will be at the discretion of the treating physician.

The first safety follow-up visit will be conducted for all subjects 30 days after the last dose of trial treatment (DEC-C and/or Nivolumab) or before the initiation of a new anti-cancer treatment, whichever comes first.

Should the subject not initiate a new anti-cancer treatment, the second safety follow-up visit will be conducted 90 days after the last dose of trial treatment.

All AEs that occur prior to the Safety Follow-Up Visits should be recorded. Subjects with an AE of Grade > 1 will be followed until the resolution of the AE to Grade 0-1 or until the beginning of a new anti-neoplastic therapy, whichever occurs first.

SAEs that occur within 90 days of the end of treatment or before initiation of a new anti-cancer treatment should also be followed and recorded.

Subjects will be followed for up to two years following end of treatment, or until recurrence, death, withdrawal of consent, or the end of the study, whichever occurs first. Every effort should be made to collect information regarding disease status and post-study anti-neoplastic treatment.

8.4 ADVERSE EVENTS AND SERIOUS ADVERSE EVENTS

8.4.1 DEFINITION OF ADVERSE EVENTS (AE)

Adverse event means any untoward medical occurrence associated with the use of an intervention in humans, whether or not considered intervention-related (21 CFR 312.32 (a)).

For the purposes of this Agreement an Adverse Event (AE) shall mean any untoward medical occurrence whether or not thought to have been caused by the study drug(s) or the Clinical Trial procedures.

8.4.2 DEFINITION OF SERIOUS ADVERSE EVENTS (SAE)

Serious Adverse Event (SAE) shall mean any adverse event that is fatal, life threatening, disabling or incapacitating, requires in-patient hospitalization or prolongs existing hospitalization, is a congenital anomaly in the offspring of the patient or which may require intervention to prevent the previously stated outcomes.

Serious adverse event or **serious suspected adverse reaction**. An AE or suspected adverse reaction is considered "serious" if, in the view of either the investigator or sponsor, it results in any of the following outcomes: death, a life-threatening adverse event, inpatient hospitalization or prolongation of existing hospitalization, a persistent or significant incapacity or substantial disruption of the ability to conduct normal life functions, or a congenital anomaly/ birth defect.

Important medical events that may not result in death, be life-threatening, or require hospitalization may be considered serious when, based upon appropriate medical judgment, they may jeopardize the patient or subject and may require medical or surgical intervention to prevent one of the outcomes listed in this definition. Examples of such medical events include allergic bronchospasm requiring intensive treatment in an emergency room or at home, blood dyscrasias or convulsions that do not result in inpatient hospitalization, or the development of drug dependency or drug abuse.

The Sponsor/Investigator is responsible for the identification, collection, reporting and documentation of AEs/SAEs, as per protocol and institutional policy.

8.4.3 CLASSIFICATION OF AN ADVERSE EVENT

8.4.3.1 SEVERITY OF EVENT

The adverse event severity grading scale for the NCI CTCAE (v5.0, **Table 5**) will be used for assessing adverse event severity. The following table will be used for assessing severity for adverse events that are not specifically listed in the NCI CTCAE (v5.0).

Table 5. NCI CTCAE v5.0 grading scale

Grade	Severity
1	Mild; asymptomatic or mild symptoms; clinical or diagnostic observations only; or intervention not indicated
2	Moderate; minimal, local, or non-invasive intervention indicated; or limiting age-appropriate instrumental activities of daily living [a]
3	Severe or medically significant, but not immediately life-threatening; hospitalization or prolongation of hospitalization indicated; disabling; or limiting self-care activities of daily living [b,c]
4	Life-threatening consequences or urgent intervention indicated
5	Death related to adverse event

NCI CTCAE- National Cancer Institute Common Terminology Criteria for Adverse Events.
Note: Based on the most recent version of NCI CTCAE (v5.0), which can be found at:
http://ctep.cancer.gov/protocolDevelopment/electronic_applications/ctc.htm

[a] Instrumental activities of daily living refer to preparing meals, shopping for groceries or clothes, using the telephone, managing money, etc.

[b] Examples of self-care activities of daily living include bathing, dressing and undressing, feeding oneself, using the toilet, and taking medications, as performed by patients who are not bedridden.

[c] If an event is assessed as a "significant medical event," it must be reported as a serious adverse event.

8.4.3.2 RELATIONSHIP TO STUDY INTERVENTION

For all collected AEs, the clinician who examines and evaluates the participant will determine the AE's causality based on temporal relationship and his/her clinical judgment. The degree of certainty about causality will be graded using the categories below.

- **Definitely Related** – There is clear evidence to suggest a causal relationship, and other possible contributing factors can be ruled out. The clinical event, including an abnormal laboratory test result, occurs in a plausible time relationship to drug administration and cannot be explained by concurrent disease or other drugs or chemicals. The response to withdrawal of the drug (dechallenge) should be clinically plausible. The event must be pharmacologically or phenomenologically definitive, with use of a satisfactory rechallenge procedure if necessary.

- **Probably Related** – There is evidence to suggest a causal relationship, and the influence of other factors is unlikely. The clinical event, including an abnormal laboratory test result, occurs within a reasonable time after administration of the drug, is unlikely to be attributed to concurrent disease or other drugs or chemicals, and follows a clinically reasonable response on withdrawal (dechallenge). Rechallenge information is not required to fulfill this definition.

- **Possibly Related** – There is some evidence to suggest a causal relationship (e.g., the event occurred within a reasonable time after administration of the trial medication). However, other factors may have contributed to the event (e.g., the participant's

clinical condition, other concomitant events). Although an AE may rate only as "possibly related" soon after discovery, it can be flagged as requiring more information and later be upgraded to "probably related" or "definitely related", as appropriate.

- **Unlikely to be Related** – A clinical event, including an abnormal laboratory test result, whose temporal relationship to drug administration makes a causal relationship improbable (e.g., the event did not occur within a reasonable time after administration of the trial medication) and in which other drugs or chemicals or underlying disease provides plausible explanations (e.g., the participant's clinical condition, other concomitant treatments).

- **Not Related** – The AE is completely independent of study drug administration, and/ or evidence exists that the event is definitely related to another etiology. There must be an alternative, definitive etiology documented by the clinician.

8.4.3.3 EXPECTEDNESS

Expectedness will only be documented for SAEs. The treating physician and/or the study PI will be responsible for determining whether an SAE is expected or unexpected. An SAE will be considered unexpected if the nature, severity, or frequency of the event is not consistent with the risk information previously described for the study agent.

8.4.4 TIME PERIOD AND FREQUENCY FOR EVENT ASSESSMENT AND FOLLOW-UP

The occurrence of an AE or SAE may come to the attention of study personnel during study visits and interviews of a study participant presenting for medical care, or upon review by a study monitor. All AEs including local and systemic reactions not meeting the criteria for SAEs will be captured on the appropriate CRF. Information to be collected includes event description, time of onset, clinician's assessment of severity, relationship to study product (assessed only by those with the training and authority to make a diagnosis), and time of resolution/ stabilization of the event. All AEs occurring while on study must be documented appropriately regardless of relationship. All AEs will be followed to adequate resolution.

Any medical condition that is present at the time that the participant is screened will be considered as baseline and not reported as an AE. However, if the study participant's condition deteriorates at any time during the study, it will be recorded as an AE. UAPs will be recorded in the data collection system throughout the study.

Changes in the severity of an AE will be documented to allow an assessment of the duration of the event at each level of severity to be performed. AEs characterized as intermittent require documentation of onset and duration of each episode.

The PI will record all reportable events with start dates occurring any time after informed consent is obtained until 30 days after the last day of study treatment or until initiation of new treatment,

whichever comes first. SAEs will be followed until resolution or stabilization. At each study visit, the investigator will inquire about the occurrence of AE/ SAEs since the last visit.

8.4.5 ADVERSE EVENT REPORTING

The investigator must record non-serious adverse events and report to DSMC and IRB according to timetable for reporting specified in section 10.1.6.

In addition, the following Special Situation Reports should be collected even in the absence of an AE:

A. Drug exposure during pregnancy and lactation, or paternal drug exposure
B. Lack of drug effect
C. Unintended beneficial effect
D. Any suspected transmission of an infectious agent by a medicinal product
E. Product quality complaints associated with possible safety issue(s)
F. Drug or food interaction
G. Overdose
H. Medication error
I. Misuse
J. Occupational exposure

8.4.6 SERIOUS ADVERSE EVENT REPORTING

The Sponsor/Investigator will be responsible for collecting all SAEs, and significant product quality defects that may have consequences for the safety of patients (e.g., suspected transmission of an infectious agent via a medicinal product) from the Study.

Sponsor/Investigator shall record the seriousness, causality and expectedness assessments for each SAE as per Institution's policies and determine the reportability of each assessed SAE. All SAEs considered by the Sponsor/Investigator as unexpected per the Reference Safety Information and assessed as related to the Investigational Product must be reported as a SUSAR by the Sponsor/Investigator to the relevant Regulatory Authorities and ECs in accordance with applicable laws.

All SAEs shall be reported to National Comprehensive Cancer Network (NCCN) and Taiho Oncology, Inc./OTSUKA by completing and submitting a MedWatch form as complete as possible and at the minimum including the following:

A. SAE term(s)
B. Patient identifier
C. Relationship to Company product (Causality)
D. Name of the reporter (Sponsor/Investigator)
E. Seriousness criteria
F. Date of event onset

Information not available at the time of the initial report shall be documented on a follow-up report and submitted to National Comprehensive Cancer Network (NCCN) and Taiho Oncology, Inc./OTSUKA.

The sponsor-investigator must notify FDA and all participating PIs in an IND safety report of potential serious risks as soon as possible, but in no case later than 15 calendar days after the sponsor-investigator determines that the information qualifies for reporting. (21 CFR 312.32(c)(1))

The sponsor-investigator must notify FDA of any unexpected fatal or life-threatening adverse reactions as soon as possible, but in no case later than 7 calendar days after the sponsor-investigator's initial receipt of the information. (21 CFR 312.32(c)(2)).

All SAEs will be followed until satisfactory resolution or until the site PI deems the event to be chronic or the adherence to be stable. Other supporting documentation of the event may be requested by the sponsor-investigator and should be provided as soon as possible.

The sponsor-investigator will be responsible for notifying FDA of any unexpected fatal or life-threatening suspected adverse reaction as soon as possible but in no case later than 7 calendar days after the sponsor-investigator's initial receipt of the information.

All SAEs will be reported using the FDA 3500A Mandatory MedWatch report form unless funding sponsor or drug manufacturer requires use of proprietary form. The SAE form can be found at: http://www.fda.gov/downloads/AboutFDA/ReportsManualsForms/Forms/UCM048334.pdf.

To submit an SAE to study PI, OCRST, and DSMC: E-mail as follows within 24 hours of becoming aware of the event to:

- *martin.mccarter@cuanschutz.edu*
- *cpdm.iit@cuanschutz.edu*
- *DSMC@cuanschutz.edu*

To submit an SAE to National Comprehensive Cancer Network (NCCN) and Taiho Oncology, Inc./OTSUKA: The Sponsor/Investigator shall report to National Comprehensive Cancer Network (NCCN) and Taiho Oncology, Inc./OTSUKA all Serious Adverse Events (SAEs), and Pregnancy in English via a MedWatch form, in accordance with the Protocol and applicable law. All applicable source document(s) will also be sent to National Comprehensive Cancer Network (NCCN) and Taiho Oncology, Inc./OTSUKA in English. The Sponsor/Investigator shall comply with OTSUKA's reasonable follow-up requests.

The Sponsor/Investigator will provide National Comprehensive Cancer Network (NCCN) and Taiho Oncology, Inc./OTSUKA with details of whom National Comprehensive Cancer Network (NCCN) and Taiho Oncology, Inc./OTSUKA shall address requests for follow up information on SAE and pregnancy cases reported from this Clinical Trial, and further agree to update such contact details, as necessary.

Transmission of these reports (initial and follow-up) will be sent electronically via email to the NCCN (ORPreports@nccn.org or fax to 215-358-7699) and Taiho Oncology, Inc. via email (*Global_Intake@otsuka-us.com*) within the timelines specified below:

A. Reporting Clinical Safety Information to National Comprehensive Cancer Network (NCCN) and Taiho Oncology, Inc./OTSUKA:

   a. The investigator shall monitor the Clinical Trial Subject for adverse events and fulfill all the reporting requirements to RAs/ECs in accordance with applicable laws. The Investigator shall inform National Comprehensive Cancer Network (NCCN) and Taiho Oncology, Inc./OTSUKA of serious adverse events:
      - *Unexpected Fatal or Life Threatening Suspected Adverse Reactions*- Complete and send MedWatch to National Comprehensive Cancer Network (NCCN) and Taiho Oncology, Inc/OTSUKA within 24 hours of awareness.
      - *Serious & Unexpected Suspected Adverse Reactions*- Complete and send MedWatch to National Comprehensive Cancer Network (NCCN) and Taiho Oncology, Inc./OTSUKA within 24 hours of awareness.
      - *All other Serious cases (Expected and Related; Expected and Not related; Unexpected and Not related)*- Complete and send MedWatch to National Comprehensive Cancer Network (NCCN) and Taiho Oncology, Inc./OTSUKA within 24 hours of awareness.

   b. All serious adverse events need to be sent to National Comprehensive Cancer Network (NCCN) and Taiho Oncology, Inc./OTSUKA via a MedWatch Form. For reports from a non-English speaking country, English translations be sent to National Comprehensive Cancer Network (NCCN) and Taiho Oncology, Inc./OTSUKA Pharmacovigilance along with a cover sheet.

   c. MedWatch reports must clearly specify SAE term(s) and corresponding Investigator causality assessment.

   d. Otsuka is responsible for submitting post-marketed reports to regulatory authorities via XML/MedWatch/CIOMS.

B. Serious AE reports related or not related to the Product or where the causality is assessed as unknown or not provided shall be transmitted to National Comprehensive Cancer Network (NCCN) and Taiho Oncology, Inc./OTSUKA Pharmacovigilance group within 24 hours of awareness date.

C. Special Situation Reports:

1. <u>Drug Exposure During Pregnancy and Lactation, or Paternal Drug Exposure Reports:</u>

   a. The Sponsor/Investigator will report Exposure During Pregnancy and Lactation, or Paternal Drug Exposure on any Clinical Trial Subject while participating in the Clinical Trial, and following exposure to a TAIHO IMP, to National Comprehensive Cancer Network (NCCN) and Taiho Oncology, Inc./OTSUKA (as specified below) on a MedWatch Form and within 24 hours from first becoming aware of the pregnancy or exposure. If the partner of a Clinical Trial Subject becomes pregnant, the Sponsor/Investigator may collect information about the pregnancy and birth if the partner agrees.

   b. The Subject will also be followed by the Sponsor/Investigator to determine the outcome of the pregnancy (including any premature termination of the pregnancy). Information on the status of the mother and child will be forwarded to National Comprehensive Cancer Network (NCCN) and Taiho Oncology, Inc./OTSUKA. The Sponsor/Investigator must provide final outcome of pregnancy to National Comprehensive Cancer Network (NCCN) and Taiho Oncology, Inc./OTSUKA. If any SAE(s) is observed in the Subject or fetus/child, then SAE(s) must also be reported to National Comprehensive Cancer Network (NCCN) and Taiho Oncology, Inc./OTSUKA.

2. **Other Special Situation Reports** shall be transmitted to National Comprehensive Cancer Network (NCCN) and Taiho Oncology, Inc./OTSUKA within 24 hours of the awareness date.

D. The Sponsor/Investigator shall comply with all applicable laws, including those related to data privacy, when undertaking these safety reporting-related obligations. Any SAE/special situations shall only contain anonymous or key coded information so that any individuals' names or other fully identifiable information shall be redacted before being submitted to National Comprehensive Cancer Network (NCCN) and Taiho Oncology, Inc./OTSUKA. Please note that reporter information is necessary and should not be redacted.

National Comprehensive Cancer Network (NCCN) and Taiho Oncology, Inc./OTSUKA Day 0 for all single case reports (including SUSARs) will be the day National Comprehensive Cancer Network (NCCN) and Taiho Oncology, Inc./OTSUKA is notified by the Sponsor/Investigator and the report meets criteria for reporting.

Each party shall notify the other party within two (2) working days of becoming aware of any of the following urgent safety information: Major safety concerns identified in a clinical trial. Possible teratogenic effects of the Product or other significant hazard to public health. Safety issues published in the scientific and medical literature.

It is understood and agreed that the Sponsor/Investigator will perform adequate due diligence with regards to obtaining follow-up information on incomplete SAE, Special Situation, and pregnancy reports.

Follow-up of unresolved serious adverse events: Any SAEs that are unresolved at the time of the initial report submission should be followed up by the investigator for as long as medically indicated, and an updated SAE report submitted at the time new information regarding the event becomes available.

8.4.7 REPORTING OF PREGNANCY

Although pregnancy and lactation are not considered adverse events, it is the responsibility of investigators or their designees to report any pregnancy or lactation in a subject (spontaneously reported to them) that occurs during the trial.

Pregnancies and lactations that occur after the consent form is signed but before treatment allocation must be reported by the investigator if they cause the subject to be excluded from the trial or are the result of a protocol-specified intervention, including but not limited to washout or discontinuation of usual therapy, diet, placebo treatment or a procedure.

Pregnancies and lactations that occur from the time of treatment allocation through 120 days following cessation of the study drugs, or 30 days following cessation of treatment if the subject initiates new anticancer therapy, whichever is earlier, must be reported by the investigator. All reported pregnancies must be followed to the completion/termination of the pregnancy. Pregnancy outcomes of spontaneous abortion, missed abortion, benign hydatidiform mole, blighted ovum, fetal death, intrauterine death, miscarriage and stillbirth must be reported as serious events (Important Medical Events). If the pregnancy continues to term, the outcome (health of infant) must also be reported. Such events must be reported within 24 hours to the Sponsor-Investigator, within 24 hours to National Comprehensive Cancer Network (NCCN) and Taiho Oncology, Inc./OTSUKA, and to DSMC and COMIRB according to institutional policy.

8.5 UNANTICIPATED PROBLEMS

8.5.1 DEFINITION OF UNANTICIPATED PROBLEMS (UAP)

This study will use the COMIRB definition of UAP. An unanticipated problem is any event or information that was unforeseen and indicates that the research procedures caused harm (including physical, psychological, economic, or social harm) to participants or others or indicates that participants or others are at increased risk of harm than was previously known or recognized.

8.5.2 REPORTING OF UNANTICIPATED PROBLEMS

This study will follow COMIRB's guidance for UAP reporting and the DSMC's requirements. AEs, noncompliance, and protocol violations will be recorded and reported as required either

promptly (within 5 days of Sponsor-Investigator's knowledge) or at the time of the study's continuing review.

It is the responsibility of the PI to report incidents or events that meet the criteria for UAPs reporting to their IRB using the IRB's standard UAP form. The PI is also responsible for reporting the UAP to the UCCC DSMC.

9 STATISTICAL CONSIDERATIONS

9.1 STATISTICAL HYPOTHESES

- Primary Endpoint(s):
    - Determine the safety and tolerability of DEC-C in combination with Nivolumab by evaluating adverse events (AEs) using CTCAEv5.0 criteria and establishing the recommended phase 2 dose (RP2D) of DEC-C.
- Secondary Endpoint(s):
    - Evaluate the rate of subjects that achieve an objective response (ORR) to DEC-C in combination with Nivolumab by radiographic assessment using RECIST criteria.
    - Evaluate and estimate progression free survival (PFS) and overall survival (OS) in unresectable locally advanced and metastatic mucosal melanoma patients at 24 months post-study.

9.2 SAMPLE SIZE DETERMINATION

This study will enroll a maximum of 19-30 patients in phase I/II. The total sample size required for the phase 1 and Phase II will depend on the number of patients treated at the RP2D during Phase 1b, and we estimate the total sample size will be in the range of 19-21 subjects.

Phase Ib: We will enroll a minimum of 9 subjects and maximum 12 subjects into the phase 1b portion of the study.

Phase II: We anticipate a maximum of 9 patients treated at the RP2D in Phase 1b dose de-escalation, and we include those treated at the RP2Din Phase 1b as the first stage in the two-stage design. If there is at least three responses among the 11 patients enrolled in stage 1, the trial will move on to the second stage and enroll additional patients to reach a minimum of 19 patients.

9.3 POPULATION FOR ANALYSES

Patients with stage III unresectable or locally advanced, or Stage IV metastatic, mucosal melanoma. Our Center for Rare Melanomas recruits patients locally, nationally, and internationally.

Accrual Estimates: Our melanoma clinic has recruited an average of 15 new mucosal melanoma cases per year over the past 3 years, and we anticipate this number will increase with the recent establishment of the CU Center for Rare Melanomas. Based on this, we anticipate being able to accrue 1 patient per month. We will therefore require at least 18 months for accrual.

9.4 STATISTICAL ANALYSES

9.4.1 GENERAL APPROACH

The clinical objectives of the study are to characterize the safety and tolerability of DEC-C in combination with Nivolumab, and evaluate the efficacy, with a total of 19-21 evaluable subjects. Cross-sectional imaging will be performed as standard of care. Tumor response will be determined using RECIST. Patients will remain on treatment until unacceptable toxicity, disease progression, or for up to 2 years from study enrollment.

Phase Ib: Time to event Bayesian optimal interval (TITE-BOIN) design will be used to assess the RP2D during phase 1b (*27*). The BOIN design is a model assistant approach used in phase I clinical trial for finding the maximum tolerated dose (MTD). TITE-BOIN design imputes the DLT outcome for patients whose DLT data are pending and accelerate the trial by allowing for real-time dose assignment decisions for new patients. The decision of dose escalation and de-escalation involves comparing the observed dose-limiting toxicity (DLT) rate at the current dose with a pair of fixed dose escalation boundaries and the imputed DLT outcome if there are DLT pending patients. The target toxicity (MTD) rate for this study is set at $\phi = 0.30$. Underdosing bound is set to be 0.236 and overdosing bound is set to be 0.359. There are 2 dose levels where dose of Nivolumab would be fixed at 480 mg intravenously every 4 weeks (Q4W), while DEC-C (35 mg decitabine + 100 mg cedazuridine) orally for 5 days directly prior to each infusion of Nivolumab (day -5, day -4, day -3, day -2, day -1) as first dose level, and DEC-C (35 mg decitabine + 100 mg cedazuridine) orally for 3 days directly prior to each infusion of Nivolumab (day -3, day -2, day -1) as the second dose level. The maximum sample size is 12. If there are nine patients treated on the same dose level, we would terminate the trial early and move to Phase II if the number of response meets the response criterion. We will enroll and treat patients in cohorts of size three. Please see **Table 6-1** for stay/de-escalation rule. The TITE-BOIN design has a built-in stopping rule: stop the trial if the second dose level (the lower dose level) is eliminated due to toxicity, please see Table 6-2 for the stopping rule. In this case, no dose should be selected as the MTD. This rule says that if the lowest dose exceeds a certain safety threshold, we stop the trial for safety.

DLTs will be assessed during the DLT assessment window of 28 days following the first dose of DEC-C and Nivolumab (Cycle 1 Day 0 to Cycle 2 Day 0). Patients who withdraw from the study prior to completing the DLT assessment window for any reason other than a DLT will be replaced. See Section 6.1.2 for DLT definition. The MTD will then be determined at the end of the trial using isotonic regression to pool information across doses, following Liu and Yuan (*33*) and Zhou et al (*34*).

We anticipate up to 9 patients treated at the RP2D in Phase 1b dose de-escalation. If there is at least three responses among the 11 patients during stage 1 (including patients treated at RP2D during Phase Ib), the trial will move on to the second stage and enroll additional patients to reach a minimum of 19 patients.

Table 6-1. Dose stay/de-escalation rule for the TITE-BOIN design.

Escalation/De-escalation Rule

# treated	# DLTs	# Pending patients	Escalation	Stay	De-escalation
3	0	<=1	Yes	No	No
3	0	>=2	Suspend	Suspend	Suspend
3	1	0	No	Yes	No
3	1	1	No	STFT>0.88	STFT<=0.88
3	1	>=2	Suspend	Suspend	Suspend
3	2	<=1	No	No	Yes
3	3	0	No	No	Yes & Eliminate
6	0	<=3	Yes	No	No
6	0	>=4	Suspend	Suspend	Suspend
6	1	<=1	Yes	No	No
6	1	2	STFT>=0.6	STFT<0.6	No
6	1	3	STFT>=1.96	STFT<1.96	No
6	1	>=4	Suspend	Suspend	Suspend
6	2	0	No	Yes	No
6	2	1	No	STFT>0.73	STFT<=0.73
6	2	2	No	STFT>1.8	STFT<=1.8
6	2	3	No	STFT>2.87	STFT<=2.87
6	2	>=4	Suspend	Suspend	Suspend
6	3	<=3	No	No	Yes
6	>=4	<=2	No	No	Yes & Eliminate
9	0	<=4	Yes	No	No
9	0	>=5	Suspend	Suspend	Suspend
9	1	<=4	Yes	No	No
9	1	>=5	Suspend	Suspend	Suspend
9	2	0	Yes	No	No
9	2	1	STFT>=0.59	STFT<0.59	No
9	2	2	STFT>=1.65	STFT<1.65	No
9	2	3	STFT>=2.71	STFT<2.71	No
9	2	4	STFT>=3.77	STFT<3.77	No
9	2	>=5	Suspend	Suspend	Suspend

9	3	0	No	Yes	No
9	3	1	No	STFT>0.58	STFT<=0.58
9	3	2	No	STFT>1.65	STFT<=1.65
9	3	3	No	STFT>2.72	STFT<=2.72
9	3	4	No	STFT>3.79	STFT<=3.79
9	3	>=5	Suspend	Suspend	Suspend
9	4	<=5	No	No	Yes
9	>=5	<=4	No	No	Yes & Eliminate
12	0	<=6	Yes	No	No
12	0	>=7	Suspend	Suspend	Suspend
12	1	<=6	Yes	No	No
12	1	>=7	Suspend	Suspend	Suspend
12	2	<=3	Yes	No	No
12	2	4	STFT>=1.33	STFT<1.33	No
12	2	5	STFT>=2.72	STFT<2.72	No
12	2	6	STFT>=4.11	STFT<4.11	No
12	2	>=7	Suspend	Suspend	Suspend
12	3	<=6	No	Yes	No
12	3	>=7	Suspend	Suspend	Suspend
12	4	0	No	Yes	No
12	4	1	No	STFT>0.43	STFT<=0.43
12	4	2	No	STFT>1.5	STFT<=1.5
12	4	3	No	STFT>2.57	STFT<=2.57
12	4	4	No	STFT>3.65	STFT<=3.65
12	4	5	No	STFT>4.72	STFT<=4.72
12	4	6	No	STFT>5.79	STFT<=5.79
12	4	>=7	Suspend	Suspend	Suspend
12	5, 6	<=7	No	No	Yes
12	>=7	<=5	No	No	Yes & Eliminate

NOTE: "No. treated" is the total number of patients treated at the current dose level, "No. DLTs" is the number of patients who experienced DLT at the current dose level, "No. with data pending" denotes that number of patients whose DLT data are pending at the current dose level, "STFT" is the standardized total follow-up time for the patients with data pending, defined as the total follow-up time for the patients with data pending divided by the length of the DLT assessment window. "Y" represents "Yes," and "Y&Elim" represents "Yes & Eliminate." “Suspend” is “suspend accrual.” When a dose is eliminated, all higher doses should also be eliminated. At the dose level 1 (the higher dose), if the rule asks to escalation for the next cohort of patients, the new patients will stay at the current dose level. At the dose level 2(the lower dose level), if the rule asks for de-escalation for the next cohort of patients, we will need to check the Table 6-2 for the likelihood of stop the trial for toxicity.

Table 6-2. Stopping boundaries.

	1	2	3	4	5	6	7	8	9
Number of evaluable patients treated at the lowest dose	1	2	3	4	5	6	7	8	9
Stop the trial if # of DLT >=	NA	NA	2	3	3	4	4	4	5

Note: Stop the trial if the number of patients experiencing dose limiting toxicity at the lowest dose level exceeds the stopping boundaries listed in Table 2. # of DLT is the number of patients with at least 1 DLT and "NA" means that a trial cannot be stopped before treating 3 evaluable patients at the lowest dose

Phase II: The objective of Phase II is to preliminarily evaluate the efficacy and further evaluate safety of the combination at the RP2D. The phase II cohort will enroll patients using the established RP2D of DEC-C from the phase Ib portion in combination with standard doses of Nivolumab. Patients treated at the RP2D in Phase 1b will be included in analysis for the Phase 1I component of this study. All patients enrolled at the RP2D level and receiving at least 2 treatment cycles will be included in the efficacy and safety/tolerability endpoint analysis. Our results indicate that the objective response rate (ORR) to anti-PD1 in mucosal melanoma patients at our institution is 20%. We hypothesize ORR for the combination will be 43%, a 23% absolute improvement over the anti-PD1 treatment. Simon's 2-stage design will be used to evaluate the ORR (*28*). The null hypothesis that the ORR is 20% will be rejected if there are 3 or more response among the 11 patients. The trial will move on to enroll up to a total 19 patients. The design yields a type I error rate of 0.1 and power of 80% when the true response rate is 43%.

9.4.2 ANALYSIS OF THE PRIMARY EFFICACY ENDPOINT(S)

The efficacy analysis population consists of all subjects who receive any DEC-C treatment. RECIST will be used in this study for assessment of tumor response. While either CT or MRI may be utilized, as per RECIST, CT is the preferred imaging technique in this study. However, the optimal imaging technique should be determined by the treating physician. For the end point overall response rate (ORR), ORR will be summarized for patients on R2PD and 95% exact confidence interval provided.

9.4.3 ANALYSIS OF THE SECONDARY ENDPOINT(S)

The efficacy analysis population consists of all subjects who receive any DEC-C treatment. For the secondary endpoints of PFS and OS, Kaplan-Meier product-limit method will be used to estimate the PFS and OS and median time and associated 95% CI reported.

9.4.4 SAFETY ANALYSES

All patients who received any protocol therapy will be employed for safety analyses. Adverse Event (AE)/Safety analysis will be performed on all patients in the Full Analysis Set. Patient incidence of all treatment-emergent AEs will be tabulated by system organ class and preferred term. Tables of fatal AEs, serious AEs, treatment-related AEs, and AEs leading to withdrawal from investigation product will tabulated and provided. For select laboratory parameters, changes of laboratory values over time (e.g., change from baseline summary statistics), grade shifts in

laboratory values from baseline to worst on-study value, and grade 3 or higher laboratory toxicities will be summarized.

The ECG measurements from this study will be performed as per standard of care for routine safety monitoring. The MedDRA will be used to code adverse events and the NCI CTCAE version 5.0 will be used to grade severity of adverse events and laboratory toxicities.

Summary statistic will be provided for total number of subjects treated under each dose, average dose administered, and duration of treatment.

9.4.5 BASELINE DESCRIPTIVE STATISTICS

Descriptive summaries of demographic and baseline characteristics for all enrolled patients will be tabulated. Patients will be tabulated by dose level and overall and will be tabulated by anatomical location when possible.

9.4.6 PLANNED INTERIM ANALYSES

Safety data will be reviewed following the TITE-BOIN design for all the enrolled subjects for phase IB. There is no additional interim analysis of the safety data other than following the TITE-BOIN design. Sub-Group Analyses

No formal subgroup analysis is planned due to low number of total enrollment; however, analyses comparing responses among gender and anatomic location may be performed depending on final target enrollment.

9.4.7 TABULATION OF INDIVIDUAL PARTICIPANT DATA

The Full Analysis Set includes all patients in the As-Treated Population who have a baseline disease assessment and have the opportunity to be followed for at least 24 weeks at the time of the data cutoff (i.e., dosed at least 24 weeks prior to the time of the data cutoff). Individual participant data will be listed by measure and time point in tabular form.

9.4.8 EXPLORATORY ANALYSES

- **Tumor and circulating immune cell analysis:** Flow cytometry analysis of tumor cells and circulating immune cells (T cells including exhausted T cells and immunosuppressive Tregs, MDSCs, and NK cells) will be performed on serial patient blood samples and FNA biopsies as previously described.(*32*) This will allow us to compare pre- and post-treatment immune cell profiles to assess whether the combination of DEC-C and Nivolumab favorably alters immune cell profiles and if immune profiles correlate with patient responses. Cells recovered from flow cytometry analysis will be used to purify gDNA and RNA for subsequent correlative analyses.

- **Global methylation analysis:** Global methylation levels will be analyzed in peripheral blood mononuclear cells and tumor cells prior to and during treatment by

performing LINE-1 qRT-PCR on bisulfite-treated gDNA. This data will allow us to determine whether DEC-C and is inducing global hypo-methylation as expected.

- **RIG-I innate immune pathway analysis:** Gene expression will be analyzed in tumor cells prior to and during treatment by performing qRT-PCR analysis of RNA for a panel of 20 RIG-I pathway genes.

- **Plasma protein analysis:** Serial patient plasma samples will be analyzed using the Olink proteomics Immuno-Oncology detection panel to quantify the expression of 92 proteins including innate immune sensing pathway cytokines and proteins (TNFa, IL-6, IL-8, etc.) and other immune markers including NK ligands. This data will determine a) whether pre-treatment serum protein levels predict responses to, and b) are increased by the treatment with, the combination of DEC-C and Nivolumab. This data will be compared to Olink proteomics data for treatment-naïve and anti-PD1 only treated mucosal melanoma patients with samples in our melanoma biorepository.

10 SUPPORTING DOCUMENTATION AND OPERATIONAL CONSIDERATIONS

10.1 REGULATORY, ETHICAL, AND STUDY OVERSIGHT CONSIDERATIONS

10.1.1 INFORMED CONSENT PROCESS

10.1.1.1 CONSENT/ASSENT AND OTHER INFORMATIONAL DOCUMENTS PROVIDED TO PARTICIPANTS

Consent forms describing in detail the study agent, study procedures, and risks are given to the participant and written documentation of informed consent is required prior to starting intervention/ administering study product.

10.1.1.2 CONSENT PROCEDURES AND DOCUMENTATION

Informed consent process will be initiated prior to the individual's agreeing to participate in the study and continues throughout the individual's study participation. Extensive discussion of risks and possible benefits of participation will be provided to the participants and their families.

Consent forms will be IRB-approved, and the participant will be asked to read and review the document. The investigator will explain the research study to the participant and answer any questions that may arise. All participants will receive a verbal explanation in terms suited to their comprehension of the purposes, procedures, and potential risks of the study and of their rights as research participants. Participants will have the opportunity to carefully review the written consent form and ask questions prior to signing. The participants will have the opportunity to discuss the study with their surrogates or think about it prior to agreeing to

participate. The participant will sign the informed consent document prior to any procedures being done specifically for the study.

The participants may withdraw consent at any time throughout the course of the trial. A copy of the informed consent document will be given to the participants for their records. The rights and welfare of the participants will be protected by emphasizing to them that the quality of their medical care will not be adversely affected if they decline to participate in this study.

The study allows the inclusion of non-English speaking and non-reading participants. Witnesses to these consent processes will be individuals not associated with the trial and will not have a conflict of interest.

10.1.2 STUDY DISCONTINUATION AND CLOSURE

This study may be temporarily suspended or prematurely terminated if there is sufficient reasonable cause. Written notification, documenting the reason for study suspension or termination, will be provided by the suspending or terminating party to the study PI and sponsors. If the study is prematurely terminated or suspended, the PI will promptly inform the IRB and will provide the reason(s) for the termination or suspension.

Circumstances that may warrant termination or suspension include, but are not limited to:

- Determination of unexpected, significant, or unacceptable risk to participants.
- Demonstration of efficacy that would warrant stopping.
- Insufficient compliance to protocol requirements.
- Data that are not sufficiently complete and/ or evaluable.
- Determination of futility.

Study may resume once concerns about safety, protocol compliance, and data quality are addressed and satisfy the sponsor, IRB, DSMC, and/ or FDA.

The End of Treatment (EOT) and Follow-up visit procedures are listed in Section 1.3 (*Schedule of Events*). Additionally, all subjects will be followed for overall survival until death, withdrawal of consent, or the end of the study, whichever occurs first.

10.1.3 CONFIDENTIALITY AND PRIVACY

Participant confidentiality is strictly held in trust by the participating investigators, their staff, and the sponsor-investigator(s) and their agents. This confidentiality is extended to cover testing of biological samples and genetic tests in addition to the clinical information relating to participants. Therefore, the study protocol, documentation, data, and all other information generated will be held in strict confidence. No information concerning the study, or the data, will be released to any unauthorized third party without prior written approval of the sponsor.

The study monitor, other authorized representatives of the sponsor-investigator, representatives of the IRB or pharmaceutical company supplying study product may inspect all documents and

records required to be maintained by the investigator, including but not limited to, medical records (office, clinic, or hospital) and pharmacy records for the participants in this study. The clinical study site will permit access to such records.

The study participant's contact information will be securely stored at each clinical site for internal use during the study. At the end of the study, all records will continue to be kept in a secure location for as long a period as dictated by local IRB and Institutional regulations.

Study subject research data, which is for purposes of statistical analysis and scientific reporting, will be transmitted to and stored at the University of Colorado Cancer Center. This will not include the subject's contact or identifying information. Rather, individual subjects and their research data will be identified by a unique study identification number. The study data entry and study management systems used by the University of Colorado Cancer Center research staff will be secured and password protected. At the end of the study, all study databases will be de-identified and archived at the University of Colorado Cancer Center.

10.1.4 FUTURE USE OF STORED SPECIMENS OR DATA

- **Intended Use:** Samples and data collected under this protocol may be used to study mucosal melanoma. No genetic testing will be performed.

- **Storage:** Access to stored samples will be limited to authorized personnel using secure laboratory access. Samples and data will be stored using codes assigned by the investigators. Data will be kept in password-protected computers. Only investigators will have access to the samples and data.

- **Tracking:** Data will be tracked using the REDCap database.

- **Disposition at completion of the study**: All stored samples will be sent to the University of Colorado Melanoma Biorepository. Study participants who request destruction of samples will be notified of compliance with such request and all supporting details will be maintained for tracking.

Data collected for this study will be analyzed and stored at the University of Colorado Cancer Center. Clinical data will be collected during each visit by the clinical provider and stored in a locked office or cabinet with access limited to the study coordinator and/or data manager. Age, gender, pathologic features of the melanoma, co-morbid conditions, and concomitant medications will be recorded. Study data will be collected and managed using REDCap. REDCap is a secure web application designed to support data capture for research studies, providing user-friendly web-based case report forms, real-time data entry validation (e.g., for data types and range checks), audit trails and a de-identified data export mechanism to common statistical packages. The data system includes password protection and internal quality checks.

After the study is completed, the de-identified, archived data may be transmitted to and stored in a password protected database for use by other researchers including those outside of the study. Permission to transmit data will be included in the informed consent.

With the participant's approval and as approved by local IRBs, de-identified biological samples will be stored at the University of Colorado Melanoma Biorepository. These samples could be used for research into the causes of mucosal melanoma, its complications and other conditions for which individuals with mucosal melanoma are at increased risk, and to improve treatment. The University of Colorado Melanoma Biorepository will also be provided with a code-link that will allow linking the biological specimens with the phenotypic data from each participant, maintaining the masking of the identity of the participant.
During the conduct of the study, and individual participant can choose to withdraw consent to have biological specimens stored for future research. However, withdrawal of consent with regard to specimen storage will not be possible after the study is completed.

When the study is completed, access to study data and/ or samples will be provided through the University of Colorado Melanoma Biorepository.

10.1.5 SAFETY OVERSIGHT

The sponsor investigator will be responsible for monitoring the trial per the trial monitoring plan, in addition to overseeing the safety and efficacy of the trial including any specimens collected, executing the data and safety monitoring (DSM) plan, and complying with all reporting requirements to local and federal authorities. This oversight will be accomplished through additional oversight from the Data and Safety Monitoring Committee (DSMC) at the University of Colorado Cancer Center (CU Cancer Center). The DSMC is responsible for ensuring data quality and study participant safety for all clinical studies at the CU Cancer Center, which is the coordinating institution of this trial. A summary of the DSMC's activities is as follows:

- Conduct of internal audits
- Ongoing review of all serious adverse events (SAEs) and unanticipated problems (UAPs)
- May submit recommendations for corrective actions to the CU Cancer Center's Executive Committee

Per the CU Cancer Center Institutional DSM Plan, SAEs and UAPs are reported to the DSMC, IRB and the sponsor investigator per protocol. All SAEs and UAPs are to be reported to the DSMC within 7 (for fatal or life-threatening events) or 15 (non-life-threatening events) calendar days of the sponsor investigator receiving notification of the occurrence.

Each subject's treatment outcomes will be discussed by the site PI and appropriate staff at regularly scheduled meetings. Data regarding number of subjects, significant toxicities, dose modifications, and treatment responses will be discussed and documented in the meeting's minutes.

The sponsor investigator will provide a DSM progress report to the CU Cancer Center DSMC on a recurring basis (either every six or twelve months based on DSMC vote). The DSM report will

include a protocol summary, current enrollment numbers, summary of toxicity data to include specific SAEs, UAPs and AEs, any dose modifications, all protocol deviations, and protocol amendments. The DSM progress report submitted to the DSMC will also include, if applicable, the results of any efficacy data analysis conducted. Results and recommendations from the review of this progress report by the DSMC will then be provided to the sponsor investigator in a DSMC review letter. The sponsor investigator is then responsible for ensuring this letter is submitted to the site's IRB of record at the time of IRB continuing review.

will include a protocol summary; current enrollment numbers; summary of toxicity data to include specific SAEs, UAPs and AEs; any dose modifications; all protocol deviations; and protocol amendments. The DSM report submitted to the DSMC will also include, if applicable, the results of any efficacy data analysis conducted. Results and recommendations from the review of this six month report by the DSMC will then be provided to the sponsor investigator in a DSMC review letter. The sponsor-investigator is then responsible for ensuring this letter is submitted to the site's IRB of record at the time of IRB continuing review.

10.1.6 CLINICAL MONITORING

Clinical site monitoring will be conducted to ensure that the rights and well-being of human participants are protected, that the reported trial data are accurate, complete, and verifiable, and that the conduct of the trial is in compliance with the currently approved protocol/ amendment(s), with GCP, and with applicable regulatory requirement(s).

Monitoring for this study will be performed by a CU Cancer Center Clinical Monitor in accordance with the clinical monitoring plan (CMP), incorporated herein by reference. The CMP describes in detail who will conduct the monitoring, at what frequency monitoring will be done, at what level of detail monitoring will be performed, and the distribution of the monitoring reports.

10.1.7 QUALITY ASSURANCE AND QUALITY CONTROL

Quality Control (QC) procedures will be implemented beginning with the data entry system and data QC checks that will be run on the database will be generated. Any missing data or data anomalies will be communicated to the site(s) for clarification/ resolution.

Following written SOPs, the study monitor will verify that the clinical trial is conducted and data are generated, documented (recorded), and reported in compliance with the protocol, GCP, and the applicable regulatory requirements (e.g., Good Laboratory Practices [GLP], Good Manufacturing Practices [GMP]).

The investigational site will provide direct access to all trial-related sites, source data/ documents, and reports for the purpose of monitoring and auditing by the DSMC audit team, and inspection by local and regulatory authorities.

10.1.8 DATA HANDLING AND RECORD KEEPING

10.1.8.1 DATA COLLECTION AND MANAGEMENT RESPONSIBILITIES

Data collection is the responsibility of the clinical trial staff at the site under the supervision of the site PI. The PI is responsible for ensuring the accuracy, completeness, legibility, and timeliness of the data reported.

All source documents should be completed in a neat, legible manner to ensure accurate interpretation of data. When making changes or corrections, cross out the original entry with a single line, and initial and date the change. DO NOT ERASE, OVERWRITE, OR USE CORRECTION FLUID OR TAPE ON THE ORIGINAL.

Copies of the electronic CRF (eCRF) will be provided for use as source documents and maintained for recording data for each participant enrolled in the study. Data reported in the eCRF derived from source documents should be consistent with the source documents or the discrepancies should be explained and captured in a progress note and maintained in the participant's official electronic study record.

Clinical data (including AEs, concomitant medications, and expected adverse reactions data) and clinical laboratory data will be entered into the REDCap database, OR a 21 CFR Part 11-compliant data capture system provided by the University of Colorado Cancer Center. The data system includes password protection and internal quality checks, such as automatic range checks, to identify data that appear inconsistent, incomplete, or inaccurate. Clinical data will be entered directly from the source documents.

10.1.8.2 STUDY RECORDS RETENTION

Study documents should be retained for a minimum of 2 years after the last approval of an investigational marketing application and until there are no pending or contemplated marketing applications or until at least 2 years have elapsed since the formal discontinuation of clinical development of the investigational product. These documents should be retained for a longer period, however, if required by local regulations or institutional policies. No records will be destroyed without the written consent of the sponsor, if applicable. It is the responsibility of the sponsor to inform the PI when these documents no longer need to be retained.

10.1.9 PROTOCOL DEVIATIONS

A protocol deviation is any noncompliance with the clinical trial protocol, GCP, or SOP requirements. The noncompliance may be either on the part of the participant, the investigator, or the study site staff. As a result of deviations, corrective actions are to be developed by the site and implemented promptly. These practices are consistent with ICH E6, sections:

- 4.5 Compliance with Protocol, sections 4.5.1, 4.5.2, and 4.5.3.
- 5.1 Quality Assurance and Quality Control, section 5.1.1.
- 5.20 Noncompliance, sections 5.20.1 and 5.20.2.

It is the responsibility of the study team to use continuous vigilance to identify and report deviations. All deviations must be addressed in study source documents, reported as required by COMIRB UAP Reporting Guidelines. Protocol deviations must be sent to the local IRB per institutional guidelines. The site PI/study staff is responsible for knowing and adhering to their IRB requirements. Further details about the handling of protocol deviations will be included in the SOP and/or study procedures manual.

10.1.10 PUBLICATION AND DATA SHARING POLICY

This study will ensure that the public has access to the published results of this research.

As required, either for publication (the ICMJE or other publication policy), or according to U.S. regulations (Section 801 of the Food and Drug Administration Amendments Act of 2007) this clinical trial will be registered in a public trials registry including ClinicalTrials.gov, which is sponsored by the National Library of Medicine, and the NCI CTRP Registry for cancer clinical trials.

10.1.11 CONFLICT OF INTEREST POLICY

Independence of this study from any actual or perceived influence, such as by the pharmaceutical industry, is critical. Any actual conflict of interest of persons who have a role in the design, conduct, analysis, publication, or any aspect of this trial will be disclosed and managed by the University of Colorado Denver's Office of Regulatory Compliance Conflict of Interest and Commitment Management (COIC) program. Persons with a perceived conflict of interest will have such conflicts managed in a way that is appropriate to their participation in the trial. Conflict of Interest management plans are project-specific and are reviewed at least annually. The University of Colorado Denver has integrated the institutional conflict of interest management program with its existing program.

11 REFERENCES

1. P. L. Triozzi *et al.*, Differential effects of low-dose decitabine on immune effector and suppressor responses in melanoma-bearing mice. *Cancer Immunol Immunother* **61**, 1441-1450 (2012).
2. G. Yu *et al.*, Low-dose decitabine enhances the effect of PD-1 blockade in colorectal cancer with microsatellite stability by re-modulating the tumor microenvironment. *Cell Mol Immunol* **16**, 401-409 (2019).
3. C. Fazio *et al.*, Immunomodulatory Properties of DNA Hypomethylating Agents: Selecting the Optimal Epigenetic Partner for Cancer Immunotherapy. *Front Pharmacol* **9**, 1443 (2018).
4. B. J. Schmiedel *et al.*, Azacytidine impairs NK cell reactivity while decitabine augments NK cell responsiveness toward stimulation. *Int J Cancer* **128**, 2911-2922 (2011).
5. A. E. Chang, L. H. Karnell, H. R. Menck, The National Cancer Data Base report on cutaneous and noncutaneous melanoma: a summary of 84,836 cases from the past decade. The American College of Surgeons Commission on Cancer and the American Cancer Society. *Cancer* **83**, 1664-1678 (1998).
6. M. Mihajlovic, S. Vlajkovic, P. Jovanovic, V. Stefanovic, Primary mucosal melanomas: a comprehensive review. *Int J Clin Exp Pathol* **5**, 739-753 (2012).
7. D. Kuk *et al.*, Prognosis of Mucosal, Uveal, Acral, Nonacral Cutaneous, and Unknown Primary Melanoma From the Time of First Metastasis. *Oncologist* **21**, 848-854 (2016).
8. S. P. D'Angelo *et al.*, Efficacy and Safety of Nivolumab Alone or in Combination With Ipilimumab in Patients With Mucosal Melanoma: A Pooled Analysis. *J Clin Oncol* **35**, 226-235 (2017).
9. A. N. Shoushtari *et al.*, The efficacy of anti-PD-1 agents in acral and mucosal melanoma. *Cancer* **122**, 3354-3362 (2016).
10. M. A. Postow *et al.*, Ipilimumab for patients with advanced mucosal melanoma. *Oncologist* **18**, 726-732 (2013).
11. J. D. Wolchok *et al.*, Nivolumab plus ipilimumab in advanced melanoma. *N Engl J Med* **369**, 122-133 (2013).
12. K. Namikawa *et al.*, Efficacy and safety of nivolumab in combination with ipilimumab in Japanese patients with advanced melanoma: An open-label, single-arm, multicentre phase II study. *Eur J Cancer* **105**, 114-126 (2018).
13. O. Hamid *et al.*, Safety and tumor responses with lambrolizumab (anti-PD-1) in melanoma. *N Engl J Med* **369**, 134-144 (2013).
14. A. Betof Warner *et al.*, Long-Term Outcomes and Responses to Retreatment in Patients With Melanoma Treated With PD-1 Blockade. *J Clin Oncol*, JCO1901464 (2020).
15. S. Bek *et al.*, Targeting intrinsic RIG-I signaling turns melanoma cells into type I interferon-releasing cellular antitumor vaccines. *Oncoimmunology* **8**, e1570779 (2019).
16. L. Such *et al.*, Targeting the innate immunoreceptor RIG-I overcomes melanoma-intrinsic resistance to T cell immunotherapy. *J Clin Invest* **130**, 4266-4281 (2020).
17. S. Heidegger *et al.*, RIG-I activation is critical for responsiveness to checkpoint blockade. *Sci Immunol* **4**, (2019).
18. K. B. Chiappinelli *et al.*, Inhibiting DNA Methylation Causes an Interferon Response in Cancer via dsRNA Including Endogenous Retroviruses. *Cell* **164**, 1073 (2016).
19. S. Dhillon, Decitabine/Cedazuridine: First Approval. *Drugs* **80**, 1373-1378 (2020).

20. G. G. Chabot, J. Bouchard, R. L. Momparler, Kinetics of deamination of 5-aza-2'-deoxycytidine and cytosine arabinoside by human liver cytidine deaminase and its inhibition by 3-deazauridine, thymidine or uracil arabinoside. *Biochem Pharmacol* **32**, 1327-1328 (1983).
21. D. Ferraris *et al.*, Design, synthesis, and pharmacological evaluation of fluorinated tetrahydrouridine derivatives as inhibitors of cytidine deaminase. *J Med Chem* **57**, 2582-2588 (2014).
22. S. Coral *et al.*, Phenotypic and functional changes of human melanoma xenografts induced by DNA hypomethylation: immunotherapeutic implications. *J Cell Physiol* **207**, 58-66 (2006).
23. H. A. Tawbi *et al.*, Safety and efficacy of decitabine in combination with temozolomide in metastatic melanoma: a phase I/II study and pharmacokinetic analysis. *Ann Oncol* **24**, 1112-1119 (2013).
24. A. M. Di Giacomo *et al.*, Guadecitabine Plus Ipilimumab in Unresectable Melanoma: The NIBIT-M4 Clinical Trial. *Clin Cancer Res* **25**, 7351-7362 (2019).
25. E. M. Burton *et al.*, A phase II study of oral azacitidine (CC-486) in combination with pembrolizumab (PEMBRO) in patients (pts) with metastatic melanoma (MM). *Journal of Clinical Oncology* **37**, 9560-9560 (2019).
26. H. Fan *et al.*, Low-dose decitabine-based chemoimmunotherapy for patients with refractory advanced solid tumors: a phase I/II report. *J Immunol Res* **2014**, 371087 (2014).
27. Y. Yuan and S. Liu. BOIN: Bayesian Optimal Interval (Boin) Deisgn for Single-Agent and Drug-Combination Phase I Clinical Trials. *https://cran.r-project.org/package=BOIN* (2018).
28. R. Simon, Optimal two-stage designs for phase II clinical trials. *Control Clin Trials* **10**, 1-10 (1989).
29. C. S. Qian *et al.*, PD1 inhibitor in combination with 5-azacytidine and low-dose DLI for the successful treatment of AML patients who relapsed after transplantation. *Bone Marrow Transplant*, (2020).
30. E. Jabbour *et al.*, Randomized phase 2 study of low-dose decitabine vs low-dose azacitidine in lower-risk MDS and MDS/MPN. *Blood* **130**, 1514-1522 (2017).
31. J. Larkin *et al.*, Five-Year Survival with Combined Nivolumab and Ipilimumab in Advanced Melanoma. *N Engl J Med* **381**, 1535-1546 (2019).
32. P. H. Lizotte *et al.*, Fine needle aspirate flow cytometric phenotyping characterizes immunosuppressive nature of the mesothelioma microenvironment. *Sci Rep* **6**, 31745 (2016).
33. S. Liu and Y. Yuan. Bayesian optimal interval designs for phase I clinical trials. *Journal of the Royal Statistical Society* Series C, **64**, 507-523 (2015).
34. Y. Zhou *et al.*, Bayesian Optimal Interval (BOIN) Design for Phase I Clinical Trials. (V2.6.0.0) [Computer software]. Available at *https://www.trialdesign.org/* (2021).
