# Supplementary material for: Epigenetic Priming with Oral Decitabine/Cedazuridine Combined with Nivolumab in Unresectable or Metastatic Mucosal Melanoma: Clinical and Correlative Findings from a Phase Ib/II Trial
Source: Cancers (Basel). 2026 Oct 1;18(19):3172. doi: 10.3390/cancers18193172 (PMC13664269; doi:10.3390/cancers18193172)
Supplement: Supplementary file 1 [file cancers-18-03172-s001.zip › Supplementary File S2_09.30.pdf]

CONSORT-DEFINE downloadable checklist

Recommended checklist items to consider in an early phase dose-finding clinical trial report from CONSORT 2010 and CONSORT-DEFINE checklists[^]

Please cite as: Yap C, Solovyeva O, de Bono J, et al. Enhancing reporting quality and impact of early phase dose-finding clinical trials: CONSORT Dose-finding Extension (CONSORT-DEFINE) guidance. *BMJ* 2023;383:e076387. doi:10.1136/bmj-2023-076387

Category and section	Standard CONSORT 2010 checklist item		CONSORT-DEFINE checklist item for EPDF Trials		Addressed on Page No[¤]
	**Item No**	**CONSORT 2010**	**Item No**	**CONSORT-DEFINE**	
**Title and abstract**					
	1a	Identification as a randomised trial in the title	1a†	Identification as an early phase dose-finding (eg, first-in-human, dose escalation or de-escalation, phase 1, phase 1/2, expansion, dose titration) and, if applicable, randomised trial in the title or abstract	1-2,
	1b	Structured summary of trial design, methods, results, and conclusions (for specific guidance, see CONSORT for abstracts)	1b	Structured summary of trial design, methods, results, and conclusions (for specific guidance, see CONSORT-DEFINE for abstracts)	2
**Introduction**					
Background and objectives	2a	Scientific background and explanation of rationale	2a.1†	Description of research question(s) and justification for undertaking the trial, including summary of relevant clinical studies (published and unpublished) examining benefits and harms for each intervention	2-3
			2a.2*	Summary of key findings from relevant non-clinical or preclinical research	2-3
			2a.3*	Summary of findings from previously generated preclinical and translational studies to support any planned biomarker substudies (where applicable)	2-3
	2b	Specific objectives or hypotheses	2b†	Specific objectives (eg, relating to safety, activity, pharmacokinetics, pharmacodynamics, recommended dose(s))	4-5

Category and section	Standard CONSORT 2010 checklist item		CONSORT-DEFINE checklist item for EPDF Trials		Addressed on Page No¤
	Item No	CONSORT 2010	Item No	CONSORT-DEFINE	
**Methods**					
Trial design	3a	Description of trial design (such as parallel, factorial) including allocation ratio	3a.1†	Description of trial design elements, such as dose escalation or de-escalation strategy, number of treatment groups, allocation ratio if relevant, and details of any prespecified trial adaptations	3-4
			3a.2*	Trial design schema to show the flow of major transition points (eg, dose escalation to dose expansion, phase 1 to phase 2, single ascending dose to multiple ascending dose)	4
			3a.3*	Statistical methods or rationale underpinning the trial design	6-7
			3a.4*	Starting dose(s) with rationale	3-4
			3a.5*	Range of planned dose levels with rationale	3-4
			3a.6*	Presentation of planned dose levels (eg, as a diagram, table, or infographic), where applicable	N/A
			3a.7*	Skipping of dose level(s), if applicable	N/A
			3a.8*	Planned cohort size(s) (eg, fixed, flexible, adaptive)	3-4
			3a.9*	Dose allocation method within a dose level (including sequence and interval between dosing of participants, eg, sentinel or staggered dosing)	3-4
			3a.10*	Dose expansion cohort(s), if applicable, with rationale	N/A
			3a.11*	Criteria for progression to the next part of the trial (eg, phase 1 to phase 2, single ascending dose to multiple ascending dose), where applicable	4

Category and section	Standard CONSORT 2010 checklist item		CONSORT-DEFINE checklist item for EPDF Trials		Addressed on Page No¤
	Item No	CONSORT 2010	Item No	CONSORT-DEFINE	
	3b	Important changes to methods after trial commencement (such as eligibility criteria), with reasons	3b†	Important changes to the design or methods after trial commencement (eg, insertion of unplanned additional doses) outside the scope of the prespecified adaptive design features, with reasons	N/A
Participants	4a	Eligibility criteria for participants	4a		3
	4b	Settings and locations where the data were collected	4b		3
Interventions	5	The interventions for each group with sufficient details to allow replication, including how and when they were actually administered	5a†	Interventions for each dose level (within each group) with sufficient details to allow replication, including administration route and schedule showing how and when they were actually administered	3-5
			5b*	Criteria for dose discontinuation, dose modifications, and dosing delays of allocated interventions for a given trial participant (eg, dose change in response to harms, participant request, or improving or worsening disease)	4
Outcomes	6a	Completely defined prespecified primary and secondary outcome measures, including how and when they were assessed	6a†	Primary and secondary outcomes, including the specific measurement variable, analysis metric, method of aggregation, and time point for each outcome. Explanation of the clinical relevance of chosen outcomes is strongly recommended. Any other outcomes used to inform prespecified adaptations should be described with the rationale	5
	6b	Any changes to trial outcomes after the trial commenced, with reasons	6b†	Any unplanned changes to trial outcomes after the trial commenced, with reasons	4

Category and section	Standard CONSORT 2010 checklist item		CONSORT-DEFINE checklist item for EPDF Trials		Addressed on Page No¤
	Item No	CONSORT 2010	Item No	CONSORT-DEFINE	
Sample size	7a	How sample size was determined	7a†	Estimated number of participants (minimum, maximum, or expected range) needed to address trial objectives and how it was determined, including clinical and statistical assumptions supporting any sample size and operating characteristics	3-4 & 6-7
	7b	When applicable, explanation of any interim analyses and stopping guidelines	7b†	Prespecified interim decision making criteria or rules that guided the trial adaptation process (eg, dosing decision to (de-)escalate); prespecified and actual timing and frequency of interim data reviews and the information to inform trial adaptations	N/A
Randomization (if applicable)					
Sequence generation	8a	Method used to generate the random allocation sequence	8a		N/A, not a randomised trial
	8b	Type of randomisation; details of any restriction (such as blocking and block size)	8b†	Type of randomisation; details of any restrictions (such as blocking and block size); any prespecified adaptive assignment rules or algorithm leading to adjustments in the allocation ratio, including timing and frequency of updates; any changes to the allocation rule following trial adaptation decisions	N/A, not a randomised trial
Allocation concealment mechanism	9	Mechanism used to implement the random allocation sequence (such as sequentially numbered containers), describing any steps taken to conceal the sequence until interventions were assigned	9		N/A, not a randomised trial
Implementation	10	Who generated the random allocation sequence, who enrolled participants, and who assigned participants to interventions	10		N/A, not a randomised trial

Category and section	Standard CONSORT 2010 checklist item		CONSORT-DEFINE checklist item for EPDF Trials		Addressed on Page No¤
	**Item No**	**CONSORT 2010**	**Item No**	**CONSORT-DEFINE**	
Blinding	11a	If done, who was blinded after assignment to interventions (eg, participants, care providers, and how	11a		N/A
	11b	If relevant, description of the similarity of interventions	11b		N/A
Statistical methods	12a	Statistical methods used to compare groups for primary and secondary outcomes	12a.1†	Statistical methods for primary and secondary outcomes and any other outcomes used to make prespecified adaptations	6-7
			12a.2*	For the implemented adaptive design features, statistical methods used for estimation (eg, safety, dose(s), treatment effects) and to make inferences	N/A
	12b	Methods for additional analyses, such as subgroup analyses and adjusted analyses	12b†	Statistical methods for additional analyses (eg, subgroup and adjusted analyses, pharmacokinetics or pharmacodynamics, biomarker correlative analyses)	6-7
			12c*	Analysis population(s) (eg, evaluable population for dose-finding, safety population)	3-4 & 6-7
			12d*	Strategies for handling intercurrent events occurring after treatment initiation (eg, how dosing adjustments were handled) that can affect either the interpretation or the existence of the measurements associated with the clinical question of interest, and any methods to handle missing data	3-4 & 6-7
**Results**					

Category and section	Standard CONSORT 2010 checklist item		CONSORT-DEFINE checklist item for EPDF Trials		Addressed on Page No¤
	Item No	CONSORT 2010	Item No	CONSORT-DEFINE	
Participant flow (a diagram is strongly recommended)	13a	For each group, the numbers of participants who were randomly assigned, received intended treatment, and were analysed for the primary outcome	13a†	For each group, the number of participants who were assigned to each dose level at each interim analysis (eg, for dosing decisions), received intended treatment, and were analysed for the primary outcome and, if applicable, any other outcomes used to inform prespecified adaptations	7
	13b	For each group, losses and exclusions after randomisation, together with reasons	13b†	For each group, losses and exclusions after allocation to each dose level, together with reasons	7
Recruitment	14a§	Dates defining the periods of recruitment and follow-up	14a§		7
	14b§	Why the trial ended or was stopped	14b§		8
			14c*	Trial adaptation decisions made (including on what basis they were made, and when) in light of the prespecified decision making criteria and observed accrued data	8
Baseline data	15	A table showing baseline demographic and clinical characteristics for each group	15†	Baseline demographic and clinical characteristics across each dose level within each group, where appropriate	7-8
Numbers analysed	16	For each group, number of participants (denominator) included in each analysis and whether the analysis was by original assigned groups	16†	For each group, the number of participants (denominator) included in each analysis across each dose level, and whether the analysis was by original assigned interventions	N/A
Outcomes and estimation	17a	For each primary and secondary outcome, results for each group, and the estimated effect size and its precision (such as 95% confidence interval)	17a†	For each primary and secondary outcome, results for each dose level within each group, and the estimated effect size and its precision, if applicable	N/A

Category and section	Standard CONSORT 2010 checklist item		CONSORT-DEFINE checklist item for EPDF Trials		Addressed on Page No¤
	Item No	CONSORT 2010	Item No	CONSORT-DEFINE	
	17b§	For binary outcomes, presentation of both absolute and relative effect sizes is recommended	17b§		N/A
			17c*	Report interim results used to inform interim decision making such as dose escalation, de-escalation, or staying at the same dose	N/A
Ancillary analyses	18	Results of any other analyses performed, including subgroup analyses and adjusted analyses, distinguishing prespecified from exploratory	18		N/A
Harms	19	All important harms or unintended effects in each group (for specific guidance, see CONSORT for harms (1))	19†	All important harms (eg, adverse events or effects, toxicities) reported by dose level in each group (for specific guidance, see CONSORT for harms (2))	8-9
**Discussion**					
Limitations	20	Trial limitations, addressing sources of potential bias, imprecision, and, if relevant, multiplicity of analyses	20		15
Generalizability	21	Generalisability (external validity, applicability) of the trial findings	21		15
Interpretation	22	Interpretation consistent with results, balancing benefits and harms, and considering other relevant evidence	22		12-15
**Other information**					
Registration	23	Registration number and name of trial registry	23		2-3
Protocol	24	Where the full trial protocol can be accessed, if available	24		Supplementary File S1 pages 10-12

Category and section	Standard CONSORT 2010 checklist item		CONSORT-DEFINE checklist item for EPDF Trials		Addressed on Page No¤
	Item No	CONSORT 2010	Item No	CONSORT-DEFINE	
Funding	25	Sources of funding and other support (such as supply of drugs), role of funders	25		16 (manuscript)
Data monitoring			26a*	Composition of any decision making or safety review committee or group; summary of its role and reporting structure; statement of whether it is independent from the sponsor and competing interests; and reference to where further details can be found (such as in a charter or protocol)	50-60 (supplementary File S1)
			26b*	Description of who had access to interim results and made the interim and final decision to terminate the trial (or part(s) of the trial, eg, end of dose escalation), and measures to safeguard the confidentiality of interim information	1 (supplementary File S1)
Dissemination			27*	Specify, if applicable, whether and when results (such as safety and/or activity) were reported externally (eg, through scientific presentations, journal publication, or the trial website) while the trial (or part(s) of the trial) was still ongoing	N/A

CONSORT=CONonsolidated Standards Of Reporting Trials; DEFINE=Dose-finding Extension; EPDF=early phase dose-finding.

^ This checklist should be used in conjunction with the CONSORT explanation and elaboration document (3) for important clarifications on the checklist items. Empty items in the CONSORT-DEFINE column indicate no modification from the standard CONSORT item. CONSORT extensions for non-pharmacological treatments and outcomes might also be relevant (4). Note that the term "dose" in the checklist can be considered synonymous and used interchangeably with dosage, or dosing regimen (dose or schedule), or a unit dose.

¤ If the information is not provided in the primary paper, give details of where this information is available. This may include locations such as a published protocol or other published papers (provide citation details) or a website (provide the URL).

* New items that should only be applied in reference to CONSORT-DEFINE.

† Modified items that require reference to both CONSORT and CONSORT-DEFINE.

§ Item wording remains unchanged in reference to CONSORT, but additional CONSORT-DEFINE explanatory text has been provided to clarify additional considerations for early phase dose-finding trials.
