# Supplementary material for: Epigenetic Priming with Oral Decitabine/Cedazuridine Combined with Nivolumab in Unresectable or Metastatic Mucosal Melanoma: Clinical and Correlative Findings from a Phase Ib/II Trial
Source: Cancers (Basel). 2026 Oct 1;18(19):3172. doi: 10.3390/cancers18193172 (PMC13664269; doi:10.3390/cancers18193172)
Supplement: Supplementary file 1 [file cancers-18-03172-s001.zip › Supplementary Tables.pdf]

Supplementary Table S1 – Overview of flow cytometry panel

Marker	Fluor	Clone	Supplier
Viability	Ghost Dye Blue		
CD11b	PE-Cy7	ICRF44	BD
CD127 (IL-7Ra)	Spark NIR 685	A019D5	BioLegend
CD14	BUV805	M5E2	BD
CD15	BV605	W6D3	BD
CD16	BUV496	3G8	BD
CD161 (NK1.1)	RB744	HP-3G10	BD
CD19	BV650	SJ25C1	BioLegend
CD223 (LAG-3)*	PE-Fire 700	7H2C65	Biolegend
CD25	BUV563	M-A251	BD
CD279 (PD-1)*	RB613	EH12.1	BD
CD3	BB515	SK7	BD
CD300e	BUV661	UP-H1	BD
CD314 (NKG2D)	BUV737	1D11	BD
CD33*	RB705	WM53	BD
CD366 (TIM3)*	BV711	344823	BD
CD4	APC-Fire 810	SK3	BioLegend
CD45	BV570	HI30	BioLegend
CD56	R718	NCAM16.2	BD
CD64	BV480	10.1	BD
CD8	BUV395	RPA-T8	BD
CD96*	PE	NK92.39.rMAb	BD
FOXP3	RB780	236A/E7	BD
Granzyme B	Pacific Blue	GB11	BioLegend
LOX-1	APC		
MHC Class II*	RB545	G46-6	BD
MR1-Tet	BV421		
TCR gd	BUV615	11F2	BD
TCR Va7.2	BV785		

* gating was set using fluorescence minus one controls

Supplementary Table S2 – Summary Statistics of Log-Transformed Values of pre- and on-treatment circulating immune cells

CellType	Mean (Screen)	Mean (Tx)	Mean Diff (log-scale)	Fold Change	Pair Count	*P*value (Paried-T)
Live_CD45p/T_cells/Non-GD/Non-MAIT/CD8p/TIM3p_PD1p_Live_CD45p	-1.474	-3.395	-1.921	0.146	8	0.006
Live_CD45p/T_cells/Non-GD/Non-MAIT/CD8p/TIM3p_PD1p_Parent	-0.288	-2.069	-1.781	0.168	8	0.013
Live_CD45p/Non-B_Non-T/CD56Bright_CD16-/Immature_NK_Live_CD45p	-2.085	-2.303	-0.218	0.804	8	0.029
Live_CD45p/T_cells/Non-GD/Non-MAIT/CD4p/Treg/TIM3p_PD1p_Parent	1.465	0.008	-1.456	0.233	8	0.034
Live_CD45p/T_cells/Non-GD/MAIT/CD8neg_CD4p_Parent	0.179	0.616	0.436	1.547	8	0.083
Live_CD45p/Non-B_Non-T/NK_Live_CD45p	2.129	2.312	0.183	1.201	8	0.096
Live_CD45p/Non-B_Non-T/Non-NK/CD11bp/Non-MDSC/NOT_monocytes_Live_CD45p	-0.349	0.284	0.633	1.883	8	0.098
Live_CD45p/T_cells/Non-GD/MAIT/CD8p_CD4neg_Parent	4.427	4.378	-0.049	0.953	8	0.105
Live_CD45p/Non-B_Non-T/Non-NK/CD11bp/Non-MDSC/NOT_monocytes_Parent	1.021	1.533	0.513	1.67	8	0.118
Live_CD45p/Non-B_Non-T/Non-NK/CD11bp/Non-MDSC/Intermediate_Live_CD45p	0.709	1.106	0.397	1.488	8	0.121
Live_CD45p/T_cells/Non-GD/Non-MAIT/CD4p/Treg_Parent	0.888	1.125	0.238	1.268	8	0.128
Live_CD45p/Non-B_Non-T/Non-NK/CD11bp/MDSCs_Live_CD45p	-0.505	0.379	0.884	2.42	8	0.144
Live_CD45p/T_cells/Non-GD/Non-MAIT/CD4p/Treg/TIM3neg_PD1p_Parent	1.696	0.387	-1.309	0.27	8	0.144
Live_CD45p/T_cells_Live_CD45p	4.241	4.138	-0.103	0.902	8	0.176
Live_CD45p/T_cells/Non-GD/MAIT/CD8neg_CD4p_Live_CD45p	-6.051	-5.506	0.545	1.724	8	0.181
Live_CD45p/Non-B_Non-T/Non-NK/CD11bp/Non-MDSC/Intermediate_Parent	2.08	2.357	0.278	1.32	8	0.19
Live_CD45p/T_cells/Non-GD/Non-MAIT/CD4p/Treg_Live_CD45p	-0.367	-0.061	0.306	1.358	8	0.202
Live_CD45p/Non-B_Non-T/Non-NK/CD11bp/MDSCs/Mo-MDSC_Live_CD45p	-0.712	0.06	0.772	2.165	8	0.219
Live_CD45p/B_cells_Live_CD45p	1.935	1.739	-0.197	0.822	8	0.307
Live_CD45p/Non-B_Non-T/Non-NK/CD11bp/Non-MDSC/Nonclassical_Parent	2.258	1.943	-0.316	0.729	8	0.318
Live_CD45p/T_cells/Non-GD/Non-MAIT/CD8p/GZMBp_CD314p_Parent	3.995	4.174	0.178	1.195	8	0.321
PMN-MDSC_Live_cells	-2.318	-1.699	0.619	1.858	8	0.342
Live_CD45p/T_cells/Non-GD/Non-MAIT/CD8p_Live_CD45p	3.415	3.284	-0.131	0.877	8	0.368
Live_CD45p/T_cells/Non-GD/Non-MAIT/CD4p_Parent	3.76	3.927	0.167	1.181	8	0.439
Live_CD45p/Non-B_Non-T/Non-NK/CD11bp/Non-MDSC/Nonclassical_Live_CD45p	0.888	0.691	-0.197	0.821	8	0.446

Live_CD45p/Non-B_Non-T/CD56Dim_CD16+/Mature_NK_Live_CD45p	1.977	1.804	-0.172	0.842	8	0.556
Live_CD45p/Non-B_Non-T/Non-NK/CD11bp/MDSCs_Geometric_Mean_(Comp-PE-A_::_CD96)	6.659	6.72	0.061	1.063	8	0.561
Live_CD45p/Non-B_Non-T/Non-NK/CD11bp/Non-MDSC/Classical_Live_CD45p	2.832	3.03	0.198	1.218	8	0.592
Live_CD45p/Non-B_Non-T/Non-NK/CD11bp/Non-MDSC/Classical_Parent	4.202	4.281	0.079	1.082	8	0.644
Live_CD45p/T_cells/Non-GD/MAIT_Live_CD45p	-1.616	-1.519	0.097	1.102	8	0.684
Live_CD45p/T_cells/GD_T_cells_Live_CD45p	0.379	0.307	-0.072	0.93	8	0.706
Live_CD45p/T_cells/Non-GD/Non-MAIT/CD4p_Live_CD45p	3.357	3.419	0.062	1.064	8	0.776
Live_CD45p/T_cells/Non-GD/MAIT/CD8p_CD4neg_Live_CD45p	-1.801	-1.747	0.054	1.055	8	0.823
Live_CD45p/T_cells/Non-GD/Non-MAIT/CD8p_Parent	3.818	3.793	-0.025	0.975	8	0.84
Live_CD45p/T_cells/Non-GD/Non-MAIT/CD8p/GZMBp_CD314p_Live_CD45p	2.805	2.852	0.047	1.048	8	0.848

Supplementary Table S3 – Summary Statistics of Log-Transformed Values of circulating immune cells comparing responders and non-responders

	Partial Response (n = 2) Median			Progressive Disease (n = 4) Median			*P*-values Between [a]		
**CellType**	**Screen**	**During Tx**	**Diff.**	**Screen**	**During Tx**	**Diff.**	**Screen**	**During Tx**	**Diff.**
Live_CD45p/Non-B_Non-T/Non-NK/CD11bp/MDSCs_Geometric_Mean_(Comp-PE-A_::_CD96)	6.62	6.57	-0.05	6.56	6.75	0.19	1	1	0.13
Live_CD45p/T_cells/Non-GD/Non-MAIT/CD4p/Treg/TIM3neg_PD1p_Parent	3.21	-1.07	-4.28	0.92	-0.47	0.25	0.27	0.27	0.13
Live_CD45p/Non-B_Non-T/Non-NK/CD11bp/MDSCs/Mo-MDSC_Live_CD45p	0	-0.69	-0.69	-0.82	-0.06	1.06	0.53	0.53	0.27
Live_CD45p/Non-B_Non-T/Non-NK/CD11bp/MDSCs_Live_CD45p	0.16	-0.32	-0.48	-0.64	0.28	1.06	0.8	0.53	0.27
Live_CD45p/Non-B_Non-T/Non-NK/CD11bp/Non-MDSC/Intermediate_Live_CD45p	-0.06	0.62	0.68	1.14	1.05	0.06	0.27	0.8	0.27
Live_CD45p/Non-B_Non-T/Non-NK/CD11bp/Non-MDSC/Nonclassical_Live_CD45p	0.86	0.29	-0.57	0.91	0.8	0.3	1	0.8	0.27
Live_CD45p/Non-B_Non-T/Non-NK/CD11bp/Non-MDSC/Nonclassical_Parent	2.44	1.94	-0.5	2.16	2.19	0.47	0.53	0.8	0.27
Live_CD45p/T_cells/Non-GD/MAIT/CD8neg_CD4p_Parent	-0.29	-0.08	0.21	0.65	0.7	0.05	0.13	0.13	0.27
Live_CD45p/T_cells/Non-GD/MAIT/CD8p_CD4neg_Live_CD45p	0.24	-0.22	-0.46	-2.75	-2.6	0.15	0.13	0.13	0.27
Live_CD45p/T_cells/Non-GD/MAIT_Live_CD45p	0.5	0.04	-0.46	-2.6	-2.41	0.17	0.13	0.1	0.27
Live_CD45p/T_cells/Non-GD/Non-MAIT/CD4p/Treg/TIM3p_PD1p_Parent	2.22	-0.99	-3.21	0.72	-0.6	-0.42	0.8	0.8	0.27
Live_CD45p/T_cells/Non-GD/Non-MAIT/CD8p/TIM3p_PD1p_Live_CD45p	-0.78	-4.31	-3.53	-1.52	-4.07	-1.11	0.8	1	0.27
Live_CD45p/T_cells/Non-GD/Non-MAIT/CD8p/TIM3p_PD1p_Parent	0.54	-3.05	-3.58	-0.4	-2.79	-0.89	0.53	1	0.27
Live_CD45p/Non-B_Non-T/CD56Bright_CD16-/Immature_NK_Live_CD45p	-1.87	-2.2	-0.33	-2.09	-2.05	-0.01	1	1	0.53
Live_CD45p/Non-B_Non-T/Non-NK/CD11bp/Non-MDSC/Intermediate_Parent	1.51	2.27	0.76	2.34	2.51	0.15	0.53	0.8	0.53
Live_CD45p/T_cells/Non-GD/MAIT/CD8neg_CD4p_Live_CD45p	-4.41	-4.63	-0.22	-6.63	-5.97	0.17	0.1	0.13	0.53
Live_CD45p/T_cells/Non-GD/Non-MAIT/CD4p_Live_CD45p	3.71	3.27	-0.43	3.52	3.38	-0.01	0.53	0.8	0.53
PMN-MDSC_Live_cells	-1.41	-1.93	-0.52	-3.75	-2.53	0.91	0.48	0.53	0.53
Live_CD45p/T_cells/Non-GD/Non-MAIT/CD4p/Treg_Parent	1.24	1.75	0.51	0.91	1.23	0.19	0.27	0.27	0.8
Live_CD45p/T_cells/Non-GD/Non-MAIT/CD8p/GZMBp_CD314p_Live_CD45p	2.7	3.12	0.41	2.48	2.86	0.33	0.53	0.27	0.8
Live_CD45p/T_cells/Non-GD/Non-MAIT/CD8p_Parent	3.65	3.91	0.26	3.72	3.86	0.11	1	1	0.8
Live_CD45p/Non-B_Non-T/Non-NK/CD11bp/Non-MDSC/NOT_monocytes_Live_CD45p	-0.34	0.06	0.4	-0.52	0.19	0.73	0.8	0.8	0.8

Live_CD45p/Non-B_Non-T/Non-NK/CD11bp/Non-MDSC/NOT_monocytes_Parent	1.24	1.71	0.47	0.69	1.54	0.83	0.27	0.8	0.8
Live_CD45p/T_cells/Non-GD/Non-MAIT/CD4p_Parent	4.07	3.84	-0.23	4.04	3.91	-0.08	1	1	0.8
Live_CD45p/T_cells/Non-GD/Non-MAIT/CD8p/GZMBp_CD314p_Parent	4.02	4.38	0.36	4.17	4.22	0.13	1	0.8	0.8
Live_CD45p/T_cells/Non-GD/Non-MAIT/CD8p_Live_CD45p	3.29	3.34	0.05	3.21	3.33	0.12	0.8	1	0.8
Live_CD45p/T_cells_Live_CD45p	4.31	4.12	-0.19	4.2	4.17	-0.01	0.8	1	0.8
Live_CD45p/B_cells_Live_CD45p	2.15	1.87	-0.28	2.1	2.06	-0.04	0.8	0.8	1
Live_CD45p/Non-B_Non-T/CD56Dim_CD16+/Mature_NK_Live_CD45p	1.94	2.09	0.15	2.16	2.24	0.07	0.8	1	1
Live_CD45p/Non-B_Non-T/NK_Live_CD45p	2.01	2.21	0.21	2.29	2.36	0.24	0.8	0.8	1
Live_CD45p/Non-B_Non-T/Non-NK/CD11bp/Non-MDSC/Classical_Live_CD45p	2.79	2.62	-0.16	3.12	2.85	-0.21	0.13	0.53	1
Live_CD45p/Non-B_Non-T/Non-NK/CD11bp/Non-MDSC/Classical_Parent	4.36	4.28	-0.08	4.37	4.29	-0.12	1	0.8	1
Live_CD45p/T_cells/GD_T_cells_Live_CD45p	0.89	0.98	0.08	-0.28	-0.17	0.11	0.53	0.53	1
Live_CD45p/T_cells/Non-GD/MAIT/CD8p_CD4neg_Parent	4.35	4.34	-0.01	4.45	4.43	-0.01	0.53	1	1
Live_CD45p/T_cells/Non-GD/Non-MAIT/CD4p/Treg_Live_CD45p	0.34	0.41	0.08	-0.19	-0.07	0.28	0.27	0.53	1

p-values were calculated between PR and PD by Wilcoxon

Supplementary Table S4 – Summary Statistics of Original Scale Values of pre- and on-treatment tumor cells

Name	Median (Screen)	Median (Treatment)	Change (Median)	Pair Count
Live CD45+/T cells/Non-GD/ Non-MAIT/CD4+ (Freq of Live CD45+)	27.1	36.8	15.8	3
Live CD45+/T cells/Non-GD/ Non-MAIT/CD4+ (Freq of Parent)	49.5	62.2	14.1	3
Live CD45+/T cells/Non-GD/ Non-MAIT/CD8+ (Freq of Parent)	40	33.5	-8.6	3
Live CD45+/T cells/Non-GD/ Non-MAIT/CD8+ (Freq of Live CD45+)	28.7	21.2	-7.5	3
Live CD45+/T cells/Non-GD/ Non-MAIT/CD8+/ Q9: GRZM-B- , CD314+ (Freq of Parent)	60.6	65.1	-7.4	3
Live CD45+/T cells (Freq of Parent)	74.3	68.2	-6.1	3
Live CD45+/T cells/Non-GD/ Non-MAIT/CD8+/ Q2: TIM-3+ , PD-1+ (Freq of Parent)	13.6	22.9	-5.7	3
Live CD45+/T cells/Non-GD/ Non-MAIT/CD8+/ Q10: GRZM-B+ , CD314+ (Freq of Parent)	29.6	30	4.9	3
Live CD45+/T cells/Non-GD/ Non-MAIT/CD8+/ Q5: LAG-3- , CD127+ (Freq of Parent)	21.4	44	-3.9	3
Live CD45+ (Freq of Parent)	84.1	75.3	-3.7	3
Live CD45+/Non-B, Non-T/ NK (Freq of Live CD45+)	6.91	4.22	-2.65	3
Live CD45+/Non-B, Non-T/Non-NK/ CD11b+ (Freq of Live CD45+)	7.95	5.83	-2.12	3
Live CD45+/T cells/Non-GD/ Non-MAIT/CD4+/ Treg/Q2: TIM-3+ , PD-1+ (Freq of Parent)	22.2	17.1	-1.9	3
Live CD45+/B cells (Freq of Parent)	4.39	19.5	-1.07	3
Live CD45+/T cells/ GD T cells (Freq of Parent)	1.1	0.55	-0.3	3
Live CD45+/T cells/ GD T cells (Freq of Live CD45+)	0.58	0.49	-0.25	3
Live CD45+/T cells/Non-GD/ Non-MAIT/CD4+/ Treg (Freq of Parent)	2.39	5.28	-0.19	3
Live CD45+/T cells/Non-GD/ Non-MAIT/CD4+/ Treg (Freq of Live CD45+)	0.9	1.94	-0.15	3
Live CD45+/T cells/Non-GD/ MAIT cells (Freq of Parent)	0.81	0.46	-0.05	3
PMN-MDSC (Freq of Parent)	0.88	0.43	-0.03	3
Live CD45+/T cells/Non-GD/ MAIT cells (Freq of Live CD45+)	0.58	0.4	-0.01	3
Live CD45+/T cells/Non-GD/ Non-MAIT/CD4+/ Treg/Q1: TIM-3- , PD-1+ (Freq of Parent)	0	2.36	0	3

Supplementary Table S5 – Summary Statistics of Original Scale Values of tumor cells comparing responders and non-responders

	Partial Response (n = 1) Median			Progressive Disease (n = 2) Median		
**Name**	**Screen**	**During Tx**	**Diff.**	**Screen**	**During Tx**	**Diff.**
Live CD45+ (Freq of Parent)	87.6	83.9	-3.7	59.45	67.15	7.7
Live CD45+/B cells (Freq of Parent)	20.7	19.5	-1.2	3.62	11.34	7.72
Live CD45+/Non-B, Non-T/NK (Freq of Live CD45+)	6.91	8.59	1.68	7.57	3.52	-4.04
Live CD45+/Non-B, Non-T/Non-NK/CD11b+ (Freq of Live CD45+)	20.4	27.2	6.8	7.92	5.2	-2.71
Live CD45+/T cells (Freq of Parent)	48.7	38.8	-9.9	78.2	78.3	0.1
Live CD45+/T cells/GD T cells (Freq of Live CD45+)	0.54	0.78	0.24	0.74	0.41	-0.33
Live CD45+/T cells/GD T cells (Freq of Parent)	1.12	2.03	0.91	0.95	0.52	-0.43
Live CD45+/T cells/Non-GD/MAIT cells (Freq of Live CD45+)	1.03	1.64	0.61	0.5	0.29	-0.2
Live CD45+/T cells/Non-GD/MAIT cells (Freq of Parent)	2.15	4.33	2.18	0.66	0.36	-0.3
Live CD45+/T cells/Non-GD/Non-MAIT/CD4+ (Freq of Live CD45+)	27.1	22.5	-4.6	29.2	46	16.8
Live CD45+/T cells/Non-GD/Non-MAIT/CD4+ (Freq of Parent)	57.8	62.2	4.4	38	59.05	21.05
Live CD45+/T cells/Non-GD/Non-MAIT/CD4+/Treg (Freq of Live CD45+)	0.65	0.5	-0.15	1.76	3.8	2.04
Live CD45+/T cells/Non-GD/Non-MAIT/CD4+/Treg (Freq of Parent)	2.39	2.2	-0.19	8.04	7.79	-0.25
Live CD45+/T cells/Non-GD/Non-MAIT/CD4+/Treg/Q1: TIM-3- , PD-1+ (Freq of Parent)	6.34	2.36	-3.98	0	2.35	2.35
Live CD45+/T cells/Non-GD/Non-MAIT/CD4+/Treg/Q2: TIM-3+ , PD-1+ (Freq of Parent)	30.3	1.71	-28.59	14.13	18.7	4.57
Live CD45+/T cells/Non-GD/Non-MAIT/CD8+ (Freq of Live CD45+)	14.2	12.7	-1.5	32.95	25.1	-7.85
Live CD45+/T cells/Non-GD/Non-MAIT/CD8+ (Freq of Parent)	30.3	35.1	4.8	43.35	32.45	-10.9
Live CD45+/T cells/Non-GD/Non-MAIT/CD8+/Q10: GRZM-B+ , CD314+ (Freq of Parent)	21.2	30	8.8	31.7	31.25	-0.45
Live CD45+/T cells/Non-GD/Non-MAIT/CD8+/Q2: TIM-3+ , PD-1+ (Freq of Parent)	9.56	3.22	-6.34	21.1	25.95	4.85
Live CD45+/T cells/Non-GD/Non-MAIT/CD8+/Q5: LAG-3- , CD127+ (Freq of Parent)	65.9	62	-3.9	21.1	28.25	7.15
Live CD45+/T cells/Non-GD/Non-MAIT/CD8+/Q9: GRZM-B- , CD314+ (Freq of Parent)	74.2	65.1	-9.1	58.85	62.85	4
PMN-MDSC (Freq of Parent)	0.17	0.14	-0.03	1.25	1.56	0.31

Supplementary Table S6 – Summary Statistics of Log-Transformed Values of pre- and on-treatment plasma cytokines

Name	Mean (Screen)	Mean (Treatment)	Mean Diff (log-scale)	Fold Change	Pair Count	*P* value (Paried-T)
**IL-1β**	-1.829	-0.645	1.184	3.267	8	0.022
**IL-6**	0.364	1.003	0.638	1.893	8	0.054
**IL-8**	2.143	2.892	0.749	2.115	8	0.07
**TNF-α**	-0.229	0.608	0.837	2.309	8	0.18
**IFN-β**	-2.895	-1.781	1.114	3.047	8	0.257
**IFN-γ**	2.728	2.853	0.125	1.134	8	0.67
**IFN-α2a**	-1.982	-1.946	0.036	1.037	8	0.944

Supplementary Table S7 – Summary Statistics of Log-Transformed Values of plasma cytokines comparing responders and non-responders

	Partial Response (n = 2) Median			Progressive Disease (n = 4) Median			*P*-values Between [a]		
**Name**	**Screen**	**During Tx**	**Diff.**	**Screen**	**During Tx**	**Diff.**	**Screen**	**During Tx**	**Diff.**
**IFN-γ**	2.24	2.78	0.54	3.32	3.22	-0.43	0.13	0.8	0.27
**IL-1β**	-2.04	-1.2	0.84	-1.48	-1.42	0.19	1	1	0.53
**TNF-α**	-0.65	-0.02	0.63	0.13	0.36	0.05	0.53	0.53	0.53
**IFN-α2a**	-0.76	-0.13	0.63	-3.75	-3.33	-0.05	0.24	0.24	0.8
**IL-6**	-0.26	0.35	0.61	0.73	0.78	0.08	0.53	1	0.8
**IL-8**	1.8	2.71	0.91	1.89	2.4	0.52	1	0.53	0.8
**IFN-β**	-1.47	-0.34	1.13	-4.61	-3.03	1.58	0.22	0.24	1

p-values were calculated between PR and PD by Wilcoxon

Supplementary Table S8. Representativeness of Study Participants.

**Supplementary Table 8.** Representativeness of Study Participants	
**Cancer type(s)/subtype(s)/stage(s)/condition**	Melanoma, mucosal melanoma, unresectable stage III or stage IV
**Considerations related to:**	
**Sex**	Mucosal melanomas account for 0.8–3.7% of all melanomas diagnosed annually. Women are more likely to develop mucosal melanoma due to vulvovaginal mucosal involvement.
**Age**	The median age of diagnosis for melanoma is 65.
**Race/ethnicity**	While non-cutaneous melanomas, including mucosal melanoma, account for only 2-8% of melanoma cases in White populations, they represent the most common subtypes in Latin-American, African, and Asian populations.
**Geography**	While the incidence of cutaneous melanoma has increased, the incidence of mucosal melanoma appears to be stable over time. Incidence is highest in Latin-American, African, and Asian populations.
**Overall representativeness of this study**	The median age of our study participants was 64, which is similar to the values reported in the literature. All patient in our trial were female, and while mucosal melanoma is more common in female due to vulvovaginal involvement, this is higher than expected.<br><br>Race and ethnicity were not considered in the inclusion/exclusion criteria of the trial, and patients

	from three different ethnic backgrounds were included.<br><br>Our study population was limited to a single institution, and all participants were recruited at this institution.
